# Neutrophil diversity and function in health and disease

**DOI:** 10.1038/s41392-024-02049-y

**Published:** 2024-12-06

**Authors:** Fengyuan Zhang, Yidan Xia, Jiayang Su, Fushi Quan, Hengzong Zhou, Qirong Li, Qiang Feng, Chao Lin, Dongxu Wang, Ziping Jiang

**Affiliations:** 1 Department of Hand and Foot Surgery, Orthopedics Center, The First Hospital of Jilin University, Changchun, People’s Republic of China; 2 Institute of Translational Medicine, The First Hospital of Jilin University, Changchun, China; 3 Laboratory Animal Center, College of Animal Science, Jilin University, Changchun, China; 4 School of Grain Science and Technology, Jilin Business and Technology College, Changchun, China

**Keywords:** Inflammation, Inflammation

## Abstract

Neutrophils, the most abundant type of granulocyte, are widely recognized as one of the pivotal contributors to the acute inflammatory response. Initially, neutrophils were considered the mobile infantry of the innate immune system, tasked with the immediate response to invading pathogens. However, recent studies have demonstrated that neutrophils are versatile cells, capable of regulating various biological processes and impacting both human health and disease. Cytokines and other active mediators regulate the functional activity of neutrophils by activating multiple receptors on these cells, thereby initiating downstream signal transduction pathways. Dysfunctions in neutrophils and disruptions in neutrophil homeostasis have been implicated in the pathogenesis of numerous diseases, including cancer and inflammatory disorders, often due to aberrant intracellular signaling. This review provides a comprehensive synthesis of neutrophil biological functions, integrating recent advancements in this field. Moreover, it examines the biological roles of receptors on neutrophils and downstream signaling pathways involved in the regulation of neutrophil activity. The pathophysiology of neutrophils in numerous human diseases and emerging therapeutic approaches targeting them are also elaborated. This review also addresses the current limitations within the field of neutrophil research, highlighting critical gaps in knowledge that warrant further investigation. In summary, this review seeks to establish a comprehensive and multidimensional model of neutrophil regulation, providing new perspectives for potential clinical applications and further research.

## Introduction

Neutrophils, the most predominant type of granulocyte, comprise 40–70% of all human leukocytes.^1^ Approximately 100–200 billion neutrophils are generated daily in humans through hematopoiesis in the bone marrow.^2^ According to conventional estimates, the half-life of circulating neutrophils spans 4 to 18 h during homeostasis.^3–5^ A recent study utilizing advanced technologies determined a more precise half-life of approximately 19 h, which is still less than 1 day.^6^ However, neutrophils within the tumor have a relatively prolonged lifespan, with evidence showing that they can persist for more than 5 days.^7^ Neutrophils are traditionally considered short-acting effector cells in the innate immune system and the primary line of defense against extracellular pathogens and acute inflammation.^4,8–11^ Their short lifespan contributes to the efficiency of the immune system,^12^ and a substantial decrease in neutrophils in the bloodstream results in severe immunodeficiency in humans.^13,14^ However, neutrophils are more complex than initially believed, exhibiting significant functional and phenotypic heterogeneity, particularly in pathological contexts such as inflammation and cancer, contributing to the development of various diseases.^15,16^ They are integral to the regulation of immune responses, mediated through a diverse array of cell surface receptors.^17^ Toll-like receptors recognize microbial structures to prevent pathogen invasion,^18^ while Fcγ receptors and C-type lectins are pivotal in activating the adaptive immune response.^19^ Other receptors, such as G protein-coupled receptors —also known as 7-transmembrane receptors—, receptor tyrosine kinases, and adhesion molecules such as selectins/selectin ligands and integrins, are involved in diverse functional activities of neutrophils.^20–22^ Following receptor-ligand binding, multiple downstream signal transduction pathways are activated, which regulate transcription factors through phosphorylation cascades, mediating numerous neutrophil functions.^23^ Therefore, understanding the regulatory relationship between neutrophil receptors and their downstream signaling pathways contributes to elucidating the diversity and functions of neutrophils in both health and disease.

This review begins by detailing the history of neutrophil research and their physiological functions. We then elaborate on the biological functions of key receptors on neutrophils and the regulation of neutrophil functions through their downstream signal transduction pathways. Furthermore, we present a comprehensive perspective on neutrophil involvement in the pathogenesis of various diseases and explore current therapeutic strategies targeting neutrophils, enhancing our understanding of disease development and informing novel therapeutic approaches.

## Neutrophil discovery and development

**Fig. 1:**
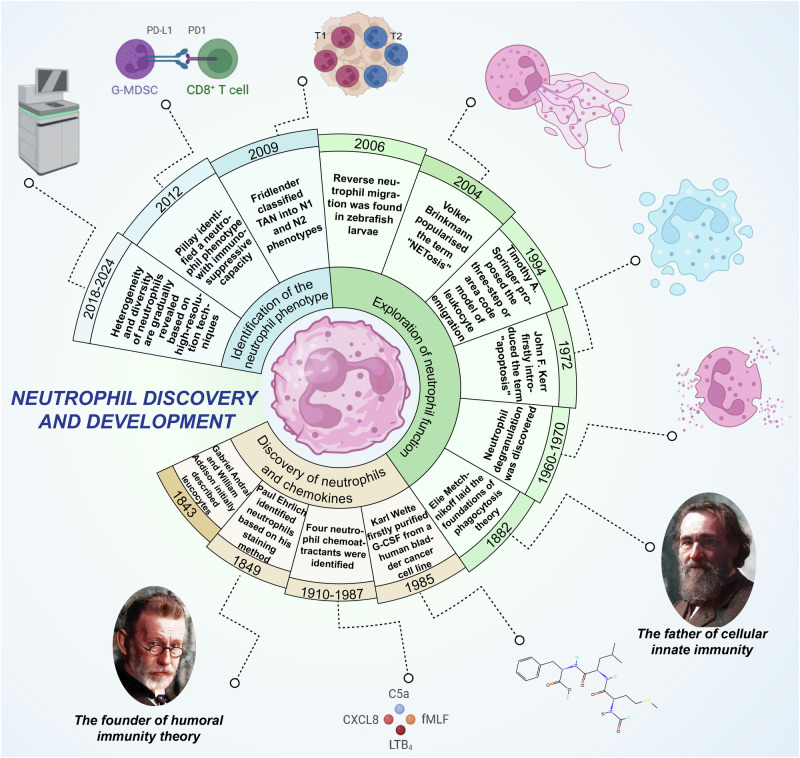
Review of neutrophil discovery and development. Neutrophil development has undergone three phases: the discovery of neutrophils, the exploration of neutrophil function, and the identification of the neutrophil phenotype. LTB_4_ leukotriene B_4_, CXCL8 C-X-C chemokine ligand 8, fMLF N-formyl-methionyl-leucyl-phenylalanine, C5 The fifth complement component, TAN Tumor-associated neutrophil, PD-L1 Programmed cell death ligand 1, PD-1 Programmed cell death protein 1, G-MDSC Granulocytic Myeloid-Derived Suppressor Cell, G-CSF Granulocyte colony-stimulating factor. Figure created with BioRender.com

Developments in research on neutrophils reflect developments in science and technology. Thanks to the invention of the microscope and advances in cell separation techniques, the structure and function of neutrophils were gradually discovered. Subsequent developments in molecular biology and genetic engineering have provided deeper insights into the molecular mechanisms and signaling pathways of neutrophils. More recently, the emergence of single-cell and gene-editing technologies has enabled more in-depth studies of the properties and functions of individual neutrophils. The journey of neutrophil research is the culmination of the tireless efforts of many pioneers, shown in Fig. 1. This 200-year journey has profoundly deepened our understanding of inflammatory immunity and tumor immunity, significantly contributing to advancements in medicine.

### Discovery and identification

In ancient civilizations, humans understanding of blood was restricted to symbolic interpretations and religious convictions. The analysis of blood composition became possible only after the invention of the compound microscope.^24^ Since then, the composition, function, and role of blood have been explored in depth. Leukocytes were initially described in 1843 by Gabriel Andrai and William Addison.^25^ However, no suitable methods to identify these leukocytes were available. The eosinophil was discovered by Jones in 1846.^26^ By 1879, Paul Ehrlich had introduced techniques for blood film staining with coal tar dyes. Based on these approaches, he identified eosinophils, neutrophils, basophils, and lymphocytes.^27^ This breakthrough in staining methodologies marked a pivotal advancement, laying the foundation for modern leukocyte research.^28^

### Discovery of chemoattractants

The term “chemokine” is specifically used to describe those cytokine subpopulations that enable the migration of immune cells from one immune organ or site to another. Chemoattractants, derived from chemokines, are a class of biomolecules that can regulate the assembly, depolymerization, and contractility of cytoskeletal proteins, as well as the expression of protein adhesion molecules on the cell surface.^29^ Neutrophil chemoattractants in humans can be classified into four biochemically distinct subfamilies: formyl peptides, chemokines, complement anaphylatoxins, and chemotactic lipids. The major molecules from each subfamily, which have the most significant impact on neutrophil chemotaxis, are as follows: N-formyl-methionyl-leucyl-phenylalanine (fMet-Leu-Phe or fMLF), C-X-C chemokines, C5a, and leukotriene B_4_ (LTB_4_).^30^ These chemoattractants activate downstream signal transduction pathways by binding to their corresponding receptors and inducing concentration-gradient-dependent neutrophil migration.^31–33^

### Discovery of granulocyte colony-stimulating factor

Granulocyte colony-stimulating factor (G-CSF) is a cytokine synthesized by both non-hematopoietic and hematopoietic cells, exerting a pivotal influence on the regulation of granulocyte proliferation, differentiation, and mobilization from the bone marrow.^34^ Human G-CSF was initially purified by Karl Welte from a human bladder cancer cell line.^35,36^ Advancements in molecular biology techniques led to the swift cloning of the G-CSF gene, facilitating large-scale production of this cytokine and paving the way for its widespread clinical application.^37,38^ G-CSF plays a critical role in neutrophil production in healthy and diseased states and can dramatically increase neutrophil counts during emergencies, such as infections and circumstances that compromise bone marrow function, including cytotoxic chemotherapy for cancer.^39^ The first clinical trial of G-CSF was designed to treat chemotherapy-induced infections and neutropenia.^40,41^ Subsequently, more established G-CSF analogs were gradually developed and received approval from the Food and Drug Administration (FDA).

### Neutrophil recruitment cascade model

To fight infection and clear pathogens, inflammatory responses involve the recruitment of multiple cells to the site of action, with neutrophils needing to be precisely targeted to the inflammation site.^42^ In most tissues, the leukocyte recruitment follows a well-characterized cascade of sequential steps, including tethering, rolling, firm adhesion, intravascular crawling, and transendothelial migration.^43^ Among numerous proposals, a three-step paradigm of leukocyte emigration from the bloodstream proposed by Timothy A. Springer in 1994 seemed to explain the mechanism of neutrophil recruitment well. The model focuses on the fact that the migration of neutrophils and monocytes from the vascular system is regulated by at least three different molecular signals: selectins, chemoattractants, and integrins. The model’s crucial feature is that selectin-carbohydrate, chemoattractant-receptor, and integrin-immunoglobulin interactions act sequentially rather than in parallel.^44^ Selectin, a type I transmembrane cell adhesion molecule, mediates cell-cell interactions by specifically recognizing and binding to carbohydrate structures on the surfaces of other cells.^45^ Selectins can be divided into three families according to the cell type in which they were first characterized: the L-, E-, and P-selectins, of which the L-selectins are expressed in neutrophils. Integrins, a family of ubiquitously expressed transmembrane receptors, anchor cells within their ambient extracellular matrix (ECM) and bind to counter-receptors expressed on other cells.^46^ Multiple molecular choices at each step of the neutrophil recruitment cascade provide a huge combinatorial diversity of signals. The three signaling molecules are closely linked and interact with each other to ensure the tight regulation of neutrophil recruitment. However, the cascade model of neutrophil recruitment has recently been revised and questioned, leading to a shift in the prevailing paradigm. Specifically, the previous broad recruitment hypothesis does not appear to apply to all activation steps within all organs.^47–49^ Nevertheless, more research is needed to prove the hypothesis, and perhaps, in the near future, our understanding of the classical model will be expanded or even subverted.

### Neutrophil phagocytosis

Neutrophils are pivotal innate immune cells, particularly during the early stages of infection, owing to their exceptional ability to eradicate pathogens. Phagocytosis, the process by which neutrophils detect and engulf particles into specialized organelles known as phagosomes, is essential for their pathogen-eliminating function.^50^ The concept of phagocytosis as a fundamental host defense mechanism was first introduced by Elie Metchnikoff.^27^ He laid the foundations of phagocytosis theory in 1882 when he observed protective cells around inserted spines in starfish larvae. In the 1960s, neutrophils were shown to undergo granule fusion with phagocytic vesicles following the engulfment of bacteria, later known as degranulation, following which the contents of the granules were decreased, indicating that neutrophils have a pathogen-fighting function.^51^ The neutrophil phagosome is a specialized organelle, originating from the invagination of the plasma membrane to fully encapsulate the engulfed particle. The formation of a phagosome activates a series of signals, subsequently transforming the originally inert environment of phagocytosis into one optimized for the degradation of ingested particles.^52,53^ The complex mechanisms controlling the process of neutrophil phagocytosis are controversial. Phagosome maturation progresses through an endocytic maturation pathway in most phagocytes, such as macrophages, during which the phagosome merges with lysosomes to form a phagolysosome.^54^ However, in neutrophils, the presence of pre-formed granules in the cytoplasm allows the phagolysosome to remove pathogens faster. Despite this discrepancy, increasing studies have affirmed the existence of phagosomes and attempted to explore the potential underlying mechanisms.^50,53,55^

### Neutrophil death

Under homeostatic conditions, the body produces a large number of neutrophils; the number is even higher in inflammatory conditions. Mature neutrophils do not undergo cell division and have a relatively short lifespan.^56^ This dynamic balance ensures that neutrophils function as an immune defense without causing additional damage to the organism. Like epithelial cells and fibroblasts, neutrophils undergo various death modalities, including apoptosis, pyroptosis, ferroptosis, and NETosis (which is unique to neutrophils).^57^ The term “apoptosis” was first introduced by John F. Kerr in 1972 to describe the observed process of cell loss under physiological and pathological conditions.^58^ Cookson and Brennan added the concept of pyroptosis in 2001 to describe caspase-1-dependent cell death in inflammatory cells.^59^ The term “ferroptosis,” proposed by Brent R. Stockwell in 2012, describes a distinctive form of cell death characterized by iron-dependent phospholipid peroxidation, underscoring the pivotal role of glutathione peroxidase 4 (GPX4) in regulating this process.^60^ Neutrophils can also enhance the defenses against acute infections of the innate immune system by releasing a fibrous extracellular structure called the neutrophil extracellular trap (NET) that accompanies neutrophil death.^61^ Volker Brinkmann popularized the term “NETosis” in 2004 to describe the phenomenon of neutrophils fighting pathogens by “committing suicide“.^62^ Death may not be the only outcome for neutrophils in sterile inflammation. Initially, researchers observed that neutrophils could re-enter the vascular system in a unique process called reverse transendothelial migration (rTEM).^63^ Subsequently, a study in 2006 showed that neutrophils within tissues reversely migrated away from the site of injury in zebrafish larvae,^64^ which may be another fate for neutrophils at sites of sterile inflammation.

### Tumor-associated neutrophils

Beyond their well-established function as innate immune effector cells defending against pathogens, neutrophils have increasingly been recognized as a key constituent of immune infiltrate within tumor microenvironments, exhibiting significant heterogeneity in the context of cancer.^65^ Neutrophils within tumors also exhibit a prolonged lifespan, persisting for more than 5 days.^7^ Tumor-associated neutrophils (TANs) are neutrophils present in tumors, typically displaying tumor-promoting properties.^66^ Studies on neutrophil functions in tumors have a long history, with origins that are difficult to trace. Fridlender et al. demonstrated in 2009 that transforming growth factor-β (TGF-β) is responsible for the polarization of TAN phenotypes, classifying them into N1 and N2 types.^67^ N1 neutrophils display an anti-tumor profile, whereas N2 neutrophils exhibit a pro-tumor phenotype.^67^ TGF-β induces N2 neutrophil polarization, whereas type I interferon (IFN I) induces N1 polarization.^67,68^ Additionally, polymorphonuclear myeloid-derived suppressor cells (PMN-MDSCs), distinguished by pro-tumorigenic and immunosuppressive activities, have been identified in tumor tissues.^69^ Lectin-type oxidized low-density lipoprotein (LDL) receptor 1 (LOX1) is markedly overexpressed in PMN-MDSCs,^70^ suggesting PMN-MDSCs as a functionally distinct immunosuppressive subset of TANs. Neutrophils in the tumor microenvironment can also be classified according to their density gradient, with mature (segmented) high-density neutrophils (HDN) characterized by anti-tumor activities, and immature (banded) low-density neutrophils (LDN), including PMN-MDSCs, displaying immunosuppressive functions observed in cancer patient circulation.^71^ However, recent advancements in high-resolution cell sequencing technologies are likely to refine these classifications. A study employing single-cell RNA sequencing of neutrophils across various tumors and organs identified three distinct neutrophil states within the tumor microenvironment: T1, T2, and T3.^7^ This study suggests that neutrophils undergo aggregate reprogramming within tumors, which may better explain their plasticity. In summary, neutrophils display significant plasticity and functional diversity within the tumor microenvironment, making them a current focal point in tumor immunotherapy research.

### Immunosuppressive neutrophils

Traditionally, neutrophils are considered innate immune effector cells that initiate an adaptive immune response by releasing multiple inflammatory mediators.^72^ However, recent studies have shown that neutrophils also exhibit immunosuppressive capacities.^73–75^ While suppression of the immune response is necessary to limit tissue damage during inflammation, in certain diseases, such as sepsis and cancer, it can lead to a breakdown of the immune defense system.^65,76^ In 2012, Pillay et al. identified a unique human neutrophil phenotype with immunosuppressive capabilities during acute inflammation,^77^ establishing the foundation for subsequent extensive research on immunosuppressive neutrophils. One study revealed that neutrophils from patients with septic shock exhibit increased oxidative burst and phagocytosis, while their chemotaxis is strongly inhibited.^78^ A subsequent single-cell sequencing study demonstrated that these immunosuppressive neutrophils exhibit high PD-L1 expression and attenuate the innate immune response by promoting an increased proportion of Tregs.^76^ Neutrophils can also inhibit T cell function, facilitating immune evasion by tumor cells.^65^ Immunosuppressive neutrophils also mitigate tissue damage caused by excessive inflammatory responses.^73^ Compared to bone marrow neutrophils, lung neutrophils exhibit immunosuppressive characteristics due to alterations in multiple genes and surface markers. This phenotype protects the lungs from damage resulting from excessive infection, injury, and dysfunctional/homeostatic responses.^79^ In summary, immunosuppressive neutrophils function as a double-edged sword, with their harmful influence on disease progression demanding thorough evaluation and strategic consideration.

### Neutrophil examination under high-resolution technology

Historically, neutrophil developmental stages were identified through Giemsa staining-based histologic examination.^80^ However, recent advancements in multi-omics technologies, such as single-cell sequencing and transcriptome sequencing, have significantly improved our understanding of neutrophil development and functional activities.^81^ A previous study utilizing mass spectrometry cytometry and flow cytometry identified three neutrophil subpopulations in murine bone marrow: committed proliferative neutrophil precursors (preNeu), non-proliferating immature neutrophils, and mature neutrophils. Transcriptomic and functional analyses revealed that preNeu requires the C/EBPε transcription factor for differentiation from granulocyte-monocyte progenitors (GMPs).^82^ A subsequent study using transcriptomic and proteomic analyses identified that proNeu1, marked by Lin^–^CD117^+^CD34^hi^Ly6C^+^CD115^–^CD81^+^CD106^–^CD11b^lo^ and present in GMP, initially generates the intermediate progeny proNeu2, marked by CD117^+^CD34^lo^Ly6C^+^CD115^–^CD81^+^CD106^+^CD11b^hi^, and then differentiates into Lin^–^Siglec-F^–^CD117^+^CXCR4^+^Gr1^+^CD11b^+^ preNeu.^83^ Further, recent research identified the CD66b^–^CD64^dim^CD115^–^ cell population in human bone marrow as neutrophil-directed progenitor cells. This study also indicated that neutrophils at various developmental stages, distinguished by their cellular morphology, display unique transcriptomic profiles, suggesting that combining morphological characteristics with transcriptomic analysis offers a precise approach for identifying distinct stages of neutrophil differentiation.^84^

A study integrating transcriptional and chromatin analyses of neutrophils during acute inflammation identified two important transition points in neutrophil transcriptional regulation: the shift from bone marrow to blood and the subsequent transition from blood to tissue. This research elucidated the function of binding motifs for several transcription factors, such as RUNX1 and KLF6, which regulate neutrophil maturation; RELB, IRF5, and JUNB, which drive neutrophil effector responses; and RFX2 and RELB, which promote neutrophil survival.^85^ These findings pave the way for the development of specific therapies based on the distinct functional phases of neutrophils. High-resolution cytometric sequencing has also been applied to analyze neutrophils in tumor tissues. A study employing single-cell RNA sequencing of neutrophils in various tumors and organs identified three distinct neutrophil states in the tumor microenvironment: T1, T2, and T3.^7^ This study introduced the concept of neutrophils undergoing aggregate reprogramming within the tumor, providing a better explanation for neutrophil plasticity in tumors. Collectively, high-resolution technologies, including mass spectrometry, single-cell cytomics, high-throughput sequencing, and flow cytometry, refine the understanding of neutrophils from a single cell population to diverse subpopulations, enhancing the exploration of neutrophil heterogeneity and diversity.

## Functional activities of neutrophils

Neutrophil homeostasis is governed by a delicate equilibrium of cell maturation, release from the bone marrow, migration through vascular and tissue networks, aging, and programmed cell death.^6^ Upon encountering a foreign pathogen, neutrophils rapidly detect the signal and move to the infected site. They then engage in effective pathogen elimination through phagocytosis for immune defense.^53^ Unactivated neutrophils undergo various forms of cell death to ensure prompt clearance from the body, maintaining overall homeostasis through regulated production and rapid turnover. However, neutrophils exhibit a notably prolonged lifespan within tumor tissue and are involved in the tumor immune response through complex mechanisms.^86^ Understanding the potential functions of neutrophils in these contexts is key to designing effective clinical therapies. Here, to explore their potential functions and inform therapeutic strategies, the processes governing neutrophil production and cell death within the organism, as well as their involvement in the tumor immune response will be elucidated.

### Granulopoiesis and release

**Fig. 2:**
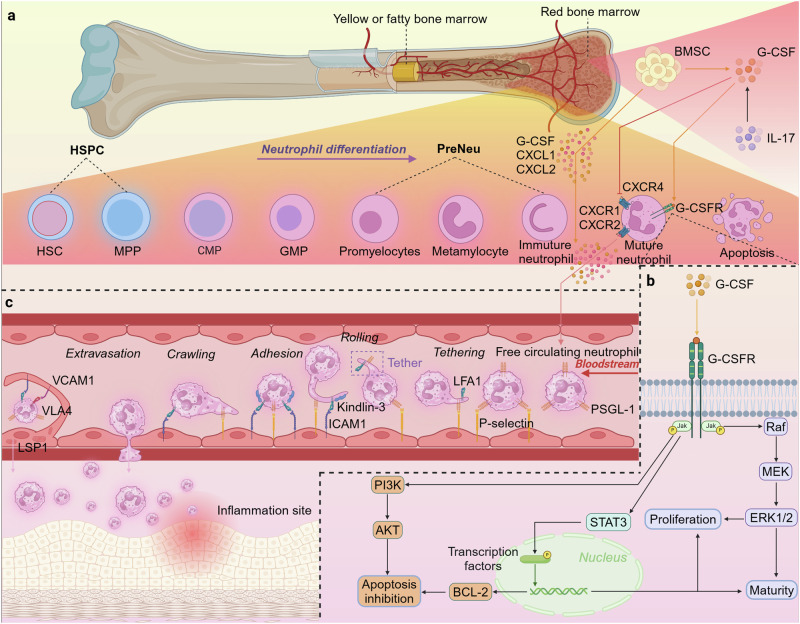
Schematic diagram illustrating the process of granulopoiesis in the bone marrow and the neutrophil recruitment cascade model. **a** Neutrophil production begins with HSCs in the bone marrow. The HSCs undergo a series of differentiation steps to enter the promyelocytic stage. The promyelocytes subsequently continue to differentiate and are ultimately released into the peripheral blood in the form of mature neutrophils. **b** G-CSF promotes and regulates the proliferation, differentiation, and release of neutrophils from the bone marrow. **c** The neutrophil recruitment cascade involves the following steps: tethering, rolling, adhesion, crawling, and finally, migration. Selectins, integrins, and chemokines regulate each step to ensure neutrophil chemotaxis. HSC Hematopoietic stem cell, MPP Multipotent progenitor cell, CMP Common myeloid progenitor cell, GMP granulocyte-monocyte progenitor cell, preNeu Neutrophil precursors, G-CSF Granulocyte colony stimulating factor, PSGL1 P-selectin glycoprotein ligand 1, BMSC Bone marrow stromal cell, LFA-1 Leukocyte function antigen 1, ICAM1 Intercellular adhesion molecule 1, VCAM1 Vascular cell adhesion molecule 1, LSP1 Lymphocyte-specific protein 1. Figure created with BioRender.com

The lifecycle of neutrophil begins with granulopoiesis in the bone marrow.^87^ Hematopoietic stem cells (HSCs) are located at the apex of the hematopoietic hierarchy and exhibit a distinct self-renewal and differentiation capacity. Upon activation, HSCs differentiate into multipotent progenitors (MPPs). Together, HSCs and MPPs comprise the pool of hematopoietic stem and progenitor cells (HSPCs).^88,89^ Subsequently, MPPs continue to differentiate into common lymphoid progenitor cells (CLPs) or common myeloid progenitor cells (CMPs). CLPs and CMPs have proliferative and differentiating capacities, respectively. CLPs differentiate into lymphocytes and natural killer cells. CMPs differentiate into the bone marrow lineage, of which granulocyte-monocyte progenitors (GMPs) can eventually differentiate into neutrophils.^90^ (Fig. 2a).

In the classical model of granulopoiesis, neutrophil-committed progenitors called promyelocytes and myelocytes are located downstream of GMPs.^91^ These immature proliferating progenitors are now referred to as neutrophil progenitors (NePs) and neutrophil precursors (preNeus). The differentiation of preNeus to mature neutrophils is characterized by a change in the shape of the nucleus: promyelocytes (round nuclei) - metamyelocytes (kidney-shaped nuclei) - immature neutrophils (banded nuclei) - mature neutrophils (classical segmented nuclei).^90^ Under homeostatic conditions, only mature neutrophils are released into the peripheral blood. However, during infection, inflammation, or cancer, the transport of neutrophils into the bloodstream is increased, and non-proliferating immature neutrophils can also be released into the periphery.^82,92^

To exit the bone marrow, mature neutrophils must traverse the sinusoidal endothelium separating the hematopoietic zone from the bloodstream.^87^ Thus, neutrophils appear to pass through the bone marrow endothelium *via* the transcellular rather than the paracellular pathway.^93^ Endothelial penetrability is significant in this process. Vascular endothelial calcineurin regulates and decisively influences endothelial cell homeostasis and the resulting release of cells from the bone marrow.^94^ Neutrophil release is facilitated by gradients of mediators across the bone marrow sinus wall, including CXCL1, CXCL2, and G-CSF.^93,95^ G-CSF is secreted by stromal cells within the bone marrow and mediates the generation of homeostatic neutrophils. IL-17 has been implicated as a pivotal regulator of G-CSF expression during emergency granulopoiesis and basal neutrophilia.^96^ IL-17 was also found to be critical to G-CSF-mediated neutrophil responses during infection, as mice lacking IL-17A receptors exhibited increased susceptibility to *Klebsiella pneumoniae* due to decreased production of G-CSF at the site of infection.^97^

G-CSF combines with a single homologous receptor (G-CSFR; CSFR3) expressed on the surface of target cells to activate an intracellular signaling cascade that is crucial to granulocyte proliferation, differentiation, release, transport, and survival.^98^ The interaction between G-CSF and its receptor induces conformational changes within the receptor’s cytoplasmic domains, subsequently triggering the activation of multiple downstream signaling pathways (Fig. 2b). G-CSF receptor-associated Janus kinase 1/2 (JAK1/2) rapidly activate the mitogen-activated protein kinase (MAPK) and phosphoinositide 3-kinase (PI3K) signaling pathways^39,99^ and phosphorylate signal transducer and activator of transcription family member 3 (STAT3), causing them to translocate into the nucleus to facilitate the transcription of target genes.^100,101^ G-CSF accelerates the transformation of progenitor cells into mature neutrophils, mediated by the MAPK and STAT3 signaling pathways.^102,103^ Additionally, G-CSF upregulates the expression of cell survival proteins, including B-cell lymphoma 2 (Bcl-2) family members, which synergize with the PI3K signaling pathway and inhibit neutrophil apoptosis.^104^ Bone marrow mesenchymal stem cells (MSCs) may preserve neutrophils from apoptosis, prevent over-activation of oxidative metabolism, and maintain their effector functions.^105^ G-CSF also antagonizes CXCL12/CXCR4 expression based on a different orientation toward neutrophil migration. G-CSF downregulates CXCL12 expression, promotes CXCL12 cleavage by proteases, and reduces CXCR4 expression on the neutrophil surface.^106^ CXCR2 is another chemokine receptor that is expressed in bone marrow cells. CXCL1 and CXCL2, its ligands, are produced by bone marrow endothelial cells and assist G-CSF in neutrophil mobilization.^107^ In conclusion, the bone marrow serves as a reservoir of mature neutrophils, readily deployable into peripheral blood in response to infection.

### Recruitment and migration

Neutrophil recruitment is initiated by alterations in the endothelial surface, which are stimulated by inflammatory mediators released by tissue-resident sentinel leukocytes on contact with pathogens.^9,108^ Pathogen detection mediated by pattern recognition receptors (PRR) directly activates endothelial cells, upregulating the expression of adhesion molecules.^9^ PRRs, which are innate immune receptors, rapidly recognize different pathogenic invaders to initiate and amplify the innate immune response.^109^ During host-pathogen interactions, PRRs can detect microbial conservative structures from microbes, namely PAMPs, and recognize molecular patterns expressed on injured host cells, namely damage-associated molecular patterns (DAMPs).^110^

Neutrophil recruitment occurs predominantly in postcapillary microvessels in the microcirculation, but has also been observed in the arterial system under pathological inflammatory conditions.^111^ Neutrophils are free-flowing cells within the bloodstream. The binding of L-selectin to endothelium-derived ligands triggers the bundling and rolling behavior of neutrophils, constituting the first step in a multi-step adhesion cascade.^45^ In fact, the preexisting P-selectin in Weibel-Palade bodies is the fastest expressed selectin. P-selectin tethers (captures) free-flowing neutrophils to the surface of endothelial cells by binding to P-selectin glycoprotein ligand 1 (PSGL1), subsequently rolling along blood vessels in the direction of blood flow^112^ (Fig. 2c). Neutrophils can also interact directly with endothelial cells to achieve capture.^113^ Subsequently, they undergo a continuous slow interaction called “rolling.” The fast rolling of neutrophils mainly depends on endothelial (E)- and/or P-selectin. E-selectin that express relatively slowly mediates the slow rolling of neutrophils and activates integrin expression.^114–116^ PSGL-1 is anchored to the actin cytoskeleton and facilitates the dynamic actin polymerization necessary for the slow rolling of neutrophils.^117^ Neutrophils rolling at high shear rates of blood require rapid adhesive formation and disruption, and the dissociation of the P-selectin-PSGL1 bond at the trailing edge of the cell must be counterbalanced by the formation of a different bond at the leading edge.^118,119^ Neutrophils were observed to form small membrane structures called “tethers” at the back and “slings” at the front that increased the membrane’s bonding area through total internal reflection fluorescence microscopy. Membrane slings are characterized by regions of high PSGL-1 expression and the presence of leukocyte function antigen 1 (LFA-1), serving as an adhesive matrix that facilitates neutrophil rolling even under high shear rates.^120^ Initially, the tether forms a temporary anchorage site through PSGL1 binding to P-selectin. As the cell rotates forwards, the tether tears posteriorly in neutrophil rolling units and PSGL1 is progressively stripped from its ligand by the breakage of these tethers. Subsequently, the tether swings to the front to act as a sling. The neutrophil rolls in this manner until it reaches the adherence zone.

The rolling and slow deceleration of neutrophils facilitate the binding of chemokine receptors on the membrane to ligands released by endothelial cells to induce integrin activation.^43^ Integrins are generally accepted to adopt at least three distinct conformations: a low-affinity bent conformation, an extended closed conformation with intermediate affinity, and an extended open conformation characterized by high ligand affinity.^121,122^ These conformational changes can be induced and stabilized by intracellular signals (activated from the inside out) or triggered by tightly regulated extracellular ligands (activated from the outside in), such as fibronectin.^22^ Activated intracellular signaling ultimately triggers a change in integrin affinity for its ligand, also known as integrin inside-out activation, which ultimately leads to the combination of kindlin and talin to the β_2_-integrin’s tail. Kindlin and talin are two cell adhesion-associated proteins that activate integrins and act as linkers in the adhesion process. These two proteins initiate the assembly of adhesion complexes to anchor integrins to the actin cytoskeleton and establish signaling hubs that orchestrate various cellular processes (integrin outside-in signaling).^123,124^ Neutrophils consistently express high levels of the integrins MAC1 and LFA-1, both of which undergo conformational changes to combine with common endothelial cell surface molecules, such as intercellular adhesion molecule 1 (ICAM1) and intercellular adhesion molecule 2 (ICAM2).^125^ This will prevent the neutrophils from rolling slowly, leading to their accumulation and stagnation on the endothelial surface.

Following adhesion, neutrophils engage in a slower movement along the endothelium, known as “crawling,” which can occur either with or against the direction of blood flow.^126^ Some adherent neutrophils exhibit behaviors termed “pirouetting” or “probing,” where they elongate and extend pseudopods, seemingly actively scanning and probing their surroundings while remaining anchored at a single location within the microvascular system. Neutrophils normally migrate at the endothelial cell-cell junction and actively crawl to this junction.^43^ Crawling depends on subtle integrin activation-inactivation steps, integrin activation pathway modulators, and actin flow dynamics.^127,128^ On the one hand, endothelial cell-expressed ICAM1 interacts with integrin-expressed MAC1 to regulate active neutrophil crawling.^125^ On the other hand, actin-binding proteins, such as mammalian actin-binding protein 1 (MABP1), enhance the high-affinity conformation of β_2_-integrins, increasing their interactions with actin, which are fundamental for neutrophil crawling under shear stress.^129^ Moreover, several neutrophil-perceivable mechanotropic components, including VAV1 and Cdc42, are involved in neutrophil crawling under shear forces.^130^ These features suggest that neutrophil rolling and adhesion provide an initial localization that allows them to come into close contact with the vascular endothelium and recognize inflammatory signals, whereas crawling allows them to move further along the vessel wall to precise extravasation sites.

Neutrophils are required to pass through several barriers consisting of vascular endothelial cells, basement membranes, and pericytes to get to the site of infection or inflammation.^43^ Paracellular diapedesis represents the predominant pathway for leukocyte extravasation from the vasculature. During the transcellular migration, endothelial cells generate migratory cups—microvillus-like projections that rise along the sides of the neutrophil, eventually extending in vivo to envelop the neutrophil within a dome-like structure. The projections have been shown to enrich vascular cell adhesion molecule 1 (VCAM1) and ICAM1 and form cup-shaped structures around clusters of adherent leukocytes.^131,132^ The dome formation depends on lymphocyte-specific protein 1 (LSP1), an F-actin–binding protein that is expressed in the nucleus and cytoplasm of resting endothelial cells and attaches to the cytoskeleton during endothelial activation.^133^ Subsequently, neutrophils may cross the continuous basement membrane *via* releasing proteases, such as matrix metalloproteinases (MMPs).^134^ Neutrophils engage with pericytes through an ICAM-1-dependent interactions to form extensions that connect with pericyte projections, ultimately seeking out gaps leaving the vascular system.^135^ Pericytes may also reduce contractility through stimulation with a macrophage migration inhibitory factor secreted by endothelial cells, enhancing neutrophil extravasation.^136^ Collectively, these observations suggest that neutrophils tend to favor the path of least resistance during extravasation, actively probing the surrounding macropores for avenues of migration.^137^

Upon breaching the basement membrane and entering the mesenchyme, the neutrophil transitions to an “amoeboid” mode of migration, characterized by cellular deformation, actin polymerization, and the reorganization of surrounding tissue structures.^138^ The intricacies of the neutrophil migration cascade are intricately governed by chemoattractants and their corresponding receptors. These molecules orchestrate concentration-dependent neutrophil migration through downstream signal transduction, a process termed neutrophil chemotaxis. Concentration gradients of chemoattractants determine the orientation of intravascular crawling and direct neutrophils to the site of infection or inflammation.^31,139^ Chemotaxis encompasses three interrelated processes: the periodic extension of typical pseudopods, the establishment of cellular polarity that heightens anterior sensitivity relative to the posterior, and orientation sensing, which continuously aligns the chemotaxis with the external gradient.^140,141^ Microfilament, also known as actin filament, is one of the main elements of the cytoskeleton, supporting the shape of the cell and maintaining the cellular architecture.^142^ Pseudopod formation is largely dependent on the dynamic reorganization of microfilaments. Nucleation serves as the rate-limiting step in actin assembly, which is finely regulated by the actin-related protein 2/3 (Arp2/3) complex.^143^ In the absence of chemotactic stimuli, microfilaments in the cytoskeleton are extended into a dynamic pseudopod that lasts about 60 s in an almost random direction. The pseudopod subsequently either retracts or anchors to the underlying substrate, facilitating the forward propulsion of the cell body. Concentration gradients formed by chemoattractants in tissues increase the likelihood of pseudopod formation on the side of their most intense concentration and suppress the establishment of lateral pseudopods. This is directional sensing.^141^ Moreover, chemoattractant concentration gradients cause neutrophils to form a specific structure that has anterior and posterior asymmetry. This is neutrophil polarization.^144^ Neutrophil polarization is regulated by intracellular signaling mediated by the combination of chemoattractant with G protein-coupled chemoattractant receptor.^145^

In conclusion, the migration and polarization of neutrophils involve a drastic reconfiguration of the tubulin cytoskeleton, which involves actin polymerization. Actin polymerization is regulated by many regulators and protein families activated by signal transduction. Myosin contraction also involves the migration and polarization of neutrophils. Myosin-II filaments, which are predominantly found in the dorsal and lateral cortexes of cells, provide the power to retract the uropod and suppress the formation of lateral pseudopodia.^146^ Actin polymerization and pseudopod formation at the leading edge of the cell and impetus for retraction provided by myosin at the trailing edge ensure neutrophil migration along the chemotactic gradient.

### Phagocytosis

**Fig. 3:**
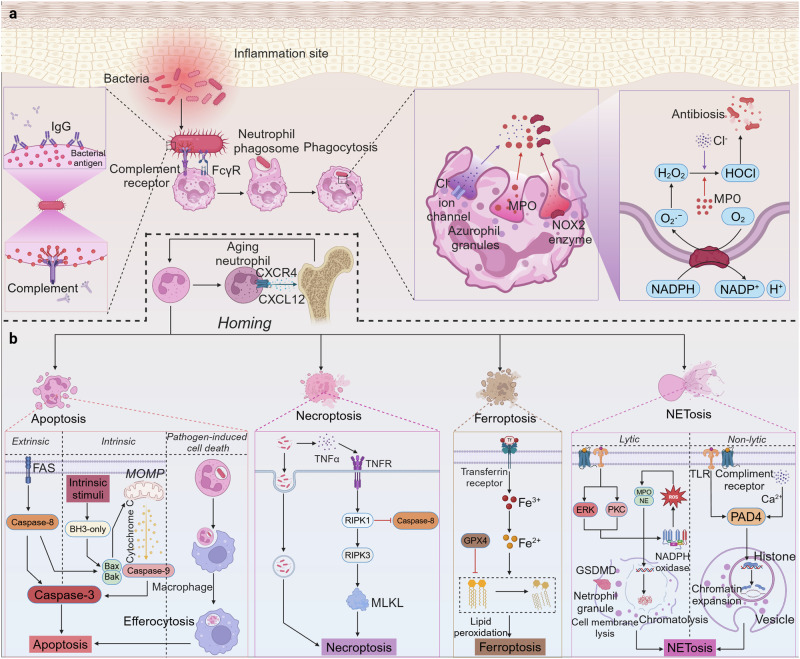
Schematic diagram illustrating phagocytosis and the ultimate fate of neutrophils. **a** Initially, neutrophils recognize antigenic proteins encapsulated on pathogens, such as IgG and complement complexes, via receptors on the cell membrane. Subsequently, neutrophils capture the pathogen by forming phagosomes. The fusion of neutrophil azurophilic granules with phagosomes inserts antimicrobial granule proteins, such as MPO, NE, and lysozyme, as well as membrane proteins, including receptors, ion channels, and NOX2 enzymes, into the phagosomal membrane. H_2_O_2_ produced by NADPH oxidase interacts with Cl^-^, which is shuttled to the phagosome through ion channels, to generate HOCl with strong oxidative antimicrobial effects in the presence of MPO. Neutrophils eliminate pathogens via both of the above cytotoxic pathways. **b** Neutrophils need to be removed promptly to prevent an excessive and unintended inflammatory response. On the one hand, senescent neutrophils are recruited back to the bone marrow by the upregulation of CXCR4 expression on the membrane surface and increased sensitivity to CXCL12 expressed in the bone marrow, namely neutrophil homing. On the other hand, neutrophils are eliminated in vivo through multiple death modalities, including apoptosis, necroptosis, ferroptosis, and NETosis. IgG Immunoglobulin G, MPO Myeloperoxidase, MOMP Mitochondrial Outer Membrane Permeabilization, NE Neutrophil elastase, GSDMD Gasdermin D, GPX4 Glutathione peroxidase 4, PAD4 Protein arginine deiminase 4, RIPK Receptor-interacting serine/threonine-protein kinase, MLKL Mixed Lineage Kinase Domain-Like protein. Figure created with BioRender.com

After a series of migratory movements, neutrophils eventually reach the site of infection or inflammation. Initially, soluble molecules within the bloodstream and interstitial fluids detect pathogens, with immunoglobulins recognizing foreign antigens and elements of the complement cascade depositing on external surfaces. These molecules deposited on foreign particles are called opsonins.^147^ Neutrophils recognize opsonins coating the pathogen via receptors on the cell membrane, stimulating phagocytosis (Fig. 3a). Phagocytosis triggers the activation of multiple signaling pathways, leading to the dynamic reorganization of the actin cytoskeleton and the formation of nascent phagosomes. Fcγ receptor (FcγR)- and complement receptor-mediated phagocytosis signaling pathways are mainly involved in phagosome formation. FcγRs engage with immunoglobulin G (IgG)-coated targets, while complement receptors recognize complement-coated particles. Upon receptor activation, the plasma membrane extends pseudopods, forming a cup-shaped enclosure around the target. This process is driven by the rearrangement of the actin cytoskeleton.^147^ Actin polymerizes at the leading edge and along the phagosomal cup, with this polymerization sustained until the phagosome constricts and seals.^148^

Within phagosomes, two pivotal cytotoxic mechanisms are initiated: (1) the generation of reactive oxygen species (ROS) *via* nicotinamide adenine dinucleotide phosphate (NADPH) oxidase and (2) the release of bactericidal proteins from pre­formed granules, namely oxidative and non-oxidative killing.^149^ Subsequently, the phagosome translocates to the center of the cell, during which pre-existing particles within the neutrophil fuse with the phagosome to kill the pathogen. Four types of neutrophil granules have been identified, including specific granules, gelatinase granules, azurophilic, and secretory vesicles, with the azurophilic granules posing the greatest threat to phagocytic bacteria.^150^ Azurophil granules possess potent bactericidal activity, harboring a range of antimicrobial components including bactericidal/permeability-increasing protein, myeloperoxidase (MPO), defensins, cathepsin G, lysozyme, and proteinase 3.^54^ These proteins play multifaceted roles in microbial eradication, including promoting membrane permeabilization and lysis, exemplified by defensins and cathelicidins, and sequestering essential metals critical for microbial growth and enzymatic function, as seen with lactoferrin.^151^ During phagosome formation and maturation, the fusion of particles with the phagosome serves two critical functions: the delivery of antimicrobial particulate proteins into the lumen and the incorporation of functionally essential membrane proteins into the phagosome membrane, including ion channels, receptors, and NADPH oxidase 2 (NOX2) enzymes.^54^ The activation of NADPH oxidase is a dynamic process orchestrated by a cascade of kinase signaling, calcium release, and the activation of small GTPases, culminating in the subsequent activation of NOX2.^152^ Among the numerous ROS generated by NOX2, MPO-catalyzed iodination and chlorination are thought to primarily mediate neutrophil bactericidal mechanisms.^153^ Although the primary mechanism of neutrophil bactericidal activity is controversial, the current conclusion is that there is no single primary mechanism for oxidative and non-oxidative killing, but rather a complex synergistic relationship. When one mechanism is impeded, another mechanism serves as an alternative compensatory system.

### Death

The powerful antimicrobial activity of neutrophils can be a double-edged sword, and their untimely removal may lead to an excessive and unintended inflammatory response in the host body.^154^ Nonlytic neutrophil death pathways, mainly apoptosis, predominate during homeostasis (Fig. 3b). This form of neutrophil death facilitates anti-inflammatory immune responses by sequestering intracellular granules and cytoplasmic components.^155^ Neutrophil apoptosis can be divided into two forms according to the mechanism of occurrence: apoptosis with the activation of classical death signaling pathways and pathogen-induced apoptosis.^156,157^ Pro-apoptotic dimers of the Bcl-2 family in the intrinsic pathway, particularly Bcl-2 homologous antagonist/killer (Bak) and Bcl-2-associated X (Bax), and cell surface death receptors in the extrinsic pathway, specifically FAS, participate in the classical death signaling pathway, orchestrating apoptosis through a caspase-3-dependent manner.^158,159^ Pathogen-induced cell death is a primary formation of neutrophil apoptosis, integrating microbial ingestion and destruction with the expedited clearance of neutrophils *via* specialized phagocytic mechanisms, particularly efferocytosis.^160,161^ Defective neutrophil efferocytosis is associated with the progression of cardiovascular disease in humans, particularly atherosclerosis.^162^ Pathogen-induced cell death also depends on the local production of ROS. In patients with chronic granulomatous disease (CGD), neutrophil deficiencies impair the ability to combat specific fungal and bacterial infections, resulting in prolonged pro-inflammatory responses and consequent tissue damage.^163,164^ However, ineffective clearance of apoptotic neutrophils results in necrosis and the subsequent release of toxic granulocytes and DAMPs into the extracellular space, exacerbating the local inflammatory response and tissue damage. Necroptosis, an ineffective form of apoptotic cell removal, acts independently of normal apoptotic signal transduction. Neutrophil necroptosis can be initiated by various stimuli, including tumor necrosis factor (TNF), TLR, granulocyte-macrophage colony-stimulating factor (GM-CSF), and adhesion receptors, such as CD44 and CD18.^165–167^ The TNF-triggered RIPK1-RIPK3-MLKL signaling pathway is the most widely studied extracellular indicator of necroptosis. Mechanistically, necroptosis begins when the apoptotic protein caspase is inhibited. TNF activates receptor-interacting protein kinase 1 (RIPK1) by binding to the corresponding receptor. RIPK1 inhibits caspase-8 expression, activating downstream receptor-interacting serine-threonine kinase 3 (RIPK3). Necroptosis is predominantly orchestrated by the synergistic activity of RIPK3 and mixed-lineage kinase domain-like (MLKL) proteins.^168,169^ Pathogens are also involved in this form of apoptosis. Community-acquired methicillin-resistant *Staphylococcus aureus* (CA-MRSA) has been observed to persist following neutrophil phagocytosis. CA-MRSA induces neutrophil necroptosis, disrupts macrophage-mediated clearance of neutrophils, and aggravates the infection.^170^ *Chlamydia pneumoniae* and *Klebsiella pneumoniae* were found to impede apoptosis, promoting their survival and replication by hindering neutrophil apoptosis and efferocytosis.^171,172^

Neutrophils can also induce lytic death through certain infectious or inflammatory etiologies. Neutrophil death in this form releases toxic pro-inflammatory chemokines and granule proteins, which may cause or exacerbate local tissue damage if unregulated.^173^ Pyroptosis is a type of lytic cell death that essentially serves to inhibit the infection spread and promote tissue repair in response to foreign pathogen invasion or environmental stresses.^174^ Pyroptosis occurs through the formation of bubble-like protrusions, also known as pyroptotic bodies, which cause cell membrane rupture, releasing the cytoplasmic contents, specifically the pro-inflammatory cytokines IL-1β and IL-18. Beyond their role in fighting pathogens, pro-inflammatory cytokines act as inflammatory signals to attract more inflammatory factors and immune cells to the infected site.^175^ Mechanistically, pyroptosis is governed by the cleavage of gasdermin D (GSDMD) by inflammatory caspases, such as caspase-1, -4, -5, and -11. This process releases the gasdermin-N terminal domain, which possesses inherent pyroptosis-inducing activity.^176^

Ferroptosis represents another form of lytic cell death characterized by the iron-driven accumulation and peroxidation of polyunsaturated fatty acid-containing phospholipids to lethal levels within the cell membrane.^177^ Ferroptosis is intricately regulated by the interplay of iron metabolism, lipid metabolism, and redox pathways, with GPX4 serving as a pivotal mediator in this process. In neutrophils, targeting the redox pathway is critical for regulating ferroptosis.^178^ Recent reports have demonstrated that PMN-MDSCs within the tumor microenvironment exhibited spontaneous ferroptosis-driven death,^179,180^ which may provide a potential therapeutic target for halting tumor progression as a manipulable immunosuppressive mechanism.

NETosis is a particular and regulated form of programmed cell death in neutrophils and a novel mechanism of neutrophil attack on pathogens.^62^ Typically, the intricate process of NET formation is initiated by the recognition of diverse signaling molecules, such as bacteria, bacterial components, and inflammatory factors, triggering the activation of the NET pathway.^181^ Subsequently, the nuclear membrane ruptures, and decompressed nuclear DNA is released into the cytoplasm. This DNA is mixed with cellular components, including granules, histones, and cytoplasmic proteins, ultimately leading to the rupture of the plasma membrane and the subsequent release of the NET structure into the extracellular space. These extracellular traps possess the capacity to inflict damage on virulent pathogens.^182^ Not all instances of NET development result in neutrophil death following the extracellular release of DNA. The term “NETosis” is often employed in current studies to refer to the formation of a NET rather than neutrophil death and can be classified into “lytic” and “non-lytic” forms. The “lytic” mechanism of NET formation is the combination of a ligand with the cellular receptor, which initiates Raf-MEK-ERK signal transduction and mobilizes the NADPH oxidase complex. The complex increases the level of cytoplasmic ROS, which is detected by MPO. Then, MPO triggers a series of reactions to disrupt chromatin packaging and works synergistically with neutrophil elastase (NE) to decondense chromatin. The nucleus, along with its chromatin, undergoes expansion, ultimately leading to cell lysis driven by GSDMD-induced pore formation in the cell membrane.^183,184^ The “non-lytic” mechanism of NET formation involves an increase in cytoplasmic calcium concentration activating protein arginine deiminase 4 (PAD4), which results in the citrullination of arginine residues on histones and reduction of the positive charge. Next, histones gradually lose their electrostatic affinity for DNA, prompting chromatin expansion alongside the nucleus, ultimately culminating in the formation of NETs.^185^ Neutrophils producing NETs in this form do not undergo lysis, and the cells remain viable and perform normal functions, including chemotaxis, phagocytosis, and pathogen killing, after the release of the NETs.^186^

Beyond their primary role in trapping pathogens to prevent infection dissemination, NETs has been implicated in a range of pathological conditions when formed in excess due to dysregulation and impaired elimination mechanisms; these effects include prolonged wound healing, systemic inflammatory responses, impaired immune surveillance, microcrystal disease, thromboembolism, and the facilitation of cancer metastasis.^187–190^ Regarding the diabetic foot, for example, diabetes is found to significantly enhance NET formation within wounds, leading to delayed diabetic wound healing.^191^ In diabetic wounds, excessive NET formation triggers activation of the NOD-like receptor protein 3 (NLRP3) inflammasome and induces the release of IL-1β in macrophages *via* the TLR-4/TLR-9/ nuclear factor κ enhancer of activated B cells (NF-κB) signaling pathway. This cascade perpetuates the local inflammatory response and hinders the wound healing process.^192^ Excessive NETs have also been implicated in delaying diabetic wound healing by promoting endothelial-to-mesenchymal transition through the Hippo pathway.^193^ Thus, an understanding of the pathophysiology of NETosis contributes to clinical practice, especially by surgeons, as NET formation is intricately linked to postoperative complications and cancer recurrence following surgery.^190^ In summary, timely clearance of neutrophils through various forms of cell death in circulation is essential for maintaining the homeostasis of the organism. These forms of neutrophil death are mediated by distinct regulatory mechanisms. However, recent studies have shown that neutrophil apoptosis and NETosis are not mutually exclusive processes. Specifically, if apoptotic neutrophils are not promptly cleared, the opening of GSDME pores can activate PAD4, leading to NETosis as a secondary death process.^194^ These findings underscore the complex interplay in neutrophil death regulation, necessitating further investigation to fully elucidate these mechanisms.

In addition to various forms of death, neutrophils can also undergo reverse migration. Currently, neutrophil reverse migration has been observed across a range of models, such as zebrafish,^195^ mice,^196^ and in vitro human neutrophils.^197^ Several mechanisms have been proposed to elucidate the reverse migration of neutrophils from inflamed tissue. Reduced junctional adhesion molecule-C (JAM-C) expression in epithelial cells (EC) might cause neutrophils to exhibit significant reverse migration. JAM-C can maintain the EC in a polarized manner, creating a unidirectional gate for neutrophil movement from the vascular lumen to the proximal tissue compartment, whereas the effects of NE and certain acute inflammatory conditions, such as ischemia-reperfusion injury, may impair this gate by downregulating the expression of JAM-C.^196,198^ Moreover, in vitro chemoattractants, such as CXCL8, can act as chemorepellents at higher concentrations,^199^ which allows for possible changes in neutrophil behavior as cells migrate along chemokine gradients. The relative levels of different lipid mediators may also be involved in neutrophil retrograde migration. Lipid mediator class-switching can lead to alterations in pro-inflammatory lipid mediator production pathways, which, in turn, produce pro-absorptive mediators, such as the production of LTB_4_ shifting to the production of LXA_4_.^200^ Apart from reverse migration, senescent neutrophils exhibit heightened sensitivity to CXCL12 in the bone marrow by upregulating CXCR4 expression on the membrane surface. In this process, senescent neutrophils are directed back to the bone marrow for homing, where they undergo apoptosis and are subsequently cleared by stromal macrophages.^201^ It is indisputable that reverse migration is a possible mechanism for the local resolution of inflammation and a potential new target for the pharmacological treatment of diseases characterized by neutrophil over-infiltration.

### Tumor immunity

While the pro- and anti-tumor roles of neutrophils are partially incongruent, certain molecules, such as G-CSF and IL-6, have the capacity to evoke both types of responses.^202,203^ Neutrophil activity within the tumor microenvironment is modulated by various factors, including tumor type, disease stage, neutrophil maturation state, and tissue environment.^204^ Consequently, the traditional classification of neutrophils into N1 and N2 phenotypes fails to fully capture their functional roles in this context. Neutrophils exhibit exceptional plasticity in response to tumor signals and exhibit complex heterogeneity, necessitating further exploration.

Neutrophils exert their anti-tumor effects through the release of cytotoxic cytokines and the activation of immunogenic responses *via* direct interactions with tumor cells.^205–207^ Neutrophils can also contribute to tumor development by fostering local tumor initiation, enhancing proliferation, supporting angiogenesis, facilitating metastasis, and orchestrating immunosuppressive networks in the tumor microenvironment.^208–210^ In particular, neutrophils within the tumor microenvironment induce the overexpression of immune checkpoint ligands, contributing to immunosuppression. Tumor-derived factors, such as GM-CSF, TNF-α, IL-6, HMGB1, and CCL20, upregulate PD-L1 expression on neutrophils, suppressing T cell activity and facilitating immune evasion by the tumor.^211–213^ PD-L1, a major ligand for the immunosuppressive receptor PD-1, serves as a pivotal immune checkpoint that modulates the activation threshold of immune cells in response to antigens.^214^ The binding of PD-L1 to PD-1 inhibits T cell function by abrogating their capacity to detect and eliminate tumor cells, facilitating immune evasion.^215^ Additionally, both tumors and normal tissues express CD47, a “don’t eat me” signal. SIRPα, a myeloid-inhibitory receptor on neutrophils, binds to CD47 on tumor cells to restrict antibody-dependent cellular cytotoxicity (ADCC), enabling tumor immune escape.^216^ These pathways through which neutrophils exert anti-tumor and pro-tumor effects have been comprehensively reviewed elsewhere.^65,75,217,218^ Moreover, previous studies have shown that NETs promote cancer metastasis by trapping circulating tumor cells.^219,220^ The DNA in NETs binds to CCDC25, triggering the ILK-β-parvin-RAC1-CDC42 cascade, promoting liver metastasis in breast and colon cancers.^221^ NETs also enhance colorectal cancer metastasis by releasing NE to activate ERK signaling.^222^ NE and MMP9 from NETs remodel laminin, an important component of the extracellular matrix (ECM), activating integrin α3β1 signaling and promoting proliferation of dormant cancer cells.^223^ ECM remodeling is essential for cancer cell initiation, invasion, and metastasis.^224^ Various enzymes released by NETs and TANs facilitate ECM remodeling, underscoring neutrophils’ role as contributors to tumor pathology. NETs may also protect tumor cells from cytotoxic T cells, potentially compromising anti-PD-1 blockade therapy.^225^ Inhibition of NETosis with PAD4 and DNase I inhibitors has shown promise in enhancing CD8^+^ T cell infiltration, reducing tumor growth, and improving the cellular response to anti-PD-1 blockade therapy in colorectal cancer.^226^

Neutrophils display significant complexity and plasticity in the tumor microenvironment. Understanding the role of TANs as either beneficial or detrimental depends on factors, including cancer stage, type, tissue specificity, and immune microenvironment. Herein, this review focuses on studies investigating epigenetic regulation and phenotypic analyses of TANs using high-resolution techniques including single-cell sequencing, time-of-flight cell counting (CyTOF), and mass spectrometry cytometry. High-resolution single-cell mapping across different tumor types has illuminated the diversity and plasticity of TANs.^227–229^ For instance, CD71, a marker associated with neutrophil proliferation, is expressed in subpopulations detectable in the circulation of patients with melanoma and non-small cell lung cancer.^230^ TANs expressing CCL4 recruit SSP1^+^ macrophages, while TANs expressing PD-L1 inhibit T cell cytotoxicity in liver tumors, highlighting potential therapeutic targets among pro-tumor TANs.^231^ In pancreatic ductal adenocarcinoma, TANs overexpressing BHLHE40 exhibit pro-tumor and immunosuppressive functions.^232^ Similarly, overexpression of the transcription factor *SOX2* in squamous lung cancer promotes TAN accumulation *via* upregulating of CXCL5, the murine homolog of human CXCL6.^233^ A subset of TANs distinguished by heightened expression of IFN-stimulated genes (ISGs)^227,231^ demonstrates anti-tumor activity and responsiveness to specific stimuli,^204^ suggesting immunotherapeutic potential. Interestingly, neutrophils expressing HLA-DR and CD74 have been identified as alternative antigen-presenting cells (APCs) in various cancer types.^234^ A previous study also reported that coupling anti-FcγRIIIB with antigens converted neutrophils into APCs,^235^ revealing novel therapeutic avenues in cancer treatment. Collectively, high-dimensional single-cell profiling and cell sorting techniques reveal neutrophil heterogeneity in cancer, decoding complex microenvironments. In terms of epigenetic regulation, neutrophils display a variety of phenotypes within tumors and exert their respective regulatory effects. Single-cell profiles that have been constructed for various tumor types enable the integrated analysis of neutrophil phenotypes across different cancers, paving the way for identifying common therapeutic targets.

Based on comprehensive single-cell analysis, a recent study classified TANs into three subgroups: T1, T2, and T3. T1 represents less mature neutrophils, T2 denotes mature neutrophils, and T3 is comprised of a mixed subtype with varying degrees of maturity.^7^ Another study observed large numbers of activated yet abnormally immature neutrophils in the circulation of patients with highly aggressive gastric cancer through single-cell RNA sequencing.^236^ Both immature (T1) and mature (T2) neutrophils in the tumor converged towards moderately mature (T3) neutrophils expressing dcTRAI1-R1. T3 neutrophils have an extended lifespan within the tumor, persisting for more than 5 days, and are predominantly localized in unique hypoxic glycolytic niches within tumors, where they enhance pro-angiogenic functions by expressing high levels of VEGFa.^7^ Collectively, this study suggested that the tumor microenvironment can deliberately manipulate neutrophil reprogramming, transforming immature (T1) and mature (T2) neutrophils into the pro-tumorigenic T3 phenotype, capable of promoting tumor proliferation and metastasis.^86^ Importantly, this neutrophil reprogramming is not restricted by tumor type or species,^7^ suggesting that therapeutic strategies aimed at limiting this differentiation could have broad applicability across different tumor types. Therefore, future research should prioritize validating the feasibility of interfering with tumor-mediated neutrophil reprogramming, and investigate whether strategies such as targeted drug therapies, immune induction training, and gene editing, can prevent the development of T3 neutrophils.

## Neutrophil receptors

Neutrophils display a diverse array of surface receptors, such as G protein-coupled receptors, Fcγ receptors, Toll-like receptors, receptor tyrosine kinases, and various cytokine receptors.^17^ Cytokine receptors, including IL-1R, IL-6R, and members of the interferon receptor family, belong to the class I cytokine receptor family. These receptors initiate signal transduction through associated Janus kinase (JAK) proteins, as they lack intrinsic kinase activity.^237^ Here, these receptors on neutrophils in both health and disease will be explored. Specifically, it will elucidate how these receptors activate downstream signal transduction pathways to regulate neutrophil functions.

### G protein-coupled receptors in neutrophil function

Heterotrimeric G proteins are composed of three subunits: α, β, and γ. The β and γ subunits form a tightly associated complex, often acting as a single functional entity. G protein-coupled receptor (GPCR) is a 7-transmembrane protein receptor containing a G protein-binding region in the cytoplasm and a ligand-binding region outside the cell. As early as the 1950s, the existence of this class of receptors was revealed by studies of the mechanisms by which the glucagon and adrenaline signaling pathways lead to elevated rates of glycogen metabolism. The N-terminus of GPCR is located extracellularly, whereas its C-terminus contains threonine and serine residues that promote the phosphorylation and intracellular signal transduction of downstream substrates *via* the G protein α-subunit in vivo.^238^ Following chemoattractant binding, GPCR facilitates the exchange of guanosine diphosphate (GDP) for guanosine triphosphate (GTP) on the Gα subunit, leading to its dissociation from the Gβγ dimer. This separation generates two active signaling entities, Gα and Gβγ, both of which engage distinct cellular pathways. Previous studies have indicated that the predominant pathway for GPCR-mediated signal transduction in neutrophils is facilitated through the Gβγ subunit.^239,240^ Another study also demonstrated that the plasma membrane of neutrophils lacking the Gβ subunit was dilated, thereby affecting cellular functions, including cell migration and phagocytosis.^241^

Neutrophils, as central players in both pathological and physiological inflammation, migrate toward target sites through a process governed by chemotaxis, relying on cell surface receptors to sense gradients of chemotactic agents.^242^ Most neutrophil chemoattractant receptors are GPCRs that strongly activate neutrophil chemotactic migration.^243–245^ The binding of chemoattractants to GPCR activates the pertussis toxin-sensitive heterotrimeric G proteins of the Gα_i/o_ family, signaling to downstream pathways.^17^ While each of these receptors is capable of inducing chemotaxis, the cellular responses they elicit vary. Distinct chemoattractants mediate unique migratory patterns through their respective receptors. Alterations in the function and expression of these chemoattractant receptors are closely linked to the pathogenesis of various diseases.

#### Chemotactic lipids receptors in neutrophil function

Leukotriene B_4_ type 1 receptor (BLT1) are GPCRs specific to LTB_4_.^246,247^ BLT1, which exhibits a high affinity for LTB_4_, is abundantly expressed in immune cells and participates in their activation and migration.^246,248^ LTB_4_ can enhance neutrophil accumulation in acute inflammation or injury by directly recruiting neutrophils through BLT1, or by serving as a relay signal that sustains a chemotactic gradient, guiding neutrophil migration toward other chemoattractants, including formyl peptides or C5a.^249,250^ Further research found that BLT1-mediated intracellular signaling is contingent upon the specific G protein subtypes expressed in various cell types. In granulocytes, LTB_4_-driven signaling predominantly occurs *via* Gα_i2_, while Gα_i1_ and Gα_o_ are abundantly expressed in the nervous system.^251^ LTB_4_ binding to its receptor BLT1 triggers the activation of G proteins, which in turn stimulate a cascade of kinases responsible for the phosphorylation of downstream signaling proteins.^252,253^ For instance, BLT1-mediated neutrophil chemotaxis necessitates the activation of PI3K and ERK pathway, both of which are crucial for establishing cell polarity and facilitating directed motility.^254–261^ Moreover, LTB_4_ has been demonstrated to upregulate the expression of pro-inflammatory cytokines implicated in atherosclerosis, primarily through the activation of the JNK, ERK, and NF-κB pathways.^262^ Recently, the receptor for advanced glycosylation end-products (RAGE) has emerged as a novel regulator of BLT1.^263^ RAGE promotes LTB_4_-driven neutrophil migration by enhancing ERK phosphorylation and inhibits LTB_4_-BLT1-mediated NF-κB activation and the upregulation of inflammatory cytokines. Positive and negative regulation of the LTB_4_-BLT1 axis by RAGE are both orchestrated through ERK phosphorylation.^264^ These insights underscore the pivotal role of the LTB4-BLT1 axis in the homeostatic control of neutrophils and highlight its potential involvement in neutrophil-associated chronic inflammatory diseases.^265^

#### Complement receptors in neutrophil function

C5a mediates its effects through two GPCRs: C5aR1 (CD88) and C5aR2 (C5L2, GPR77).^266^ C5aR1 and C5aR2 are broadly expressed across various myeloid cells, including eosinophils, neutrophils, mast cells, and macrophages.^266–268^ Recently, C5aR expression has also been identified in a range of non-myeloid cells across multiple organs, with notable abundance in the lungs and liver.^269,270^ C5aR1 belongs to the superfamily of GPCRs, which couples to the Gαi subtype of heterotrimeric G proteins.^271^ However, C5aR2 does not bind G proteins and is unable to initiate G protein-dependent signal transduction.^272^ C5a may induce a conformational change in C5aR1 that triggers the binding of C5aR1 to G proteins.^273^ Activated G proteins further dissociate to form α and βγ subunits, which subsequently activate different signaling cascades involving AC,^274^ phospholipase D (PLD),^275^ PKC,^276^ and PI3K.^277^ Moreover, increased intracellular calcium levels activate MAPK/ERK^276^ and NF-κB signaling pathways.^278^ These pathways lead to granzyme release, ROS production,^279^ and the activation of transcription factors, including STAT3,^280^ NF-κB, activator protein 1 (AP-1),^281^ and cAMP-responsive element-binding protein (CREB),^277^ which further contributes to the expression of pro-inflammatory cytokines, co-stimulatory molecules, and complement components.^282–285^

Targeting C5aR1 signaling presents a promising strategy to curb excessive neutrophil recruitment and activation. It has been shown that C5aR1 inhibition reduced neutrophil infiltration and recruitment in hepatic ischemia-reperfusion injury, accompanied by a diminished release of inflammatory mediators.^286^ In the model of necrotizing crescentic glomerulonephritis induced by anti-neutrophil cytoplasmic autoantibodies, mice that underwent bone marrow transplantation from C5aR1-deficient donors exhibited a marked reduction in the number of neutrophils infiltrating the glomeruli.^287^ Anti-C5aR1 treatment reduced neutrophil counts in bronchoalveolar lavage fluid and was correlated with improved airway hyperresponsiveness in mice.^288^ C5aR1 signaling was also found to be critical for pathological neutrophil recruitment during high-fat diet-induced vascular inflammation in murine models.^108^ This indicated an unrecognized role for C5aR1 in some vascular inflammatory diseases, including atherosclerosis and diabetic angiopathy. Thus, exploring the C5a-C5aR1 axis in neutrophil signaling pathways can contribute to understanding neutrophil-associated chronic inflammatory diseases.

#### Chemokine receptors in neutrophil function

Chemokine receptors, belonging to the class A family of GPCRs, are primarily coupled with the Gα_i_ subunit of heterotrimeric G proteins. These receptors are categorized into four distinct subfamilies, based on the classification of their principal chemokine ligands.^289^ Neutrophils are generally accepted to have limited chemokine receptor expression, consisting mainly of the CXC group.^290^ CXCR2 and CXCR4 are the primary receptors for CXCL8 and CXCL12, respectively. CXCL8 can also combine with CXCR1. The binding of a ligand and receptor activates downstream signal transduction, exerting regulatory effects.

CXCR2 is a major chemokine receptor expressed by neutrophils.^291,292^ It was initially discovered by Samanta in 1989.^293^ Both CXCR1 and CXCR2 contribute to neutrophil activation, they fulfill distinct, non-redundant functions in the context of inflammation and infection. Knockout mouse models have underscored the critical role of CXCR2 in driving inflammatory diseases characterized by neutrophil infiltration and activation.^294,295^ Specifically, although the majority of studies indicate that CXCR2 knockout mice appear generally healthy, these mice exhibit significant deficiencies in angiogenesis and wound healing, heightened susceptibility to pathogenic infections, and diminished pathogen clearance, primarily attributed to impaired neutrophil recruitment.^296–298^ The pivotal function of CXCR1 in resistance to infection was demonstrated in humans harboring a variant of the CXCR1 gene, which correlated with heightened susceptibility to bacterial infections. Neutrophils from these individuals had impaired fungicidal capacities and degranulation.^299^ The level of CXCR2 expression on the surface of neutrophils is influenced by the disease state. In general, CXCR2 expression in neutrophils is elevated in inflammatory diseases. In contrast, diminished CXCR2 levels are observed in neutrophils from patients with diverse immune-mediated diseases. The expression of peripheral blood neutrophil CXCR2 in patients with hepatitis B virus-associated acute and chronic liver failure was reduced, which is associated with a poor prognosis.^300^ In patients with systemic lupus erythematosus, aberrant regulation of CXCR2 expression by neutrophils and reduced levels of CXCL8-induced responses were observed.^301^ Neutrophils from sepsis patients exhibit reduced levels of CXCR2 and inhibition of CXCL8-induced migratory responses.^302^ However, an upregulation of neutrophil CXCR2 expression was observed during the relapse phase of ocular leukoaraiosis.^303^ Beyond directing neutrophil migration to sites of inflammation, CXCR2 signaling facilitates neutrophil release from the bone marrow, counteracting CXCR4-mediated retention within the marrow.^107^

CXCR2 activates a number of G protein-induced downstream signaling cascades, exerting different biological effects. PLC/PKC affects cell function. MAPK/p38 and Ras/ERK promote cell proliferation and survival. JAK2/STAT3 involves cell migration and cell proliferation.^304,305^ PI3K/Akt induces cell migration. Specifically, PI3K functions as a key intracellular signaling mediator downstream of the CXCR2/CXCL8 axis within the PI3K/Akt signaling pathway. When Akt phosphorylation is induced, CXCR2 can promote neutrophil chemotaxis.^306^ Moreover, CXCR2 can regulate angiogenesis and migration, with its activation being a hallmark alteration in numerous malignancies.^307^ Elevated CXCR2 expression has been shown to significantly enhance vascular density in the tumor microenvironment of transgenic mice, whereas CXCR2 knockout models exhibit a marked reduction in vascular density.^308^ CXCR2-deficient mice demonstrate reduced susceptibility to spontaneous tumor development, such as melanoma, prostate and kidney cancers.^309–311^ In pancreatic cancer patients, the expression of CXCR2 is significantly elevated. Interestingly, the upregulation of CXCR2 is predominantly confined to neutrophils, with minimal expression observed in tumor cells.

#### Formyl peptide receptors in neutrophil function

The initial discovery of the fMet motif binding to FPR1, in response to bacterial and mitochondria-derived peptides, marked a pivotal step in unraveling the intricate G protein signaling pathways within neutrophils.^312^ Human neutrophils express FPR1 and FPR2, of which FPR1 binds fMLF specifically and rapidly, whereas FPR2 binds fMLF with low affinity. Thus, FPR1 mainly regulates chemoattractant-induced neutrophil chemotaxis. The FPR1 receptor is consistently expressed on the surface of resting neutrophils and undergoes rapid upregulated in response to diverse inflammatory stimuli, such as lipopolysaccharide (LPS), unmethylated CpG oligodeoxynucleotides, and tumor necrosis factor-alpha (TNF-α).^313,314^ Some cytokines or pathogen-associated molecules may affect the expression and signal transduction of FPR. IL-4 inhibits FPR1 expression on neutrophils.^315^ *S. aureus*-derived formyl peptide receptor-like 1 (FPRL1) inhibitory protein impairs FPR-mediated Ca^2+^ signaling and neutrophil chemotaxis.^316^ Notably, various drugs can interfere with FPR function; for instance, cyclosporins exhibit inhibitory activity against FPR1.^317^ Apart from its upregulated expression in inflammatory diseases, FPR1 is also highly expressed in neutrophils in other diseases, including colorectal cancer, emphysema, and Crohn’s disease.^318–320^ The swift upregulation of FPR1 expression following stimulation suggests the receptor’s preformed presence within the cell. Subcellular fractionation studies and analyses of neutrophil transcription in the bone marrow have demonstrated that FPR1 synthesis is initiated during the late stages of neutrophil maturation. The receptor is then stored in azurophilic granules and secretory vesicles, ready for rapid deployment upon cellular activation.^321,322^

### Toll-like receptors in neutrophil function

The Toll-like receptor (TLR) family serves as primary receptors for DAMP and PAMP recognition by neutrophils, mediating immune responses, and defending against pathogens.^323^ Activation of TLRs triggers diverse signal transduction pathways, including myeloid differentiation primary response 88 (MyD88)-dependent and independent pathways (TRIF-dependent pathway), involving NF-κB and MAPK signaling pathways, which regulate the release of multiple pro-inflammatory cytokines from neutrophils to initiate inflammatory responses.^324^ MyD88, an adapter protein that consists of an N-terminal death domain and a C-terminal Toll/IL-1 receptor (TIR) domain, mediates the activation of NF-κB and MAPK pathways. With the exception of TLR3, all TLRs predominantly signal through the MyD88-dependent pathway.^18^ In contrast, the TRIF-dependent pathway is specifically employed by TLR3 and TLR4 during bacterial infections. This pathway activates interferon regulatory factors (IRFs), particularly IRF3, driving the production of type I interferons, including IFN-α and IFN-β, which play a crucial role in orchestrating immune responses against viral infections.^325^ Type I IFNs modulate both the function and phenotype of neutrophils, exerting inhibitory effects on tumor progression.^326^ Specifically, IFN-β impedes neutrophil accumulation by downregulating the expression of CXCR2 and CXCR4 on tumor cells, thereby disrupting the binding of their corresponding ligands.^327^ In addition, IFN-β shortens neutrophil lifespan by inducing the generation of ROS, regulating the expression of apoptosis-related proteins, upregulating Fas receptor-dependent death signaling, and decreasing G-CSF expression.^68^ Type I IFNs have also been demonstrated to suppress tumor angiogenesis by regulating the expression of various pro-angiogenic cytokines, including MMP9, VEGF, and a range of CXCL chemokines (CXCL1, CXCL2, CXCL5, and CXCL8).^328^

LPS, a component of the Gram-negative bacteria cell wall, serves as a major trigger for the development of sepsis. When bacteria enter the organism, LPS is detected by TLR4 on the neutrophil surface, facilitated by accessory molecules, including MD-2, LPS-binding protein (LBP), and CD14.^329^ Following LPS binding, TLR4 undergoes dynamic translocation between intracellular vesicles and the plasma membrane. This interaction triggers the recruitment of TIRAP, a junction protein containing a TIR domain, also known as MyD88-adapter-like (MAL) protein. TIRAP facilitates the activation of the MyD88-dependent signaling cascade by engaging with the TLR4-MD2 complex. Subsequently, TIRAP orchestrates the recruitment of the Myddosome complex, comprising Interleukin-1 receptor-associated kinase (IRAK) family protein kinases and MyD88, mediating early phase activation of MAPK and NF-κB pathways. This engagement subsequently enhances the mRNA expression of pro-inflammatory cytokines.^330^ The TLR4-LPS complex is internalized from the plasma membrane to the endosome through a CD14-dependent mechanism, where it mediates late-phase activation of MAPK and NF-κB.^331^ Within the IRAK family protein kinases, IRAK1 plays a critical role in signal transduction. Activation of MyD88 adapter proteins induces IRAK1 phosphorylation, which enables phosphorylated IRAK1 to interact with TNF receptor-associated factor (TRAF) family proteins, promoting recruitment of NF-κB essential modulator (NEMO) and TAK1.^332^ NEMO, also referred to as IKK-γ, forms the IKK complex with IKKα and IKKβ. NEMO mediates the phosphorylation of IκB proteins by regulating the activity of the IKK complex, thereby translocating NF-κB into the nucleus.^333^ Transforming growth factor-β-activated kinase 1 (TAK1), a member of the mitogen-activated protein kinase kinase kinase (MAPKKK) family, activates MAPK through the MAPK kinase cascade.^334^

Previous studies have elucidated the crosstalk between TLR activation and the PI3K/Akt pathway.^335,336^ PI3K acts as an intrinsic negative regulator of interleukin 12 (IL-12) production in response to TLR signaling, thereby limiting excessive Th1 polarization.^335^ This suggests that the PI3K signaling pathway may negatively regulate TLR signaling to prevent the generation of an excessive immune response. Moreover, activation of PI3K/Akt can limit the apoptotic and pro-inflammatory effects of detrimental stimuli through endogenous compensatory mechanisms. For instance, LPS-induced myocardial protection against ischemia/reperfusion injury has been associated with a PI3K/Akt-dependent mechanism.^336^ Several herbal extracts have demonstrated the ability to mitigate inflammatory responses and prevent tissue damage by activating the PI3K/Akt pathway while inhibiting the TLR4/NF-κB pathway.^337,338^ In summary, the intersection of the PI3K signaling pathway with the MAPK and NF-κB pathways plays a pivotal role in mediating crosstalk with TLR activation.

### Fcγ receptors in neutrophil function

Fcγ receptors, a class of IgG receptors found on the surface of neutrophils, regulate and mediate a variety of immune responses by recognizing the constant region of various IgG class antibodies.^339^ Among FcγRs receptors, FcγRIIa and FcγRIIIb are predominantly expressed on human neutrophils, with FcγRIIIb being the most abundant on their surface.^340^ Binding immune complexes (ICs) to FcγRIIa activates neutrophils, triggering cellular responses such as degranulation, cytoskeletal reorganization, production of ROS and inflammatory mediators, and the generation of NETs. FcγRIIIb stimulation is more effective at inducing actin polymerization, β1 integrin activation, and NET formation than FcγRIIa.^341^

FcγRIIIb is a low-affinity glycophosphatidyl inositol (GPI)-anchored molecule lacking transmembrane and cytoplasmic signal transduction domains, functioning primarily through indirect mechanisms in immune responses.^19^ FcγRIIIb has not been shown to associate with other subunits, leaving the initial mechanisms of its signaling largely undefined. However, studies have described the signaling pathway by which FcγRIIIb activation facilitates the formation of NET.^342–344^ FcγRIIIb aggregation activates Syk and TAK1 kinases, initiating the MAPK/MEK/ERK signaling cascade. MAPK signaling activates NADPH oxidase to generate ROS, a critical step in promoting the formation of NET.^342^ FcγRIIIb aggregation also activates Transient Receptor Potential Melastatin 2 (TRPM2) *via* PKC and ROS, elevating intracellular calcium ([Ca^2+^]_i_). levels in human neutrophils.^345^ IC binding to FcγRIIIb is implicated in NET release, through a process involving Mac-1.^343^ The binding of ICs to FcγR drives immune cell clearance of pathogens or target cells via ADCC, with FcγRIIIb expression levels on the surface of neutrophils correlating inversely with cellular ADCC potency. Specifically, neutrophils with elevated FcγRIIIb expression demonstrate markedly reduced ADCC compared to those with lower FcγRIIIb levels, suggesting that FcγRIIIb may act as a decoy receptor, competing with other activating Fcγ receptors for IgG binding.^346^ Notably, a previous study has indicated that FcγRIIIb is a negative regulator of neutrophil-mediated ADCC against tumor cells, positioning it as a potential therapeutic target to enhance neutrophil-mediated tumor cell destruction.^347^ During homeostasis, FcγRIIIb functions in clearing spontaneously formed ICs from the vascular system.^341^ However, neutrophils can adopt a non-professional antigen-presenting cells (nAPC) role by phagocytosing antibody-antigen complexes formed by the binding of FcγRIIIb to anti-FcγRIIIb-antigen conjugate,^235^ suggesting potential applications in immunotherapy for cancer and infectious diseases. Given that neutrophil activation and chemotaxis significantly affect inflammation levels within the tumor microenvironment and immune infiltration, future studies on the role of FcγRIIIb in neutrophils should prioritize investigating its impact on anti-tumor immunity.

Despite its lower abundance compared to FcγRIIIb on the neutrophil surface, FcγRIIa has long been acknowledged as the principal IgG receptor responsible for initiating signaling in neutrophils.^340^ The activation signal for FcγRIIa is transduced by the immunoreceptor tyrosine-based activation motif (ITAM), located in its cytoplasmic tail, which undergoes tyrosine phosphorylation by Src family kinases upon receptor activation.^348^ The phosphorylated tyrosine residues serve as docking sites for splenic tyrosine kinase (SYK), initiating multiple signaling cascades including MAPK, PI3K, and phospholipase C (PLC)-γ. Neutrophils ultimately exhibit cell activation, calcium mobilization, chemokine release, ROS production, and migration.^349^ The affinity of FcγRIIa for IgG is modulated by the cell’s activation state. When resting neutrophils exhibit low binding affinity, activation by fMLF or IL-8 enhances FcγRIIa’s ability to efficiently bind IgG.^350^ Moreover, genetic variations in neutrophil FcγR, particularly polymorphisms in the *FCGR* motif, can alter IgG binding affinity, affecting the clearance of ICs.^349^ This highlights the correlation between the FcγRIIa single nucleotide polymorphism (SNP) and the onset of various diseases, such as SLE, Kawasaki disease, and rheumatoid arthritis.^351–353^ Notably, ICs formed by heparin-induced thrombocytopenia/thrombosis can interact with FcγRIIa on neutrophils to induce NETosis, ultimately leading to thrombosis.^354^ FcγRIIa is the sole FcγR expressed on human platelets, where it promotes platelet activation upon binding IgG secreted by tumor cells. Therefore, FcγRIIa constitutes a promising therapeutic target for the management and prevention of cancer and cardiovascular disease.^355^

Blocking FcγRIIa has been shown to diminish neutrophil-mediated cytotoxicity, highlighting its role as the major FcγR involved in the elimination of IgG-opsonized tumor cells.^356^ Therapeutic monoclonal antibodies targeting epidermal growth factor receptor (EGFR) or human epidermal growth factor receptor 2 (HER2), such as Cetuximab and Margetuximab, can further enhance anti-tumor effects by binding to FcγR to activate ADCC and cytophagy.^357,358^ Collectively, Fcγ receptors are integral to the induction of neutrophil-driven adaptive immunity, including inflammatory and tumor immunity, thereby shaping the organism’s immune response. Dysfunction and genetic variations in FcγR play a critical role in the pathogenesis of various diseases, positioning FcγR as a promising therapeutic target for immune modulation and the design of novel therapeutic strategies.

### Tyrosine kinases in neutrophil function

Tyrosine kinases, a class of signaling enzymes widely involved in cellular signal transduction, regulate physiological processes in mammalian cells such as growth, differentiation, and apoptosis through the phosphorylation of protein tyrosine residues.^359^ Tyrosine kinases are broadly classified according to their function as receptor tyrosine kinases (RTKs) and non-receptor tyrosine kinases. RTKs are a class of transmembrane receptor proteins that bind specific growth factors or hormones through their external structural domains, activating their intrinsic tyrosine kinase activity.^360^ Unlike RTKs, non-receptor tyrosine kinases do not require binding to extracellular ligands for activation and directly participate in regulating signaling pathways by phosphorylating target proteins.^361^ In neutrophils, tyrosine kinases regulate a complex signaling network in response to diverse extracellular stimuli including bacterial products, chemokines, and inflammatory mediators.^21^

RTKs have been identified in more than 50 species and classified into seven subfamilies, with the G-CSFR family extensively studied in neutrophils. Recently, the RTK c-MET—also known as hepatocyte growth factor receptor (HGF-R)—has been identified as an important regulator of neutrophil function within the realms of inflammatory responses and tumor immunity.^65^ Targeting HGF/MET signaling and inhibiting c-MET have emerged as promising therapeutic strategies to attenuate neutrophil-driven inflammation.^362,363^ Moreover, the *MET* gene is widely recognized as one of the most important oncogenes, with aberrant activation or overexpression of c-MET associated with various human cancers.^364^ Thus, c-MET also represents a target for cancer immunotherapy, with its function in human neutrophils increasingly understood in recent years. It has been shown that c-MET-dependent nitric oxide release by neutrophils effectively inhibited tumor growth and metastasis, indicating the pivotal role of MET in recruiting anti-tumor neutrophils.^365^ However, a subsequent study revealed that inhibiting c-MET can hinder the reactive recruitment of neutrophils from the bone marrow into tumor tissues, facilitating T cell infiltration into tumors, and potentially improving the efficacy of cancer immunotherapy.^366^ These divergent findings highlight a dual role for c-MET in neutrophil immune responses within the tumor microenvironment. Given the success of c-MET inhibitors in tumor immunotherapy, the role of this RTK in neutrophils and tumor immunity should be explored further.

The major non-receptor tyrosine kinases integral to neutrophil signaling include Src and Syk kinases, orchestrate the effector function of FcγR and adhesion receptors on neutrophil surfaces.^21^ Previous studies have shown that Src kinases, such as Lyn and Hck, play pivotal roles in integrin-mediated neutrophil activation,^367–369^ with combined deletion severely impairing neutrophil phagocytosis^370^ and abrogating their pro-inflammatory responses in autoimmune disorders.^371^ Src kinases also participate in GPCR-mediated signaling to regulate neutrophil functions through activating downstream pathways, such as PI3K, MAPK, and NF-κB.^372,373^ Thus, inhibition of Src family kinases presents a potential therapeutic strategy to reduce inflammation triggered by neutrophil activation. In contrast, Syk kinases are activated through the interaction of their tandem Src homology 2 domains with ITAM domains,^374^ primarily regulating the activation of neutrophils by ICs through FcRγ,^375^ essential against various pathogens including bacteria and fungi.^376,377^ Both Src and Syk kinases have also been implicated in various cancers and are considered promising targets for tumor-specific therapies.^378^ However, their role in regulating the tumor immune response in neutrophils remains largely unexplored, presenting a promising avenue for future research.

## Signal transduction pathways in neutrophils

Multiple downstream signal transduction pathways are activated following the binding of receptors on neutrophils to ligands. These pathways regulate various transcription factors through phosphorylation cascades, mediating numerous neutrophil functions such as release from bone marrow, chemotactic migration, phagocytosis, secretion of inflammatory mediators, and participation in tumor immune responses.^23^ Here, given that neutrophils primarily mediate inflammatory responses and tumor immunity, the mechanisms by which signal transduction pathways downstream of neutrophil receptors regulate chemotactic migration and tumor immunity will be elucidated.

Neutrophil chemotaxis is primarily regulated by three signaling pathways that govern cell polarity and cytoskeletal remodeling: the PI3K pathway, the ERK pathway, and the p38 MAPK pathway.^379–383^ Multiple chemoattractants modulate leukocyte polarity, adhesion, and motility. Through activation of intracellular signaling pathways *via* GPCRs, they orchestrate the establishment of biochemical asymmetry within the cell. This leads to the formation of polarized cells, characterized by actin-enriched leading edges and myosin II-dense trailing ends.^384,385^ Such polarization enables the efficient translation of cytoskeletal forces into directed cell-body displacement.^384^ These signals provide both the directional guidance for neutrophil migration and the stimulus required for polarization. At the core of neutrophil polarization lies the dynamic remodeling of cytoskeletal proteins. This intricate process is tightly regulated by numerous second messengers, kinases, and members of the Rho family of GTPases. A deeper understanding of signaling pathways that govern this cytoskeletal reorganization will significantly advance our knowledge of the cell biology underlying neutrophil polarity and migration.

### PI3K signaling pathway in neutrophils

**Fig. 4:**
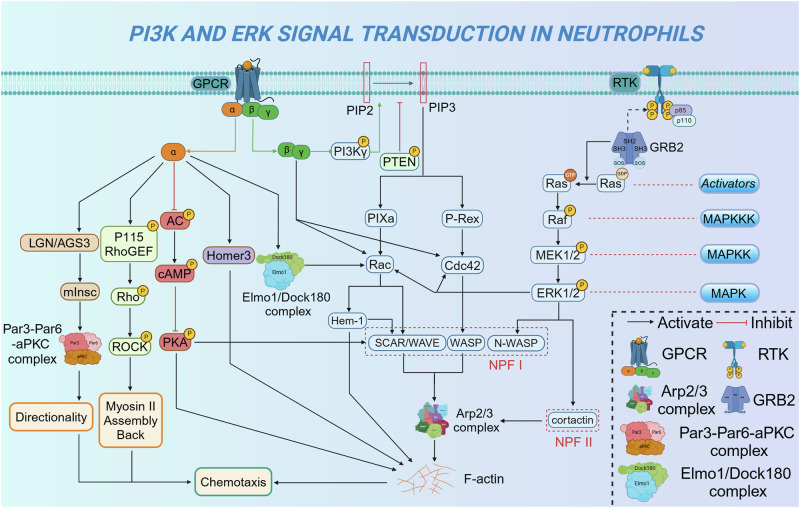
(1) Activation of downstream signaling pathways by chemoattractant receptors regulating neutrophil polarization and cytoskeletal rearrangement. The binding of GPCR to chemoattractants results in the dissociation of the Gα subunit from the Gβγ dimer. Both the Gα and Gβγ subunits signal to various cellular pathways. Activation of the PI3K pathway by the Gβγ subunit promotes F-actin remodeling. Activation of downstream signaling pathways by the Gα subunit synergizes with the PI3K pathway to regulate neutrophil chemotaxis. (2) Crosstalk between the PI3K and ERK signaling pathways. Both the PI3K pathway and the ERK pathway can target Rac and Cdc42, activating NPFs and the Arp2/3 complex and promoting F-actin remodeling. GPCR G protein-coupled receptor, PTEN Phosphatase and tensin homolog, NPF Nucleation-promoting factors, PI3K Phosphoinositide 3-kinase, ERK Extracellular signal-regulated kinase, MAPK Mitogen-activated protein kinase, WASP Wiskott-Aldrich syndrome protein, WAVE WASP-family of Veprin homolog, GRB2 Growth factor receptor-bound protein 2. Figure created with BioRender.com

The PI3K pathway has been identified as a major signaling pathway in the downstream signaling cascade of GPCRs within neutrophils^17,145,382^ (Fig. 4). PI3K consists of four catalytic subunits (p110α, β, δ, and γ) and other regulatory subunits (p101 or p85), of which δ and γ are present only in leukocytes, while the other two are widely distributed in a variety of cells.^386^ PI3Kγ primarily mediates neutrophil chemotaxis.^382^ Gβγ dimers act as transducers of chemotactic signals and interact with PI3Kγ in a Ras-dependent manner.^387^ Activated PI3K binds to phosphatidylinositol 4,5-bisphosphate (PIP2), a fundamental component of the cell membrane, which catalyzes the formation of phosphatidylinositol-(3,4,5)-trisphosphate (PIP3).^388^ PIP3 was found to be asymmetrically distributed at the leading edge of the cell. The currently accepted explanation for this is the presence of 3’ phosphatase and tensin homolog (PTEN) and 5’ SH2-containing inositol phosphatase (SHIP).^382^ PTEN catalyzes the hydrolysis of PIP3 to PIP2, effectively suppressing the PI3K signaling pathway.^389^ PTEN-mediated regulation appears to be highly context-dependent, with PTEN controlling chemotaxis in the presence of two opposing gradients and distinguishing between end-point and intermediate chemoattractants.^390^ PTEN is hypothesized to integrate the responses to multiple chemotactic signals and filter the optimal direction to reduce its own expression and activate PI3K.^390^ Conversely, SHIP-deficient neutrophils exhibit severe dispersion, with impaired polarization and diminished chemotactic capacity.^256,257^

In neutrophils, PIP3 facilitates the direct binding of Gβγ to P-Rex1 and PIXα, guanine nucleotide exchange factors (GEFs) for Rac and Cdc42.^391,392^ Moreover, PIP3 directly interacts with the binding structural domains of Rac and Cdc42, thereby promoting their activation.^382^ Rac, Cdc42, and RhoA are members of the Rho GTPase family, a critical subset of the Ras superfamily of GTPases. Guided by Rho GTPase signaling, migrating neutrophils orchestrate distinct cytoskeletal processes at the leading and trailing edges. Rac is localized to actin polymerization and leading edge protrusion, whereas Rho is localized to myosin and trailing edge contraction.^393^ Thus, the establishment and maintenance of polarity necessitates intricate crosstalk between Rho and Rac.^384,394^ Rac promotes anterior expansion and cell migration. Cdc42 participates in regulating cell polarity and the dynamic stabilization of microtubules. The activation of RhoA induces myosin-II filament contraction, while simultaneously promoting PTEN activation and suppressing Rac activity, both of which are crucial for the establishment of neutrophil polarization.^395,396^ Activated Cdc42 and Rac regulate the Wiskott-Aldrich syndrome protein (WASP) and the suppressor of cAMP receptor/WASP family verprolin-homologous protein (SCAR/WAVE) complex,^397^ which stimulate the Arp2/3 complex—essential for generating branched actin filaments and driving pseudopod extension.^140^ Rac has been shown to sustain neutrophil polarity *via* Hem-1, a protein family located exclusively at the leading edge of the cell. Hem-1 promotes actin polymerization while excluding myosin activity from the cell’s anterior; it also promotes anterior Rac activity in a positive feedback loop.^398^ WASP induces Cdc42-dependent actin cluster formation, promoting F-actin polymerization.^399^ Patients with Wiskott-Aldrich syndrome were found to exhibit defects in neutrophil chemotaxis.^400^ Actin polymerization at the cell’s leading edge drives the extension of broad, flattened lamellar pseudopods, which, together with the slender filamentous pseudopods, generate the forces that propel the cell to move. Activated Rac and Cdc42 establish a positive feedback loop, facilitating the recruitment of additional PIP3 at the cell’s leading edge.^401^ These data suggest that PI3K signal transduction mediates changes in neutrophil polarity for chemotaxis by regulating actin polymerization.

Fluctuations in intracellular Ca^2+^ levels are a defining feature of neutrophil activation. In resting neutrophils, cytosolic Ca^2+^ concentrations remain low but rapidly rise following stimulation, either through the release of Ca^2+^ from internal stores or the influx of extracellular Ca^2+^.^402^ This influx is predominantly facilitated by Ca^2+^ release-activated Ca^2+^ (CRAC) channels embedded in the plasma membrane. This process can be triggered by the activation of GPCRs in neutrophils.^403^ Gβγ dimers activate phospholipase Cβ (PLCβ), which converts PIP2 to diacylglycerol (DAG) and inositol 1,4,5 triphosphate (IP3 or InsP3). The IP3 receptor (IP3R) is situated within the endoplasmic reticulum membrane. Upon activation by IP3, this non-selective Ca^2+^ channel facilitates the release of Ca^2+^ from the endoplasmic reticulum stores into the cytoplasm, driving intracellular calcium signaling.^404^ Subsequently, Ca^2+^ flow activates protein kinase C (PKC), playing a pivotal role in driving ROS production through the NADPH oxidase complex.^405^ PKC also regulates cytoskeletal rearrangements, thereby affecting cell polarity and direction of movement.^406^ The release of Ca^2+^ shifts LFA-1 from a low-affinity state to a medium-high-affinity state, which leads to a slowing down of neutrophil rolling through LFA-1/ICAM-1 interaction.^407^ The canonical (TRPC) channels of transient receptor potential (TRP) channels are non-selective cation channels for Ca^2+^. TRPC channels were shown to influence chemokine receptor-mediated actin remodeling by modulating Ca^2+^ influx.^408^

G proteins are characterized by their Gα subunits, which are grouped into four distinct families based on sequence homology and functional attributes: Gα_s_, Gα_i/o_, Gα_q,_ and Gα_12_. Gα_s_ (s stands for stimulation) and Gα_i_ (i stands for inhibition) are ubiquitously expressed across various cell types.^20^ Gα_i/o_ family proteins inhibit adenylate cyclase (AC) activity, leading to reduced intracellular cAMP levels and therefore inhibiting the protein kinase A (PKA) signaling pathway.^20^ Inhibition of PKA was found to enhance chemotaxis-regulated neutrophil functions, such as superoxide production, chemotaxis, and adhesion. This underscores the regulatory influence of cAMP in orchestrating the cellular processes at the leading edge of the neutrophil.^409,410^ Additionally, PKA has been implicated in chemokine-induced cell polarization and caudal limb formation. Notably, cAMP analogs promote caudal peduncle development, while its formation is abrogated by the PKA-specific inhibitor.^411^ Gα_i2_ also interacts with the engulfment and cell motility 1/dedicator of cytokinesis 1 (Elmo1/Dock180) complex, which acts as a RacGEF and activates Rac proteins, inducing actin polymerization and cell migration.^412^ The Gα subunit had long been considered only a “timer” that controlled the initiation and termination of Gβγ signaling by regulating the release and recombination of Gβγ dimers.^413^ However, the Gα subunit has been shown to also function independently of the Gβγ subunit. At the leading edge of neutrophils, Gα_i_ family proteins can also bind two guanine nucleotide dissociation inhibitors, namely activator of G protein signaling 3 (AGS3) and Leu-Gly-Asn repeat-enriched protein (LGN), forming the Gαi-GDP-AGS3/LGN complex.^414^ The complex then binds to mInsc, leading to mInsc-mediated targeting of the Par3-Par6-aPKC complex to the leading edge of the pseudopods to regulate orientation during neutrophil chemotaxis.^415^ Moreover, a mammalian Gα effector that mediates neutrophil chemotaxis has recently been reported. Homer3, a recently identified Gα_i2_-interacting protein, orchestrates actin assembly to facilitate polarity and motility during neutrophil chemotaxis.^415,416^ Additionally, Gα_12/13_ activates Rho and its effector kinase ROCK through p115 RhoGEF, promoting myosin II assembly in the rear of chemotactic cells.^417,418^ Gα_i/o_ family proteins can also increase PI3K expression levels and activate the PI3K/Akt signaling pathway, promoting neutrophil functions other than chemotaxis.^145,259,419,420^ Thus, the Gα-mediated pathway appears to be important for signaling at both the leading and trailing edges of the cell and, together with the Gβγ-mediated pathway, regulates neutrophil chemotaxis.

Precise regulation of GPCR and heterotrimeric G protein signaling at multiple levels is critical for accurate chemotaxis. Both Gα and Gβγ engage distinct downstream pathways, yet some aspects remain unresolved, such as the nucleotide-binding state of Gα that mediates signal transduction, the role of Gβγ in modulating the PI3K signaling pathway, and the precise connection between heterotrimeric G protein activation and cytoskeletal reorganization. A deeper understanding of the regulatory mechanisms and intracellular targets of chemotactic receptors is of great importance, as it could illuminate disease pathogenesis at the cellular level and aid in the identification of novel therapeutic targets across various diseases.

### ERK signaling pathway in neutrophils

Neutrophils deficient in PI3Kγ are still capable of undergoing chemotaxis in substantial numbers,^421^ suggesting the involvement of additional signaling pathways beyond PI3K pathway. In fact, two important branches of the MAPK signaling pathway, the ERK and p38 MAPK pathway, also contribute to regulating neutrophil polarity and cytoskeletal rearrangement, affecting neutrophil chemotaxis^379^ (Fig. 4). MAPK, a proline-directed Ser/Thr-specific protein kinase, transmits extracellular signals through phosphorylation cascades, orchestrating coordinated cellular responses to environmental stimuli.^422^ To date, four distinct classes of mammalian MAPKs have been identified: extracellular signal-regulated kinases (ERK1 and 2), c-jun N-terminal kinases (JNK1-3), p38 MAPK, and ERK5.^423^ Activation of MAPK pathways is mediated through a conserved three-tiered kinase cascade: MAPKKK-MAPKK-MAPK.^424^

The cascade of the ERK signaling pathway is Ras-Raf-MEK1/2-ERK1/2, where Raf acts as MAPKKK, MEK1/2 as MAPKK, and ERK1/2 as MAPK.^381^ Ras, an activator of the ERK pathway, serves as a crucial upstream regulator in the Raf/MAPK cascade.^425^ Ras/Raf/MEK/ERK can be activated through distinct mechanisms: (1) a ligand-dependent route, where ligands, including hormones, growth factors, or cytokines, specifically bind to receptors and (2) a ligand-independent route, triggered by physical stressors like injury, radiation, or osmotic pressure.^426^ The activation of Ras is predominantly driven by ligand binding to receptor tyrosine kinases (RTKs) rather than GPCRs. Although chemoattractants mostly bind to GPCRs, their effects may indirectly activate RTK. Additionally, the activation of Ras may depend on PI3K activity.^427^ In conclusion, the combination of chemoattractants with GPCR may contribute to Ras activation in multiple ways. Growth factor receptor-binding protein 2 (GRB2), a key protein downstream of RTK, participates in numerous signal transduction pathways. GRB2 exhibits a distinctive “sandwich structure,” which consists of a central SH2 domain flanked by two SH3 domains on both sides.^428^ RTK activation results in the recruitment of GRB2 to phosphorylated tyrosine residues on the receptor *via* its SH2 structural domain.^380^ The SH3 structural domain of GRB2 establishes a linkage with the Son of Sevenless (SOS) protein, resulting in the translocation of SOS from the cytoplasm to the vicinity of the cell membrane.^429^ Ras activation is contingent upon the assembly of complexes involving GRB2, autophosphorylated growth factor receptors, and SOS.^430^ In their normal resting state, Ras proteins remain in an inactive GDP-bound conformation.^431^ SOS acts as a GEF, and the binding of the GRB2-SOS complex to Ras facilitates the transition of the inactive form of Ras (Ras-GDP complex) to an active state (Ras-GTP complex) within the cell membrane.^432^ GRB2 also plays a crucial role in connecting RTK and the PI3K signaling pathway.^433^ Then, Raf and other downstream targets recruited by Ras-GTP are activated. The Raf protein family comprises three Ser/Thr kinases, including ARaf, BRaf, and CRaf, which serve as critical mediators linking membrane-bound Ras-GTPases to downstream kinases in the MAPK signaling pathway.^434^ CRaf interacts with MEK through the phosphorylation of active sites (Ser338 and Tyr341).^435^ Interestingly, the phosphorylation of Tyr341 and Ser338 has been recognized as a pivotal regulator of Raf kinase activity.^436^ MEK, a dual-specificity kinase family targeting both tyrosine and Ser/Thr residues, promotes ERK activation by phosphorylating regulatory tyrosine and threonine sites. The catalytic VIII subregion of Raf interacts with MEK through its C-terminal catalytic domain, which phosphorylates serine residues, initiating MEK1/2 activation.^435^ ERK, a Ser/Thr protein kinase, plays a pivotal role in orchestrating cellular signal transduction pathways. It activates a variety of downstream target proteins that regulate cellular functions, and malfunctions in its activation are associated with the onset of a variety of diseases, including cancer, inflammation, metabolic disease, and cardiovascular disease.^437^ MEK interacts directly with ERK through its N-terminal region. Specifically, it activates ERK by catalyzing the dual phosphorylation of tyrosine and threonine residues within the eight conserved “TEY boxes” of the ERK subfunctional domain. Once activated, ERKs translocate to the nucleus, where they enhance the phosphorylation of cytoplasmic target proteins or modulate the activity of other protein kinases.^438^

Among the numerous ERK1/2 target proteins, nucleation-promoting factors (NPFs) are thought to be mainly involved in neutrophil chemotaxis. As mentioned previously, the Arp2/3 complex promotes actin polymerization, which is required for pseudopod formation. The Arp2/3 complex itself has only weak actin nucleation activity, which is enhanced by interaction with NPFs.^439^ NPFs are divided into two classes: class I NPFs and class II NPFs. Class I NPFs are further divided into five subclasses: the WASP and neural WASP (N-WASP); the WASP family of Veprin homologs (WAVE); WASP and SCAR homologs (WASH); WASP homolog associated with actin, membranes, and microtubules (WHAMM); and junction-mediated regulatory protein (JMY). The major member of class II NPFs is cortactin.^440^ WAVE promotion of the actin-nucleating activity of the Arp2/3 complex is required for the formation of a large complex called the WAVE regulatory complex (WRC). The complex scaffolds actin monomers and the Arp2/3 complex.^439,441^ ERK directly phosphorylates T346, S343, and S351 of WAVE2 in the WAVE family. ERK also phosphorylates Abl-interacting protein 1 (Abi1; a component of the WRC), which is required for the interaction of the WRC with the Arp2/3 complex and actin, suggesting that ERK promotes WAVE2-mediated actin polymerization.^442,443^ Cortactin, a class II NPF, is also involved in actin polymerization. Beyond its direct interaction with the Arp2/3 complex to enhance actin nucleation, cortactin also associates with N-WASP and WASP through its SH3 domain, synergistically activating the Arp2/3 complex.^444^ In addition to the actin nucleation factors, actin elongation factors, such as Enabled (Ena)/vasodilator-stimulated phosphoprotein (VASP) family proteins, also govern the extension of filopodia and lamellipodia. These elongation factors bind to the barbed ends of F-actin, preventing capping proteins from terminating filament elongation.^440^ ERK can also regulate actin polymerization by affecting Rho family GTPases. ERK phosphorylates Cdc42 GTPase-activating protein (CdGAP) at T776, leading to its functional inactivation.^445^ This implies that ERK can indirectly activate Cdc42 and Rac at the leading edge, promoting actin polymerization.

Cell polarization is characteristic of neutrophil chemotaxis triggered by chemotactic stimuli. This pivotal biological process is caused by the activation of numerous signaling cascades involving proteins such as GTPases, kinases, and the Rho family. These signaling molecules orchestrate the dynamic remodeling of actin cytoskeleton, facilitating directed cellular movement. The regulation of this process was predominantly attributed to the PI3K and ERK signaling transduction. They interact and influence each other and synergistically regulate cytoskeletal dynamics by phosphorylating downstream targets involved in actin remodeling. In fact, the biological processes that regulate cell polarity and migration through signal transduction are not limited to neutrophils but are also observed in other immune cells, somatic cells, and neuronal cells. The pathological changes caused by dysregulated signal transduction are not limited to inflammatory diseases but have also been demonstrated in cancer, autoimmune diseases, and metabolic diseases. Elucidating these pathways will pave the way for the development of more targeted pharmacological strategies to treat diseases marked by disrupted neutrophil polarity.

### P38 MAPK signaling pathway in neutrophils

**Fig. 5:**
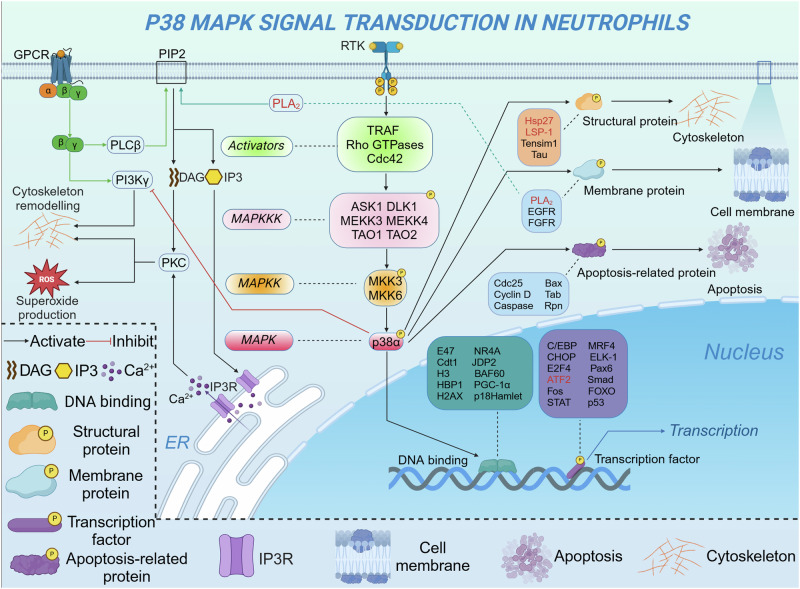
Overview of the P38 MAPK-mediated regulation of neutrophil chemotaxis and the biological functions of P38 MAPK signaling. (1) P38 MAPK-activated kinase phosphorylates many targets involved in actin microfilament dynamics, such as Hsp27, ATF-2, PLA_2_, and LSP-1. (2) PLA_2_ converts PIP2 to DAG and IP3, like PLCβ, to activate PKC, regulating superoxide production and cytoskeletal rearrangement. (3) The P38 MAPK pathway inhibits the PI3K pathway. Hsp27 Heat-shock protein 27, ATF-2 Activating transcription factor 2, LSP-1 Lymphocyte-specific protein 1, PLA_2_ Phospholipase A_2_, PLCβ Phospholipase C-beta, PIP2 Phosphatidylinositol 4,5-bisphosphate, PIP3 Phosphatidylinositol 3,4,5-trisphosphate, IP3 Inositol trisphosphate, DAG Diacylglycerol. Figure created with BioRender.com

Interestingly, although chemoattractant receptors all regulate the downstream signaling pathways of GPCR, different chemoattractants mediate various intensities of neutrophil chemotaxis. In fact, neutrophils exhibit a hierarchical response to chemoattractants, allowing them to effectively navigate complex environments characterized by overlapping gradients.^43^ Typically, host-derived signals like CXCL8 and LTB4 serve as “intermediary” cues, directing neutrophils out of the vasculature and towards the general vicinity of their target. However, once neutrophils encounter “end-target” signals, such as fMLF and C5a, these intermediary cues are disregarded. The end-target signals, often derived from injury or pathogens, focus neutrophil cytotoxic activity precisely on the relevant targets. Neutrophils have been observed to preferentially migrate toward end-target chemoattractants, even in the presence of elevated concentration of intermediary signals,^446^ implying that the end-target chemoattractant receptor may regulate a more prioritized signaling pathway compared to the intermediary chemoattractant receptor. The fact that the PI3K/Akt signaling pathway predominantly mediates neutrophil chemotaxis has been demonstrated.^383^ PI3K drives neutrophil polarization primarily by regulating downstream PIP3 production *via* the catalytic isoform p110γ. This is exemplified by the absence of phosphorylation and activation of PI3K pathway in response to chemotactic receptor engagement in p110γ-deficient mice, resulting in impaired chemotaxis.^421^ However, neutrophils from p110γ-deficient mice efficiently migrated to N-formyl-Met-Leu-Phe (fMLP) with normal rates and directions,^447^ implying that the generation of PIP3 is not required for fMLP-directed chemotaxis. Previous studies demonstrated that end-target molecules induced neutrophil chemotaxis *via* the p38 MAPK pathway, in contrast to intermediary chemoattractants, which relied on the PI3K/Akt pathway.^446,448^ Although fMLP also activated PI3K to a lesser extent than CXCL8-induced expression, the concomitant addition of chemokines and fMLP resulted in the inhibition of PI3K activity, indicating that the p38 MAPK pathway may inhibit the PI3K pathway (Fig. 5). Activation of the p38 MAPK pathway is also orchestrated through a triple kinase cascade. p38 MAPK-activated kinase phosphorylates many targets involved in actin microfilament dynamics, such as heat-shock protein 27 (Hsp27), activating transcription factor 2 (ATF-2), and LSP-1,^449^ which may explain the fact that the p38 MAPK pathway regulates neutrophil chemotaxis. Formyl peptides have been shown to selectively activate phospholipase A2 (PLA_2_) in neutrophils phosphorylated by p38 MAPK-activated kinase, and PLA_2_ inhibitors block neutrophil chemotaxis toward formyl peptides but not toward CXCL8.^390^ PLA_2_ also converts PIP2 to DAG and IP3, like PLCβ, to activate PKC,^450^ regulating superoxide production and cytoskeletal rearrangement. Moreover, the fMLP-induced antagonism of PI3K activity might be PTEN-dependent.^390^ PTEN, localized in the uropod of neutrophils, converts PIP3 to PIP2, promoting cell polarity formation. When neutrophils were simultaneously exposed to opposing chemoattractants, such as CXCL8 and fMLP, PTEN was observed to redistribute across the cell membrane in a p38-dependent manner. While the precise mechanism underlying this hierarchical chemoattractant-induced PTEN redistribution remains unclear, it is likely to contribute to reduced PIP3 levels at the leading edge, thereby promoting neutrophil migration toward PI3K-independent chemoattractants.^390^ However, the mechanisms by which fMLP suppresses the PI3K pathway *via* p38 MAPK remain elusive, as does the reason behind the co-stimulation of chemokines and fMLP leading to PTEN redistribution across the cell membrane. More efforts should be made to investigate the mechanism wherein the p38 MAPK pathway does not rely on PIP3 to regulate neutrophil-directed chemotaxis. Perhaps downstream crosstalk between the p38 MAPK and PI3K pathways is the key to the difference in chemotactic responses of neutrophils to high- and low-priority chemoattractants. Researchers have also proposed a hypothetical model in which the rapid desensitization of low-priority chemotactic receptors results in their signaling being overwhelmed by the persistent signaling output of high-priority receptors.^242^ It is certain that the precise direction of chemotaxis is determined by neutrophils through the perception of complex chemoattractant signals in tissues, ensuring they accurately reach areas of infection or inflammation to exert immune defenses.

### JAK/STAT signaling pathway in neutrophils

**Fig. 6:**
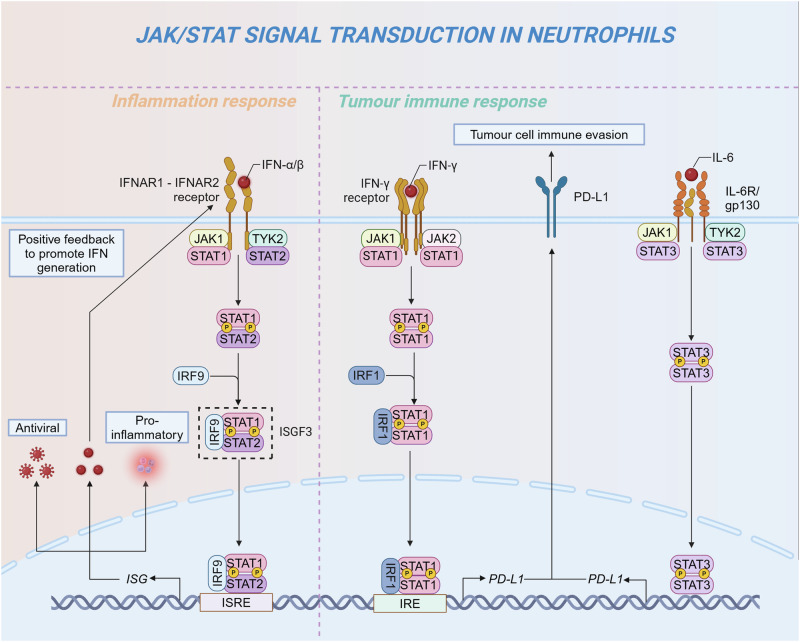
Overview of JAK/STAT-mediated regulation in neutrophil inflammatory and tumor immune responses. (1) Type I IFNs positively regulate their own production, exert antiviral effects, and activate inflammatory responses by activating JAK/STAT signal transduction in neutrophils. (2) IFN-γ and IL-6 upregulate PD-L1 expression through JAK/STAT signal transduction, inhibiting T cell function to promote immune evasion by tumor cells. JAK Janus kinase, STAT Signal transducer and activator of transcription, IFN Interferon, IRF9 IFN regulatory factor 9, ISGF3 IFN-stimulated gene factor 3, ISRE IFN-stimulated response element, ISG IFN-stimulated gene, IRE IRF1 response element, PD-L1 Programmed cell death ligand 1. Figure created with BioRender.com

The Janus kinase/signal transducer and activator of transcription (JAK/STAT) signaling pathway is a critical regulator of neutrophil function. Approximately 70 cytokines, encompassing interleukins, interferons, colony-stimulating factors, and growth factors, exert their regulatory influence predominantly *via* the JAK/STAT pathway, serving as the principal mechanism for initiating transcription.^451^ This pathway mediates multiple downstream events, including inflammation, hematopoiesis, tissue repair, cancer immunity, apoptosis, and adipogenesis.^452^ The JAK family consists of four non-receptor tyrosine kinases: JAK1, JAK2, JAK3, and TYK2. Of these, JAK1, JAK2, and TYK2 are expressed in all tissues and cell types,^453^ while JAK3 expression is typically restricted to myeloid and lymphoid tissues, with high expression in activated T cells, B cells, and monocytes.^454^ The STAT family consists of nuclear transcription factors classified into seven subtypes which regulate various cellular responses, including proliferation, apoptosis, and immunity, with neutrophils predominantly expressing STAT3.^455^ Cytokine binding to its receptor on the neutrophil membrane causes receptor dimerization. This ligand-receptor interaction induces JAK transphosphorylation, activating JAK, which then phosphorylates tyrosine residues on the receptor, forming the docking site for STAT proteins.^456^ At this docking site, JAK phosphorylates STAT, causing STAT to dissociate from the receptor and form homodimers or heterodimers through SH2 domain-phosphotyrosine interactions. These dimers translocate to the nucleus, bind the target gene promoter, and regulate transcription of the target gene.^455^ Three major cytokines or cytokine families—G-CSF, interleukins, and interferons—bind to their respective receptors on the neutrophil surface and regulate neutrophil function *via* the JAK/STAT pathway.^457^ G-CSFR is an RTK, while most interleukin receptors and interferon receptors belong to class I cytokine receptors in the insulin-like receptor family.^458^ The regulatory role of G-CSF in neutrophils is described in Section 3. Here, this review focuses on the effects of interferons and interleukins on neutrophils through JAK/STAT signaling (Fig. 6).

Initially described as cytokines with antiviral properties, interferons (IFNs) have also been shown to mediate tumor immune responses more recently.^459^ Three types of IFNs have been identified: types I, II, and III. Type I (IFN-α/β) and type III (IFN-λ) IFNs are produced by a wide range of cells, whereas type II IFN (IFN-γ) is primarily produced by T cells and natural killer (NK) cells.^460^ Each type of IFN binds to specific cell surface receptors to initiate JAK/STAT signaling, exerting distinct biological effects.^461^ Type I IFNs bind to the heterodimeric receptor IFNAR, inducing JAK1 and TYK2 phosphorylation. JAK recruits and phosphorylates STAT to form STAT1 and STAT2 heterodimers. The STAT heterodimers then bind to IRF9 to form a complex known as the IFN-stimulated gene factor 3 complex (ISGF3).^462^ ISGF3 translocates to the nucleus, where it binds to the IFN-stimulated response element (ISRE) to regulate the transcription of ISGs.^463^ Type I IFNs exert antiviral effects through JAK/STAT signaling. The type I IFN-mediated immune response is summarized in three phases: early, intermediate, and late.^464,465^ Initially, innate immune cells recognize pathogens and activate intracellular signaling *via* PRRs to upregulate IFN-β and ISG expression.^466^ In the intermediate phase, IFN-β activates JAK/STAT signaling, leading to the transcription of IRF7, which induces the expression of IFN-α. The late phase involves a positive feedback loop where IRF7 and IRF3 induce the expression of IFN-α and -β genes, respectively, resulting in large amounts of ISG and a robust antiviral response.^464^ Amplification of IFN α/β genes during the late phase enhances the pro-inflammatory response, increasing the infiltration of type I IFN-producing effector cells, further amplifying the inflammatory response.^467^ Dysregulated type I IFNs and aberrant neutrophils have been implicated in vascular injury due to systemic autoimmune disease.^468^ Type I IFNs also exert anti-tumor effects through JAK/STAT signaling. They reduce neutrophil lifespan by upregulating the IFN-β-dependent death receptor Fas and regulating anti-apoptotic and pro-apoptotic protein expression to achieve a pro-apoptotic ratio.^469^ Moreover, Type I IFNs interfere with neutrophil recruitment by inhibiting the expression of CXCR2 and CXCR4 in tumor cells, preventing ligand binding.^328^ Type I IFNs also suppress tumor proliferation by inhibiting pro-angiogenic chemokines such as VEGF, MMP9, and CXCR2-related CXCL.^328^

Type II IFNs are also involved in tumor immunity through JAK/STAT signaling. IFN-γ induces PD-L1 expression in TANs via the JAK/STAT pathway.^470–472^ In contrast to type I IFNs, IFN-γ activates JAK1 and JAK2, which subsequently recruit and phosphorylate STAT, forming STAT1 heterodimers.^452^ These STAT1 heterodimers bind to IRF1 to form a complex,^473^ which then translocates to the nucleus and binds to the IRF1 response element (IRE), modulating the transcription of PD-L1 genes and upregulating PD-L1 expression.^474^ Additionally, IFN-γ enhances the tumor-killing activity of PD-1 antibodies and induces the formation of NETs, promoting tumor cell apoptosis.^475^ Consequently, in neutrophils, IFN-γ may produce both anti-tumor effects and contribute to tumor immune evasion.

Type III IFNs have been shown to exert antifungal and antiviral effects predominantly in neutrophils through JAK/STAT signaling. Similarly to type I IFNs, IFN-λ activates JAK/STAT signaling but binds to the heterodimeric receptors IL-10RB and IFNLR1. Type I IFNs induce rapid, transient, and high-amplitude expression of ISG, while IFN-λ mediates long-term ISG expression at a lower amplitude.^476^ IFN-λ induces STAT1-dependent ROS production in neutrophils when exposed to *Aspergillus fumigatus*.^477^ In keratinocytes, IFN-λ signaling also inhibits CXCL9-mediated neutrophil recruitment to the skin, limiting the development of viral infectious dermatoses.^478^ In summary, IFN activation of JAK/STAT signaling in neutrophils is involved in inflammatory responses and tumor immunity.

Interleukins (ILs), a class of signaling proteins produced by immune cells, transmit signals between cells and mediate multiple immune effects.^479^ Several interleukin family members activate JAK/STAT signaling to regulate immune responses,^452^ with IL-6 thought to be involved in tumor immunity.^480^ IL-6 is highly expressed in the tumor microenvironment and is a major mediator of inflammation. It directly stimulates the proliferation, survival, and invasion of tumor cells, and induces production of pro-angiogenic cytokines that promote tumorigenesis in both immune and non-immune cells in the tumor microenvironment.^481^ Here, this review focuses on summarizing how IL-6 activates JAK/STAT signaling in neutrophils involved in the tumor immune response.

IL-6 binds to the secreted form of IL-6R (sIL-6R), forming an IL-6-sIL-6R complex that interacts with the transmembrane protein IL-6 receptor subunit-β (gp130; also known as IL-6Rβ).^482^ The IL-6-IL-6R-gp130 complex induces JAK1 and TYK2 phosphorylation. Subsequently, the SH2 domain of STAT3 recognizes and binds to these phosphotyrosine docking sites, positioning STAT3 near the active JAK. JAK phosphorylates STAT3 at the Tyr705 site, leading to SH2 domain-mediated head-to-tail dimerization of STAT3 proteins.^483^ The STAT3 dimer is translocated to the nucleus through an importin-α-importin-β1-dependent mechanism,^484^ where it binds to response elements in the promoters of target genes, inducing their transcription.^485^ It has been shown that hepatocellular carcinoma-associated fibroblast-derived IL-6 enhances the expression of PD-L1 in neutrophils *via* JAK/STAT3 signaling, impairing T cell function and promoting tumor immune evasion.^211^ Long-chain non-coding RNA HOTTIP enhances the immune escape of ovarian cancer cells by upregulating IL-6 expression in neutrophils, thereby enhancing PD-L1 expression.^486^ Moreover, oncostatin M (OSM), a member of the IL-6 family, promotes cancer cell proliferation through activation of JAK/STAT3 signaling.^487^ OSM is secreted primarily by T lymphocytes, neutrophils, and macrophages, and was originally introduced as an anticancer agent. However, due to its activation of multiple signaling pathways, OSM exhibits bidirectional effects in cancer.^488^ For example, when exposed to GM-CSF produced by breast cancer cells, neutrophils release significant levels of OSM, which induces VEGF expression by activating the JAK/STAT pathway in cancer cells.^489^ Tumor-associated neutrophil and macrophage interactions produce higher levels of OSM, activating STAT3 signaling in intrahepatic cholangiocarcinoma cells to promote tumor progression.^490^ Therefore, based on current studies, tumor cells appear to manipulate neutrophils in the tumor microenvironment to aid in immune evasion. Most tumor cells can produce IL-6, which promotes increased PD-L1 expression in neutrophils by activating JAK/STAT3 signal transduction, assisting the tumor in escaping immune surveillance.

## Neutrophils in human diseases

In recent years, neutrophils have been increasingly implicated in the development and progression of a wide range of diseases to varying degrees. They exhibit phenotypic and functional heterogeneity, revealing a level of complexity and sophistication far greater than previously recognized. In particular, neutrophils exhibit a dual effect in cancer, with diverse phenotypes that complicate the exploration of their functions in this context. Many diseases involving vascular inflammation are associated with the dysregulation of neutrophil homeostasis^468^; However, the pathophysiology of these disease can be varied. In this section, the impact of multiple pathophysiological mechanisms on neutrophil function will be discussed.

### Infectious diseases

#### Sepsis

**Fig. 7:**
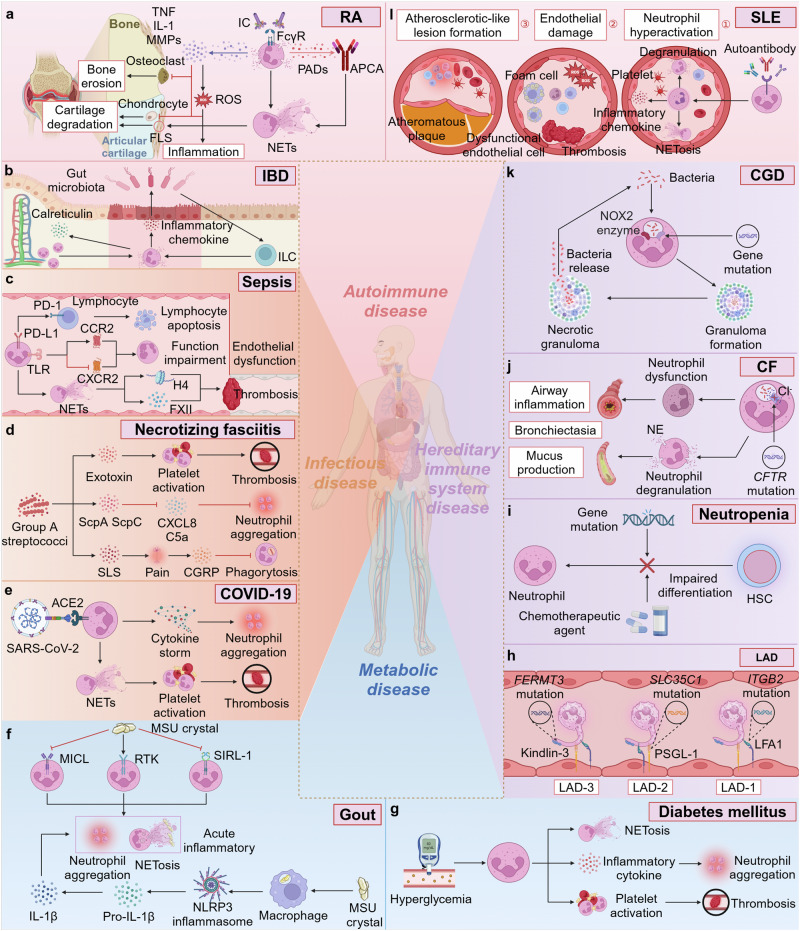
Neutrophils, a double-edged sword in the immune system, are involved in the development and progression of a wide range of diseases when dysfunctional. **a** Autoantibodies activate neutrophil pathogenic adaptive immunity. Neutrophil degranulation leads to joint damage and synovial inflammation. **b** Defects in the innate immune response of the gut lead to microflora imbalances and the hyperactivation of neutrophils, causing chronic inflammation and tissue damage. **c** Neutrophil dysfunction leads to vascular endothelial dysfunction, affecting microcirculatory blood flow, which causes inadequate tissue perfusion and triggers multi-organ failure. **d** *Streptococcus* induces platelet-neutrophil aggregation by releasing exotoxins, blocking capillaries and damaging the vascular endothelium. Neutrophils are unable to effectively clear the pathogen and allow it to spread, leading to persistent tissue necrosis. **e** Neutrophil activation by SARS-CoV-2 triggers excessive inflammatory responses and tissue damage. **f** The direct or indirect stimulation of neutrophils by MSU crystals induces acute attacks of gouty inflammatory responses. **g** Hyperglycemia stimulates neutrophil and platelet activation/adhesion, triggering a thrombotic inflammatory response, resulting in delayed or non-healing wounds. **h** Gene mutations affect the neutrophil adhesion cascade response, leading to defects in neutrophil adhesion, transport, and killing, inducing invasive gingivitis. **i** Gene mutations or chemotherapeutic drugs cause bone marrow hematopoiesis to stop maturation at the level of promyelocytes, decreasing neutrophil counts and increasing the risk of infection. **j** Gene mutations affect the expression of the chloride and bicarbonate transporter proteins, leading to the ineffective clearance of pathogens by neutrophils and inducing chronic bacterial airway infections and inflammatory responses. Neutrophil degranulation releases NE into the extracellular environment, leading to the degradation of connective tissue in the lungs and promoting mucus production in the airways. **k** Gene mutations affect the expression of the NADPH oxidase complex, leading to the ineffective clearance of pathogens by neutrophils and inducing chronic granulomatous lesions in the respiratory tract. **l** Neutrophil activation by autoantibodies triggers vascular dysfunction and inflammatory responses, resulting in atherosclerosis. RA Rheumatoid arthritis, ACPA Anti-citrullinated protein antibody, PADs Peptidylarginine deiminases, IC Immune complex, FLS Fibroblast-like synoviocyte, SLE Systemic lupus erythematosus, LDN Low-density neutrophil, IBD Inflammatory bowel disease, ILC Innate lymphocyte cell, PD-L1 Programmed cell death ligand 1, FXII Factor XII, DIC Disseminated intravascular coagulation, SLS Streptococcal hemolysin S, CGRP Calcitonin gene-related peptide, DFU Diabetic foot ulcer, MSU Monosodium urate, MICL Myeloid inhibitory C-type lectin-like receptor, SIRL-1 Signal inhibitory receptor on leukocytes-1, NLRP3 NOD-like receptor thermal protein domain associated protein 3, CGD Chronic granulomatous disease, LAD Leukocyte adhesion deficiency, CF Cystic fibrosis, COVID-19 Coronavirus disease 2019, SARS-CoV-2, Severe acute respiratory syndrome coronavirus 2, ACE2 Angiotensin-converting enzyme 2. Figure created with BioRender.com

Sepsis is a persistent systemic inflammatory disease involving multi-organ failure due to a dysregulated immune response to infection.^491^ As the primary line of defense against invading pathogen invasion, neutrophils exert their effector functions through three major approaches: degranulation, phagocytosis, and the release of NETs.^492^ However, during sepsis, neutrophils with an extended lifespan and impaired migration are confined to the vasculature, generating overwhelming vascular inflammation through the release of pro-inflammatory cytokines, ROS, and NETs,^493^ which eventually results in endothelial dysfunction (Fig. 7c). One study proposed that impaired neutrophil migration in sepsis patients may be attributed to reduced nuclear flexibility.^494^ Sepsis enhances the expression of programmed cell death ligand 1 (PD-L1) on neutrophils, triggering lymphocyte apoptosis and causing immunosuppression.^495^ Sepsis can also activate TLR2 on neutrophils, leading to the downregulation of CXCR2 and impaired chemotaxis.^496^ TLR activation can also lead to the expression of CC receptor 2 (CCR2), which is not present in neutrophils under physiological conditions, causing the inappropriate infiltration of neutrophils into CC ligand 2 (CCL2)-producing distal organs, which can lead to further tissue damage in organs such as the lungs, liver, and kidneys.^497^ One of the hallmarks of sepsis is endothelial dysfunction. Endothelial dysfunction may lead to impaired vasoconstriction, affecting microcirculatory blood flow, exacerbating tissue perfusion deficits in septic patients, and ultimately resulting in multi-organ failure.^498^ NETs have a dual function in the vasculature: trapping pathogens to prevent their spread and mediating thrombosis.^499^ Data from intravital microscopy in septic mice indicated that collaborative interactions between histone H4 in NETs induced intravascular coagulation.^500^ NETs have been shown to promote the interaction between factor XII (FXII) and neutrophils and initiate the endogenous coagulation pathway.^501^ Thrombosis induced by NETs contributes to organ ischemic damage and disseminated intravascular coagulation (DIC).^502^ Moreover, neutrophils and NETs damage the glycocalyx of endothelial cells and increase endothelial permeability, which may further lead to dysregulated inflammation, impaired microcirculatory blood flow, inadequate tissue perfusion, and life-threatening organ failure.^503^ Neutrophils and NETs constitute a strong antimicrobial defense. In sepsis, excessive neutrophil activation and NET release shift the endothelium from an anti-inflammatory and anticoagulant phenotype to a pro-inflammatory and pro-coagulant phenotype. The clinical treatment of sepsis is currently based on antibiotic therapy, fluid resuscitation, and supportive care. Thus, inhibiting the release of NETs may be a potential therapeutic strategy for the treatment of sepsis.

#### Necrotizing fasciitis

Unlike sepsis, which involves a systemic response, necrotizing fasciitis is a rapidly progressing and aggressive bacterial infection confined to the subcutaneous tissue and fascia.^504^ Although both are infectious inflammatory diseases associated with neutrophils, they differ in their etiological mechanisms, extent of lesions, and therapeutic strategies. Among the pathogenic microorganisms that can cause necrotizing fasciitis, group A streptococci are the most common.^505^ After invading the subcutaneous tissue, the bacteria proliferate and release exotoxins, which induce platelet-leukocyte aggregation, block capillaries, and damage the vascular endothelium, leading to fluid leakage, tissue swelling, and erythema. The infection gradually spreads until deeper and more extensive tissues are infected, eventually occluding small veins and arteries, which leads to the ischemic necrosis of all tissue layers from the dermis to the fascia^506^ (Fig. 7d). Neutrophils are essential for eliminating *Streptococcus pyogenes* and preventing the spread of bacteria. However, the blockage of small arteries prevents a steady flow of neutrophils from reaching the site of infection. *S. pyogenes* also secretes the proteases ScpA and ScpC to degrade crucial chemokines, such as CXCL8 and C5a, thereby disrupting neutrophil recruitment to the site of infection.^507^ This is reflected in the observation of a thin gray “dishwater” fluid containing a small number of neutrophils during surgical exploration.^506^ Additionally, *S. pyogenes* has been shown to hijack the neuronal regulation of pain and the immune response to promote bacterial survival. Specifically, it secretes streptococcal hemolysin S (SLS), which directly stimulates injury receptor neurons and elicit pain at the site of infection. Subsequently, injury receptors release the neuropeptide calcitonin gene-related peptide (CGRP) into infected tissues, which inhibits neutrophil recruitment and the opsonophagocytic killing of *S. pyogenes*.^508^ This also clearly explains one of the main clinical manifestations of necrotizing fasciitis, which is that the pain does not correspond to the physical manifestations at an early stage.^509^ Necrotizing fasciitis is currently treated surgically. The timely removal of necrotic tissue contributes to preventing the spread of infection, which otherwise may lead to systemic infection and ultimately to multi-organ failure. Targeting the peripheral nervous system and blocking neuroimmune communication has also been proposed as a promising strategy for treating highly invasive bacterial infections.^508^

#### Coronavirus disease 2019

Coronavirus disease 2019 (COVID-19) is a highly contagious respiratory disease caused by severe acute respiratory syndrome coronavirus 2 (SARS-CoV-2) infection.^510^ The clinical presentation and prognosis of patients with SARS-CoV-2 infection varies widely, from asymptomatic or febrile symptoms in mild cases to fatal acute respiratory distress syndrome (ARDS) and multiple organ dysfunction syndrome (MODS) in severe cases,^511^ primarily driven by dysregulated immune responses. Cytokine storms due to uncontrolled activation of the innate immune system, release of DAMPs during tissue injury, and intravascular thrombosis have all been associated with the function and malfunction of neutrophils (Fig. 7e). Studies have revealed a significant increase in circulating and lung tissue neutrophil counts in patients with COVID-19, especially in severe or critical patients.^512–514^ SARS-CoV-2 directly infects and activates neutrophils by binding to the angiotensin-converting enzyme 2 (ACE2) receptor,^515^ which leads to the release of cytokines and chemokines that further recruit and activate neutrophils to the site of infection. Moreover, aging appears to be associated with a decline in neutrophil efficiency, as evidenced by impaired phagocytosis and oxidative burst, reduced cytokine production, and altered chemotaxis,^516^ which increases the susceptibility of older people to infections and inflammatory diseases. Additionally, a study reported a significant increase in the frequency of LDNs in the circulating blood of patients with COVID-19, which correlated with the severity of the disease, particularly in severe or critical patients. The subsets of LDNs associated with COVID-19 are immunosuppressive and may lead to impaired lymphocyte responses during acute COVID-19.^517^ LDNs are shown to be particularly prone to NETosis, which is closely associated with vascular obstruction and COVID-19-related ARDS.^518^ Initially, NETs were thought to be a response of neutrophils to the presence of bacteria. Interestingly, NETs have also been found to be associated with antiviral defense effects,^519^ which is demonstrated by evidence that the level of NETs in the peripheral blood and lung tissue of COVID-19 patients is elevated.^520^ NETs have been demonstrated to promote thrombosis in a platelet-dependent manner through mechanisms such as platelet adhesion and activation, binding of cells to fibrinogen and von Willebrand Factor (vWF), and direct activation of the coagulation cascade.^521^ Many types of coronavirus vaccines have now been developed and are widely available to the general public, such as mRNA-1273 (Moderna), BNT162b2 (Pfizer-BioNTech), Chadox1 NCOV-19 (AstraZeneca), and AD26.COV2.S (Janssen).^522,523^ Neutrophils are considered a potential therapeutic target for coronavirus pneumonia. Receptor antagonists or monoclonal antibodies targeting neutrophil-associated cytokines, such as IL-1R, IL-1β, and IL-6, could serve as valuable strategies for improving the clinical outcomes of COVID-19 treatment.^524,525^ Additionally, targeting NETs with recombinant human deoxyribonuclease (DNase) presents another potential therapeutic avenue.^464^ While further research is required, therapies directed at neutrophils hold promise as a viable approach for the effective treatment of COVID-19.

### Metabolic diseases

#### Gout

Gout, a metabolic disease caused by hyperuricemia (serum urate level >7 mg/l [420 μmol/l]), is characterized by self-limiting inflammation caused by the deposition of monosodium urate (MSU) crystals in joints or other extra-articular tissues. If uric acid levels remain poorly controlled, gout usually progresses to the formation of greyish-white urate deposits, also known as tophi, in the joints and soft tissues, with the first metatarsophalangeal joint being the most common site.^526^ Uric acid itself is an endogenous and ubiquitous metabolite that is not considered pro-inflammatory, and the formation of MSU crystals is required to trigger clinically observed inflammation^527^ (Fig. 7f). The uptake of MSU crystals by macrophages promotes the assembly and activation of NLRP3 inflammatory vesicles, subsequently resulting in the activation of a variety of pro-inflammatory cytokines, among which IL-1β is a crucial mediator of gouty inflammatory attacks.^528^ IL-1β release leads to vasodilation and the rapid recruitment of neutrophils to the site of crystal deposition, which triggers an acute inflammatory episode.^529^ Moreover, stimulation of the tyrosine phosphorylation pathway is the most characteristic signaling event induced by MSU crystals in human neutrophils. This process involves a variety of Src family tyrosine kinases, such as Lyn, Syk, and Tec.^530^ MSU crystals can also directly stimulate neutrophils to induce inflammation by downregulating the expression of two inhibitory receptors on the cell surface, including the myeloid inhibitory C-type lectin-like receptor (MICL) and the signal inhibitory receptor on leukocytes-1 (SIRL-1).^531^ One intriguing feature of acute gouty arthritis is its spontaneous resolution, typically within 7-10 days.^532^ Aggregated NETs are assumed to be a possible mechanism for the spontaneous resolution of gout. The aggregation of NETs promotes the resolution of gouty inflammation by accumulating MSU crystals, degrading cytokines and chemokines, and preventing neutrophil recruitment and activation.^533^ This indicates that the formation of tophi is a self-protective action of the organism. In fact, the formation of tophi is a pathological manifestation of the body’s failure to compensate for the metabolism of uric acid, and the deposition of MSU will continue to erode bone, causing joint destruction and affecting limb function. The role and markers of NETs in gout remain controversial, and further studies are needed to investigate the composition, function, and mechanisms of NETs induced by MSU crystals. Different types of anti-inflammatory drugs have long been used clinically for the treatment of acute exacerbations and the effective prevention of gout, including colchicine that acts on microtubule proteins, non-steroidal anti-inflammatory drugs (NSAIDs) that inhibit the inflammation of cyclooxygenase (COX) enzymes, and corticosteroids with anti-inflammatory properties.^534^ However, the above drugs have varying degrees of side effects and limitations.^535^ Surgical removal is currently the only treatment for patients who have already exhibited visible tophi, which will inevitably result in some tissue or bone destruction. Thus, improved gout treatments and efficient drug delivery designs are needed.

#### Diabetes mellitus

Diabetes mellitus, a chronic progressive disease involving impaired glycemic control and glycemic variability, can be etiopathologically classified into three categories: type 1 diabetes mellitus (T1DM) or insulin-dependent diabetes mellitus, type 2 diabetes mellitus (T2DM) or non-insulin-dependent diabetes mellitus, and special types of diabetes, including gestational diabetes mellitus (GDM) and pancreatic diabetes mellitus.^536^ T1DM is characterized by impaired insulin secretion due to the destruction of pancreatic β-cells by the autoimmune system. T2DM is characterized by insulin resistance due to the loss of insulin sensitivity in glucose-using peripheral tissues and insufficient insulin secretion by β-cells due to risk factors, such as obesity, an unhealthy diet, and exercise deficiency.^537^ The impact of neutrophils on diabetes is currently being studied more in the context of diabetic complications, including diabetic nephropathy, diabetic eye disease, and diabetic foot ulcers (DFUs). However, neutrophils are also directly involved in T2DM pathogenesis. Increased circulating neutrophil counts in patients with T2DM may be due to the multiple effects of insulin resistance and other bodily factors, including hypercholesterolemia, systemic inflammation, and malignant disease.^538^ In preclinical models, neutrophils could induce insulin resistance by releasing IL-1β and NE, which impaired insulin signaling and degraded insulin receptor substrate-1 (IRS-1), respectively.^539,540^ Proteinase 3 (PR-3), a protease produced by neutrophils, exhibits proteolytic activity toward insulin-like growth factor-I (IGF-I) and insulin-like growth factor-binding protein-3 (IGFBP-3).^541^ Interestingly, studies found that patients with T1DM, their non-diabetic autoantibody-positive first-degree relatives, and high-risk subjects before the onset of symptoms exhibited decreased circulating neutrophil counts. This decrease is not due to peripheral neutrophil death, impaired differentiation, or the presence of anti-neutrophil antibodies.^542,543^

Chronic vascular inflammation caused by neutrophil hyperactivation may lead to some diseases, and diabetic complications are no exception. In the case of DFUs, for example, although the external manifestation of DFUs is a non-healing wound, the essence of the pathogenesis is a vascular lesion. The primary etiology of DFUs is now widely recognized as diabetic neuropathy and vasculopathy caused by persistent hyperglycemia; bacterial infections further exacerbate the difficulty associated with treating DFUs.^544^ Initially, sensory neuropathy leads to the loss of protective sensation, motor neuropathy leads to foot deformities and biomechanical abnormalities, and autonomic neuropathy leads to viscoelastic changes in the skin, such as dry skin,^545^ which makes the foot more susceptible to unintentional injuries, with even friction when walking, uncomfortable shoes, and washing the feet in hot water increasing the likelihood of injury. Peripheral arterial disease is the predominant cause of non-healing diabetic wounds. In clinical practice, most patients with DFUs are found to exhibit varying degrees of occlusion of the posterior tibial, dorsalis pedis, and peroneal arteries. Reduced blood flow to the lower limbs results in insufficient nutrients in the circulation of the foot to supply the wound to complete the healing process, as well as insufficient removal of inflammatory factors and metabolic waste products from the bloodstream. The tissue around the wound shows a continuous chronic inflammatory state, and a vicious circle occurs as the occlusion rate of the arteries of the lower limbs gradually increases until they are completely occluded. Most patients initially exhibit a slight wound in the toe, which grows in extent until tendons and bones are exposed. Eventually, the wound becomes gangrenous due to ischemia, resulting in surgical amputation. The interaction between neutrophils and platelets is thought to be an important driver of thromboinflammation.^546^ Hyperglycemia was shown to trigger thromboinflammatory responses by increasing platelet activation/adhesion and promoting the release of neutrophil- and platelet-derived particles in human samples^547^ (Fig. 7g). Moreover, glucose levels have been reported to increase NETosis in isolated human neutrophils in a concentration-dependent manner.^548^ NETs can further amplify the inflammatory response and the formation of thrombosis, which was described in section 3. Clinical treatment of DFUs with incomplete arterial occlusion is currently based on wound debridement, negative pressure wound therapy (NPWT), and skin grafting or flap repair surgery.^549^ Vacuum sealing drainage (VSD), as a specific form of NPWT, has been widely used in clinical treatments. Other promising therapeutic strategies, including hyperbaric oxygen therapy, electrical stimulation therapy, platelet-rich plasma therapy, growth factor therapy, stem cell therapy, application of herbal extracts, and bioengineered skin substitutes, have made great strides in recent years.^550^ Recent studies have focused on the development of diabetic wound healing dressings, including hydrogels, nano-enzymes, and chemically synthesized materials.^551,552^

### Hereditary immune system diseases

#### Leukocyte adhesion deficiency syndrome

Leukocyte adhesion deficiency (LAD) syndrome, a group of hereditary immune system diseases, is caused by defects in leukocyte adhesion to the endothelium at one of the various stages of the adhesion cascade^553^ (Fig. 7h). LAD is currently classified into three types: type I is due to mutations in the ITGB2 gene encoding the integrin β2 subunit; type II is due to defects in the fucosylation of the protein CD15s on neutrophils, which impairs the low-affinity rolling step of neutrophil adhesion mediated by selectins on the endothelium; and type III is due to defective expression of the kindlin 3 protein that is responsible for the activation of integrin signal transduction from the inside out.^554^ Neutrophil integrin expression depends exclusively on CD18, the common subunit of all integrins encoded by the ITGB2 gene.^555^ Thus, defects in CD18 expression or function result in defects in any or all of the CD11 family of molecules, leading to defects in adhesion, transport, and killing. Disease severity in LAD-1 is also determined by CD18 expression.^556^ Patients with LAD inherited in an autosomal recessive (AR) manner frequently present with poor wound healing, delayed umbilical cord separation, neutrophilia, oral ulcers, and aggressive gingivitis with accelerated apical bone loss.^557^ HSCT has been successfully performed in several cases of LAD, with clinical symptom improvement.^558^

#### Neutropenia

Neutropenia can be categorized into congenital or acquired forms based on its etiology. Severe congenital neutropenia (SCN) represents a spectrum of inherited hematopoietic disorders characterized by the impaired differentiation of neutrophilic granulocytes. Since the identification of *ELANE* mutations as a genetic cause for SCN or cyclic neutropenia (CyN) in 1999, numerous other genes implicated in inherited neutropenias have been uncovered, including *CXCR4*, *HAX1*, *WAS*, *CSF3R*, *G6PC3*, *TAFAZZIN*, *STK4*, *SBDS*, *VPS45*, and *LCP1*.^559^ In the majority of patients, bone marrow analysis reveals a maturational arrest of myelopoiesis at the promyelocyte stage,^560^ leading to reduced neutrophil counts (Fig. 7i). This hematopoietic disruption predisposes affected individuals to a heightened risk of infections, including otitis media, gingivitis, skin infections, pneumonia, and sepsis, with susceptibility beginning in the neonatal period and persisting throughout life without adequate treatment.^561^ Acquired neutropenia is caused by external factors, including radiation infections, and drugs, the most common of which is chemotherapy-induced neutropenia. Chemotherapy inflicts direct damage on both tumor and hematopoietic cells while also exerting indirect harm to the bone marrow stroma and microcirculation.^562^ Because chemotherapy targets dividing cells, mature neutrophils are usually unaffected by chemotherapy drugs. However, most cells in the mitotic pool, such as promyelocytes, and the maturation pool, such as metamyelocytes, are strongly susceptible to almost all chemotherapeutic agents.^563^ The main current treatment strategy for neutropenia involves the administration of G-CSF and HSCT. G-CSF remains the treatment of choice for almost all patients with CyN or SCN. However, in cases where patients are unresponsive to G-CSF therapy or progress to acute myeloid leukemia (AML) or myelodysplastic syndrome (MDS), HSCT is the only available treatment option.^564^

#### Cystic fibrosis lung disease

Cystic fibrosis (CF) lung disease, an autosomal recessive disorder with mutations in the cystic fibrosis transmembrane conductance regulator (*CFTR*) gene encoding the chloride and bicarbonate transporter proteins, is characterized by neutrophilic inflammation, chronic bacterial airway infections, and the dilatation of bronchioles obstructed by mucus plugs^565^ (Fig. 7j). As primary response cells against infection, neutrophils confine pathogens to phagosomes through phagocytosis and release bioactive molecules. These bioactive molecules, particularly MPO, H_2_O_2_ produced by NADPH oxidase, and chloride anions shuttled from the cytoplasm into the phagosome, form the MPO-H_2_O_2_-Cl system that impairs microbial integrity and viability through synergistic interactions. Specifically, MPO utilizes H_2_O_2_ to oxidize chloridion to produce hypochlorous acid (HOCl), which is a strong oxidizing antimicrobial agent.^4^ However, the absence of any of the three elements of the MPO-H_2_O_2_-Cl system, namely the absence of MPO (MPO deficiency), H_2_O_2_ (CGD), or Cl (CF), impairs normal host defense and inflammation resolution. *CFTR* mutations impair normal chloridion influx into neutrophil phagosomes and disrupt the activity of the MPO-H2O2-Cl system, ultimately leading to defective neutrophil function.^566^ Moreover, neutrophil degranulation releases NE into the extracellular environment, leading to the degradation of connective tissue in the lungs and promoting mucus production in the airways.^567^ The activity of soluble NE in the bronchoalveolar lavage fluid of infants with CF can be a reliable predictor of bronchiectasis.^568^ The presence of MPO in bronchoalveolar lavage fluid in CF patients may associated with the development of bronchiectasis.^569^ A variety of soluble inflammatory mediators were detected in bronchoalveolar lavage fluid in CF; CXCL8 was most prominently expressed at two orders of magnitude higher than in basal release.^570^ These pro-inflammatory cytokines recruit more functionally deficient neutrophils through different signaling pathways to maintain chronic inflammation in the airways of CF patients. In patients with CF, NETs have a deleterious effect on inflammation and lung destruction rather than exerting their antimicrobial capacity.^565^ Extracellular DNA concentrations have been shown to correlate with lung neutrophil concentrations, which can be used to measure lung inflammation and disease severity.^571^ The clinical treatment of CF is currently based on the appliance of antibiotics, bronchodilators, and expectorants, supplemented by airway clearance techniques and encouraging patients to exercise to facilitate the expulsion of respiratory secretions.^572–574^ Following the discovery of the CFTR gene in 1989 and the subsequent elucidation of the resulting various CFTR protein abnormalities, the treatment of CF has entered a new era of protein-targeted therapies.^575^ Several CFTR modulators have been developed in recent years to correct and enhance the function of mutant proteins, including CFTR enhancers and correctors.^576,577^ Although managing long-term inflammation remains a challenge, the development of CFTR-oriented therapies is a potential direction in CF treatment.

#### Chronic granulomatous disease

Chronic granulomatous disease (CGD), a recessive primary immunodeficiency disease (PID), is caused by mutations in the X-linked or autosomal genes encoding proteins that make up or are involved in the assembly of the phagocytic NADPH oxidase complex^578^ (Fig. 7k). During the neutrophil oxidative burst, ROS are produced in large quantities to contribute to the killing of specific microorganisms. Due to a defect in the production of ROS or superoxide by phagocytes, patients with CGD experience recurrent aggressive infections with specific bacteria and fungi, which induce granulomatous lesions in the respiratory and gastrointestinal tracts.^579^ The formation of NETs also appears to depend on ROS, and thus, in patients with CGD, defects in NETs contribute to the impact of microbial killing.^580^ This was demonstrated by the fact that neutrophils showed significant improvement in microbial killing when treated with tamoxifen, which induces NET formation in a ROS-independent manner.^581^ Most treatments for CGD are aimed at preventing and treating infections and inflammatory complications. Currently, hematopoietic stem cell transplantation (HSCT) is the available treatment for CGD.^582^ Recent research on gene therapy and enzyme replacement therapy has also progressed significantly, revealing promising strategies for CGD treatment.

### Autoimmune diseases

#### Systemic lupus erythematosus

Systemic lupus erythematosus (SLE), a prototypical autoimmune connective tissue disease, is characterized by the production of a wide range of autoantibodies, including anti-nuclear antibodies (ANAs), anti-double stranded DNA (dsDNA), anti-Sm/RNP, and anti-Ro/La, as well as complement depletion.^583^ Autoimmune-related inflammation leading to vascular injury and subsequent cardiovascular disease is a major cause of morbidity and mortality in SLE. Patients with SLE exhibit extensive vascular dysfunction, with the most characterized vascular disease being atherosclerosis of the large vessels^584^ (Fig. 7i). Neutrophils in SLE exhibit a pronounced interferon-1 (IFN-1) signature and are highly primed for enhanced NET formation.^468^ IFN-1 drives vascular damage, disrupts vascular repair processes, and fosters immune thrombosis and foam cell formation through its detrimental effects on endothelial cells.^585^ Neutrophils activated by ICs containing anti-RNP antibodies exhibit an enhanced propensity to release NETs, potentially serving as an additional source of immunogenic nucleic acids to maintain inflammation.^586^ NETs loaded with immunostimulatory and cytotoxic molecules in neutrophil granules can similarly damage endothelial cells. SLE neutrophils also exhibit enhanced ROS synthesis and secrete inflammatory cytokines.^587^ Moreover, a subpopulation of neutrophils known as low-density neutrophils (LDNs) are particularly potent mediators of vascular injury in SLE. LDNs may arise from the activation and degranulation of mature normal-density neutrophils (NDNs) and the release of immature neutrophils from the bone marrow. Functionally, LDNs can be further divided into immunosuppressive LDNs, also known as granulocyte myeloid-derived suppressor cells (G-MDSCs), and pro-inflammatory LDNs, also known as low-density granulocytes (LDGs).^588^ LDGs exhibit an increased ability to form NETs and release high levels of IFN-1 and inflammatory cytokines compared to normal neutrophils. LDGs are shown to be directly cytotoxic to endothelial cells, which damages these cells.^589^ Based on these findings, the recently proposed three-stage model of neutrophil-driven vascular inflammation may offer a mechanistic basis for cardiovascular complications and atherosclerosis associated with rheumatic diseases. The initial stage is characterized by immune-mediated activation of both neutrophils and endothelial cells. The second stage shows progression from initial endothelial damage to persistent damage. The final stage comprises the establishment of pro-inflammatory positive feedback loops, driving chronic vascular inflammation that can culminate in endothelial cell rupture and irreversible tissue damage.^590^ Like in RA, mitigating excessive inflammation through non-specific immunosuppressive therapies remains a cornerstone treatment for SLE. Emerging biologics and small-molecule inhibitors targeting neutrophils offer a promising therapeutic avenue for managing these diseases in the future.

#### Rheumatoid arthritis

Rheumatoid arthritis (RA), one of the most common systemic autoimmune diseases, is characterized by chronic progressive inflammation of the synovial joints. Autoantibodies and immune cells infiltrate and accumulate in the synovial cavity, which causes arthralgia and bone destruction, eventually resulting in joint deformity.^591^ Proteomic analysis has shown that neutrophils are the most abundant cell type in the inflamed synovial fluid in RA.^592^ Neutrophils are also present in the synovial fluid of healthy individuals. However, due to chronic inflammation in the joint space of RA patients, neutrophil counts in the synovial fluid increase and neutrophil components cause damage to joint tissues (Fig. 7a). The local inflammatory environment leads to fundamental changes in the biology of resident fibroblast-like synoviocytes (FLSs), which promote the formation of a proliferative, inflammatory, and invasive synovium, termed pannus.^593^ The onset of RA is preceded by a phase of quiescent autoimmunity termed preclinical RA (pre-RA). This period is characterized by the production of antibodies against self-proteins, particularly anti-citrullinated protein antibodies (ACPAs), which are directed against citrullinated proteins. Protein citrullination is carried out by PADs, particularly PAD2 and PAD4. Both PAD2 and PAD4 have been identified as RA-risk SNPs, suggesting that these enzymes may be involved in the disruption of immune tolerance.^594^ Neutrophils are shown to be an important source of PAD2 and PAD4.^595^ Moreover, the inflammatory environment in the synovium as well as autoantibodies, including ACPAs and rheumatoid factors (RFs), appear to be particularly favorable for the formation of NETs.^596^ NETs may interact with FLSs to activate pathogenic adaptive immunity, which may ultimately result in immune dysregulation, amplified inflammatory responses, and cartilage damage.^597^ Additionally, ICs responsible for complement activation are present in the serum and synovial fluid of RA patients, which can induce neutrophil degranulation by signaling through FcγRs.^598^ Various serine proteases in neutrophil granules can contribute to joint damage and synovial inflammation. Neutrophil elastase, histone G, and proteinase-3 activate pro-inflammatory cytokines, cleave adhesion molecules, and regulate chemokine function.^599,600^ MMP-8 and MMP-9 promote the degradation of type 2 collagen in articular cartilage.^601^ MPO also forms ROS byproducts, which increase oxidative damage in the joint.^602^ Overall, the abnormal release of neutrophil granules, which are supposed to have an immunoprotective function, can cause inflammation and tissue damage in synovial joints in RA. The clinical treatment of RA is currently based on hormonal agents, pain medications, and physical therapy. Neutrophils are also shown to have anti-inflammatory properties,^603^ which may provide a new therapeutic strategy for patients with RA.

#### Inflammatory bowel disease

Inflammatory bowel disease (IBD), including Crohn’s disease and ulcerative colitis, is essentially an inflammatory disease; however, it is not caused by the spontaneous overactivation of inflammatory pathways.^604^ Rather, the etiology of IBD may be associated with defects in the innate immune response. Defects in the intestinal epithelial barrier lead to impaired homeostasis of the intestinal microflora, as well as activation of the adaptive immune system, which promotes a secondary inflammatory response, leading to chronic inflammation and tissue damage.^605^ Neutrophils are crucial participants in intestinal innate immunity, with both deleterious and protective effects. As phagocytes, neutrophils regulate the clearance of microbiota. However, during chronic inflammation, the infiltration of large numbers of dysfunctional neutrophils alters the intestinal environment. This alteration is primarily mediated through the release of ROS and proteins contained in neutrophil granules. Such changes provide a selective advantage to parthenogenetic anaerobic bacteria, promoting the expansion of Enterobacteriaceae in the inflammatory environment of the human gut.^606^ Neutrophil infiltration of the intestinal mucosa is a hallmark of active IBD^607^ (Fig. 7b). The main fecal biomarkers of IBD activity are neutrophil granule proteins, such as calreticulin.^608^ Moreover, the detection of anti-granulocyte macrophage colony-stimulating factor autoantibodies (aGMAbs; also known as anti-GM-CSF antibodies) was demonstrated to contribute to predicting complicated ileal Crohn’s disease prior to diagnosis. aGMAbs affected the relative abundance of neutrophils in bone marrow and promoted the production of group 1 innate lymphocytes (ILCs), leading to perturbations in intestinal immune homeostasis.^609^ This suggests that neutrophils are at the center of IBD development.^610^ The human genome contains more than 350 risk loci associated with drivers of IBD. Several of these IBD susceptibility genes are involved in neutrophil functions essential for microbial defense, including *NOD2*, *NCF4*, *LRRK2*, and *CARD9*.^611^ The administration of yeast-produced GM-CSF has been found to improve some symptoms of Crohn’s disease and ileitis in mice.^612^ These results indicate that targeting neutrophil-microbiota crosstalk presents a promising therapeutic strategy for IBD. Because of the duality and complexity of neutrophil function in this disease, more research is needed to elucidate neutrophil function in host-microbiota interactions under inflammatory and homeostatic conditions.

In humans, a complete absence, a significant reduction in number, and dysfunction of neutrophils lead to severe immunodeficiency and even death. In contrast, the excessive or aberrant activation of neutrophils results in significant damage to tissues and organs.^43^ Factors contributing to the dysregulation of neutrophil homeostasis can be categorized into external factors, such as pathogens, injury, and pharmacological stimuli, as well as internal factors, such as autoantibodies, genetic mutations, and cancer. Due to their unique diversity and heterogeneity, the role of neutrophils in the tumor microenvironment appears to be more complex than in the inflammatory microenvironment. Neutrophils may be susceptible to being hijacked by the tumor microenvironment to adjust their function to promote tumor growth.^86^ Thus, exploring the diversity and function of neutrophils in human diseases contributes to better understanding the onset, progression, and prognosis of diseases, aiding the development of more comprehensive and effective treatment strategies.

## Strategies for targeting neutrophils in human diseases

Considering the dual effect of neutrophils in disease, research over the past two decades has continuously evolved to develop therapeutic strategies targeting neutrophils. These include anti-infective agents, anti-inflammatory drugs, anti-tumor therapies, drug delivery systems utilizing neutrophils, and clinical tests related to neutrophil function. Here, approved neutrophil-targeted drugs and recent advancements in therapeutic strategies involving neutrophils will be emphasized.

### Anti-infective therapy

As effector cells of the innate immune system, neutrophils are the first line of defense against pathogenic infections. They recognize PAMPs *via* PRRs, triggering intracellular signal transduction and subsequent phagosome formation. Pathogens engulfed within phagosomes are eliminated through oxygen-dependent or oxygen-independent mechanisms.^50^ However, some pathogens evade clearance by forming antiphagocytic biofilms, producing cytotoxins to target neutrophils, and employing camouflage tactics to evade immune surveillance. Anti-infective therapy targeting neutrophils focuses on two approaches: enhancing neutrophil activity and increasing neutrophil abundance. Conditions such as congenital neutropenia, severe infections, chemotherapy, and myelosuppressive disorders can cause a significant reduction in neutrophil counts, underscoring the critical need for strategies to increase neutrophil abundance in these contexts.

#### Enhancing neutrophil activity

Anti-microbial drugs are the primary choice for anti-infection therapy. In addition to targeting pathogens, some drugs also enhance neutrophil activity, exerting a synergistic effect on inhibiting pathogens.^613^ For instance, fosfomycin has been demonstrated to increase ROS production and enhance the oxidative burst process in neutrophils.^614^ Erythromycin has been shown to stimulate neutrophil phagocytosis.^615^ Additionally, several plant-derived compounds, including gingerol, arecoline, and magnoflorine, enhance neutrophil phagocytosis.^616^ Moreover, stimulating NET production during infection contributes to the elimination of pathogens and infection control. Amoxicillin,^617^ clarithromycin,^618^ and enrofloxacin^619^ have been found to promote NETosis in neutrophils. Synthetic or natural experimental compounds, such as phorbol myristate acetate (PKC agonist), A23187 (Ca^2+^-ionophore), paraquat (redox mediator), and FPR agonists, also stimulate ROS production in neutrophils through NOX2-dependent regulation.^620^ Enhancing neutrophil activity proves effective in early-stage infection to inhibit pathogen invasion. However, it can lead to overactivation of neutrophils, resulting in chronic persistent inflammation, as seen in sepsis and COVID-19. These severe infections often culminate in multi-organ failure due to systemic inflammatory response syndrome, highlighting the dangers of neutrophil overactivation. Therefore, while enhancing neutrophil activity contributes to the prevention of pathogen invasion, caution is necessary to avoid risks associated with neutrophil hyperactivity.

#### Increasing neutrophil abundance

Congenital or acquired neutropenia compromises immune system function, significantly increasing the susceptibility to infection. Even common pathogens can lead to life-threatening infections under these conditions. Thus, maintaining an adequate number of circulating neutrophils is essential for infection treatment (Table 1). Because of its potent ability to stimulate neutrophil production, G-CSF was one of the first cytokines approved to enter clinical trials. The development of recombinant granulocyte colony-stimulating factor (rHuG-CSF), a hematopoietic growth factor that acts selectively on the neutrophil spectrum, has been a tremendous boon to patients with neutropenia and infections caused by cancer chemotherapy.^621^ Initially, two forms of rHuG-CSF were successively approved by the FDA for use in the clinic: filgrastim and lenograstim.^622^ Lenograstim, derived from Chinese hamster ovary cells, is glycosylated, while filgrastim, produced in *Escherichia coli*, is nonglycosylated.^38^ Subsequently, more rHuG-CSF variants with different structural forms were developed, including pegylated forms (pegfilgrastim and lipegfilgrastim) and N-terminus mutated forms (nartograstim).^559^ The polyethylene glycolized form of rHuG-CSF extends the half-life of G-CSF, thereby reducing the number of injections given to patients.^623–625^ Filgrastim was widely used against neutropenia-related diseases in the 15 years following its development and was gradually refined and approved for other indications. However, with the end of patent protection for filgrastim in 2006, other pharmaceutical companies have produced biosimilars that are similar to filgrastim and are gradually refining their efficacy, reducing side effects, and lowering costs. These biosimilars have also gained FDA approval for marketing in recent years.^622,626,627^ Telpegfilgrastim, a novel PEGylate recombinant human G-CSF (YPEG-rhG-CSF) developed independently in China, has received marketing approval and has been included in the medical insurance catalog. Unlike conventional PEG modifications with a 20 kD straight chain structure, telpegfilgrastim features a PEG modification with a 40 kD Y-type structure, resulting in an extended half-life and increased potency. A phase III clinical trial (NCT04466137) has demonstrated the efficacy and safety of YPEG-rhG-CSF in treating malignancies with chemotherapy.^628^

**Table 1:** Current therapeutic drugs targeting neutrophil abundance

Therapeutic Category	Drug	Target	FDA Approval	Indication	References
rHuG-CSF	Filgrastim	CSFR3	Yes	·Neutropenia ·Aplastic anemia ·Myelodysplastic syndromes	^630,631,641,642^
	Lenograstim	CSFR3	Yes	·Neutropenia ·Autologous stem cell transplant (ASCT)	^622,808,809^
	Pegfilgrastim	CSFR3	Yes	·Neutropenia	^563^
	Lipegfilgrastim	CSFR3	Yes	·Neutropenia	^810^
	Telpegfilgrastim	CSFR3	No; Approved in China.	·Neutropenia	^628^
CXCR4 antagonist	AMD3100	CXCR4	Yes	·Non-Hodgkin ·lymphoma (NHL) ·Multiple myeloma (MM)	^651,660^
	Motixafortide	CXCR4	Yes	·Autologous stem cell transplant (ASCT) ·Pancreatic cancer ·Acute myeloid leukemia	^672^
	Mavorixafor	CXCR4	Yes	·WHIM syndrome	^674^

Filgrastim was initially developed and tested clinically in 1987 and received FDA approval in 1991 for cancer patients undergoing chemotherapy.^39,40,629^ Several phase I/II studies have demonstrated that administering filgrastim post-chemotherapy significantly reduces the severity of neutropenia.^41,630,631^ Both preclinical and clinical studies confirmed that filgrastim caused a specific and rapid increase in neutrophil counts.^632,633^ This swift increase is attributed to a shortened maturation period, an enhanced number of cell divisions, and the accelerated release of neutrophils into peripheral circulation.^634^ Early studies have shown that, similar to endogenous G-CSF, filgrastim selectively stimulates the proliferation and differentiation of neutrophil precursor cells through binding to its specific cell surface receptor.^635^ In addition to being a therapeutic agent, filgrastim can be administered as a prophylactic agent prior to chemotherapy. Current guidelines from the American Society of Clinical Oncology, the European Organization for Research and Treatment of Cancer (EORTC), and the National Comprehensive Cancer Network advocate for the use of CSFs for primary prevention in adult cancer patients undergoing cytotoxic chemotherapy when the overall risk of febrile neutropenia (FN) is 20% or higher.^636,637^ Patients receiving prophylactic filgrastim experienced significantly fewer dose reductions and delays in cytotoxic chemotherapy compared to those given a placebo or no supportive care.^638,639^ Along with its potency, the side effects of filgrastim were also recognized in clinical practice. Initially, across all Phase II and Phase III trials involving patients undergoing myelosuppressive chemotherapy, musculoskeletal pain emerged as the sole consistently reported adverse event directly associated with filgrastim administration.^640^ In some countries, it has also received approval for a range of other indications, including aplastic anemia,^641^ myelodysplastic syndromes,^642^ acute leukemia,^643^ and severe chronic neutropenia.^644^ The mobilization of peripheral blood stem cells (PBSCs) can be effectively achieved with G-CSF, which has been demonstrated to enhance and accelerate engraftment following HSCT.^645^ Interestingly, a study found that G-CSF treatment heightened the risk of acute graft-versus-host disease (GVHD) in bone marrow transplant (BMT) recipients but not in PBSC recipients.^646^ The use of G-CSF-mobilized PBSCs has fundamentally transformed stem cell transplantation, rendering the process more efficient, accessible, and clinically applicable.

Neutrophils undergo homing to the bone marrow through activation of CXCR4/CXCL12 signal transduction. Thus, targeted inhibition of CXCR4 promotes neutrophil release from bone marrow, increasing neutrophil abundance in the peripheral circulation. Initially, AMD3100 (Plerixafor), a CXCR4 antagonist, was identified as an inhibitor of viral entry by interfering with an early, post-envelope binding step in the replicative cycle of human immunodeficiency virus 1 (HIV-1).^647^ However, it was not approved for HIV-1 treatment, primarily due to its inability to inhibit macrophage-tropic HIV-1 strains, associated cardiac disturbances, and poor oral bioavailability.^648–650^ Interestingly, during pharmacokinetic studies conducted in HIV-1 clinical trials, an unexpected observation was made: even at low doses, AMD3100 induced a rapid mobilization of CD34^+^ HSPCs into the peripheral blood.^651–654^ Further studies revealed that AMD3100 works synergistically with rHuG-CSF to enhance the mobilization of CD34^+^ cells from the bone marrow into the peripheral circulation,^654–656^ making it suitable for transplantation. Ultimately, the FDA approved the combination of AMD3100 and rHuG-CSF for the mobilization of HPSCs for autologous transplantation in patients with multiple myeloma (MM) and non-Hodgkin lymphoma (NHL).^657^ Beyond its established use in mobilizing hematopoietic stem cells for bone marrow transplantation, AMD3100 has also been explored as a therapeutic option in patients with warts, hypogammaglobulinemia, infections, and myelokathexis (WHIM) syndrome. A phase III clinical trial (NCT02231879) revealed that long-term administration of AMD3100 can reverse neutropenia and significantly lowers the incidence of infections in affected patients.^658^ Investigations in murine models have shown that AMD3100 mobilizes neutrophils from the bone marrow into the blood and spleen. Importantly, AMD3100 has not been found to alter neutrophil kinetics at the lung margins, suggesting that it is unlikely to impair respiratory host defenses.^659^

The study also found that a new strategy of adding AMD3100 to existing chemotherapy, radiotherapy, or immunotherapy could significantly improve treatment outcomes for a variety of cancers.^660^ AMD3100, when combined with other anticancer agents, has demonstrated therapeutic potential across a broad spectrum of cancers, including cervical cancer,^661^ pancreatic cancer,^662^ ovarian cancer,^663^ mesothelioma,^664^ prostate cancer,^665^ hepatocellular carcinoma,^666^ breast cancer,^667^ and melanoma.^668^ By directly targeting the CXCR4/CXCL12 axis, AMD3100 inhibits tumor growth and metastasis, while also functioning as a potent immunomodulator, preventing the establishment of an immunosuppressive intratumoral microenvironment.^669–671^

Motixafortide, a novel CXCR4 inhibitor, induces rapid and sustained mobilization of HSPCs.^672^ FDA approval has been granted for its use in combination with G-CSF for the collection of hematopoietic stem cells suitable for subsequent autologous transplantation in patients with MM. It holds Orphan Drug Designation for the treatment of pancreatic cancer in the EU and USA, as well as for acute myeloid leukemia in the USA.^673^ A phase I clinical trial (NCT05618301) is currently evaluating the safety and efficacy of motixafortide in combination with natalizumab for mobilizing CD34^+^ hematopoietic stem cells in patients with sickle cell disease.

Mavorixafor, another small molecule CXCR4 antagonist, has gained FDA approval for the treatment of WHIM syndrome.^674^ WHIM syndrome typically results from an autosomal dominant pathogenic variant in the C-terminal end of the CXCR4 gene, resulting in reduced internalization of CXCR4 and hyperactive downstream signaling. This disrupts neutrophil migration in response to the CXCL12 concentration gradient, preventing their movement into peripheral circulation to exert immune defense. Neutrophils consequently overmature in the bone marrow, impairing T cell function and development.^675^ A phase III clinical trial (NCT03995108) demonstrated that mavorixafor reduces infection frequency, severity, duration, and the need for antibiotic applications in patients with WHIM syndrome.^676^ Another phase III clinical trial (NCT06056297) is assessing the safety and tolerability of mavorixafor in patients with congenital or acquired primary autoimmune and idiopathic chronic neutropenia. Mavorixafor may emerge as a potent treatment for neutropenia following rHuG-CSF in the near future.

### Anti-inflammatory therapy

Moderate and accurate neutrophil recruitment is critical for pathogen defense, maintaining homeostasis, modulating inflammatory responses, promoting wound healing, and facilitating tissue repair. Neutrophils mediate inflammatory responses through degranulation and secretion of pro-inflammatory cytokines. Inflammation acts as a double-edged sword in the immune response: moderate inflammation aids in pathogen elimination, limits damage, and promotes tissue repair, while excessive inflammation leads to immune dysregulation, spread of infection, tissue injury, and organ dysfunction.^43^ Neutrophil dysfunction underlies many inflammation-related diseases, making therapeutic strategies targeting neutrophils essential in anti-inflammatory treatments. Two routes of neutrophil-related anti-inflammatory therapy are available: inhibiting signal transduction of the inflammatory response in neutrophils and modulating inflammatory factors or contents released by neutrophils. These mechanisms underlie the anti-inflammatory effects of numerous drugs (Table 2). Here, currently market-approved commercially available drugs and preclinical candidates that exert anti-inflammatory effects through these pathways will be emphasized.

**Table 2:** Current therapeutic drugs targeting neutrophil-associated anti-inflammatory strategies

Therapeutic Category	Drug	Target	FDA Approval	Indication	References
Anti-IL-1R monoclonal antibody	Anakinra	IL-1R	Yes	·Rheumatoid arthritis (RA) ·Neonatal-onset multisystem inflammatory disease (NOMID) ·Cryopyrin-associated periodic syndromes (CAPS) ·Adult-onset Still disease (AOSD) ·Systemic juvenile idiopathic arthritis (sJIA) ·Schnitzler’s Syn ·Deficiency of IL1 receptor antagonist (DIRA) ·COVID-19	^677,678^
IL-1R trap fusion protein	Rilonacept	IL-1R	Yes	·Familial cold autoinflammatory syndrome (FCAS) ·Muckle-Wells syndrome (MWS) ·Deficiency of IL1 receptor antagonist (DIRA) ·Recurrent pericarditis	^677^
Anti-IL-6R monoclonal antibody	Tocilizumab	IL-6R	Yes	·Castleman’s disease ·Rheumatoid arthritis (RA) ·Systemic arthritis and polyarticular juvenile idiopathic arthritis ·Giant cell arteritis ·COVID-19-associated systemic inflammatory response syndrome	^684^
	Sarilumab	IL-6R	Yes	·Rheumatoid arthritis (RA)	^687^
CXCR2 antagonist	Navarixin	CXCR1/CXCR2	No	·Psoriasis ·Asthma	^689^
JAK antagonist	Tofacitinib	JAK3 > JAK2 > JAK1	Yes	·Rheumatoid arthritis ·Ulcerative colitis ·Psoriatic arthritis ·Polyarticular juvenile idiopathic arthritis	^452^
	Baricitinib	JAK1 and JAK2, and moderate activity against TYK2	Yes	·Rheumatoid arthritis ·Alopecia areata ·COVID-19	^452,701,702^
	Ruxolitinib	JAK1 and JAK2 > JAK3 > TYK2	Yes	·Moderate and high-risk myelofibrosis (MF) ·Steroid-refractory acute graft-versus-host disease ·True erythrocytosis	^704,705^
	Upadacitinib	JAK1 > JAK2 and JAK3	Yes	·Moderate to severely active Crohn’s disease ·Moderate to severely active RA ·Active psoriatic arthritis ·Non-imaging axial spondyloarthritis	^706–708^
	Peficitinib	All JAK proteins	Approved in Japan	·Rheumatoid arthritis	^709^
	Fedratinib	JAK2	Yes	·Moderate or high risk of myelofibrosis	^710^
	Momelotinib	JAK1 and JAK2	Yes	·Moderate or high risk of myelofibrosis	^711^
C5aR1 antagonist	Avacopan	C5aR1	Yes	·ANCA-associated vasculitis	^718^
Anti- IL-1β monoclonal antibody	Canakinumab	IL-1β	Yes	·Familial cold autoinflammatory syndrome (FCAS) ·Muckle-Wells syndrome (MWS) ·Cryopyrin-associated periodic syndromes (CAPS) ·Systemic juvenile idiopathic arthritis (sJIA) ·Familial Mediterranean fever (FMF) ·Mevalonate kinase deficiency (MKD) ·TNF receptor-associated periodic syndrome (TRAPS) ·Adult-onset Still disease (AOSD)	^677^
Anti-IL-6 monoclonal antibody	Siltuximab	IL-6	Yes	·Castleman’s disease	^719^
Anti-IL-8 monoclonal antibody	Anti-human IL-8 monoclonal antibody cream	IL-8	Approved in China	·Psoriasis Eczema	^720^
Anti-TNF-α monoclonal antibody	Infliximab	TNF-α	Yes	·Inflammatory bowel disease (IBD) ·Rheumatoid arthritis (RA) ·Psoriasis ·Kawasaki disease	^722–724^
Anti-C5 monoclonal antibody	Eculizumab	C5	Yes	·Paroxysmal nocturnal hemoglobinuria ·Atypical hemolytic uremic syndrome	^725^
	Vilobelimab	C5	Yes	·COVID-19	^727^
	Ravulizumab	C5	Yes	·Anti-AChR antibody-positive myasthenia gravis (gMG) ·Atypical hemolytic uremic syndrome (aHUS) ·Paroxysmal nocturnal hemoglobinuria (PNH)	^730–732^
Neutrophil elastase (NE) inhibitor	Sivelestat	Neutrophil elastase (NE)	Approved in Japan	·COVID-19 ·COPD ·Acute lung injury/acute respiratory distress syndrome (ALI/ARDS) ·Chronic liver diseases and cirrhosis	^733–735^
Myeloperoxidase (MPO) inhibitor	AZD5904	Myeloperoxidase (MPO)	No	·COPD ·Multiple sclerosis (MS)	^736,737^
Recombinant human deoxyribonuclease (DNase)	Dornase Alfa	DNA Neutrophil extracellular traps (NETs)	Yes	·COVID-19 ·Cystic fibrosis (CF) ·COPD	^738–740^

#### Drugs inhibiting signal transduction of the inflammatory response in neutrophils

The interleukin family, which includes IL-1, IL-6, and IL-8, comprises major pro-inflammatory cytokines. Interleukins bind to interleukin receptors on neutrophils, initiating intracellular signal transduction cascades that regulate gene expression, promote the release of inflammatory mediators, and modulate immune cell activity. Consequently, strategies aimed at blocking these receptor-ligand interactions or inhibiting intracellular signal transduction constitute a promising avenue for anti-inflammatory therapy.

Anakinra, an anti-IL-1R monoclonal antibody, inhibits IL-1-mediated inflammatory responses by binding to the IL-1 receptor. Anakinra was first approved by the FDA for the treatment of RA and has since been approved in several countries for neonatal-onset multisystem inflammatory disease (NOMID), cryopyrin-associated periodic syndromes (CAPS), adult-onset Still disease (AOSD), systemic juvenile idiopathic arthritis (sJIA), Schnitzler’s syndrome, and deficiency of IL1 receptor antagonist (DIRA).^677^ During the COVID-19 pandemic, anakinra was also approved by the European Medicines Agency (EMA) for the treatment of COVID-19 pneumonia.^678^ Additionally, anakinra showed therapeutic efficacy in patients with febrile infection-related epilepsy syndrome (FIRES).^679^ An international retrospective cohort study has confirmed the safety and potential efficacy of anakinra as an immunomodulator for FIRES.^680^ A phase II clinical trial (NCT03925194) is currently assessing anakinra’s safety and efficacy for treating CF.

In contrast, rilonacept is an IL-1R trap fusion protein that binds IL-1α and IL-1β to prevent their binding to IL-1R.^681^ Rilonacept was first approved by the FDA for the treatment of familial cold autoinflammatory syndrome (FCAS) and has since been approved in several countries for the treatment of Muckle-Wells syndrome (MWS), DIRA, and recurrent pericarditis.^677^ A phase III clinical trial (NCT03737110) has established that long-term use of rilonacept reduces the risk of recurrence of persistent pericarditis.^682^

Tocilizumab, a monoclonal antibody targeting the IL-6 receptor, suppresses IL-6-mediated inflammatory pathways by binding to its receptor. It was first approved in Japan for the treatment of Castleman’s disease and has since been approved in several countries for the management of RA, systemic arthritis, polyarticular juvenile idiopathic arthritis, and giant cell arteritis.^683^ During the COVID-19 pandemic, tocilizumab was also approved by the FDA for the treatment of COVID-19-associated systemic inflammatory response syndrome.^684^ Additionally, tocilizumab has demonstrated therapeutic efficacy in patients with neuromyelitis optica (NMO).^685^ A phase II/III clinical trial (NCT03350633) indicated that tocilizumab reduces the risk of relapse in patients with NMO and may offer safe and effective treatment following azathioprine administration.^686^ Another phase II clinical trial (NCT06033196) is evaluating the efficacy of tocilizumab for the treatment of lung transplant-induced inflammation and alloimmunization.

Similarly, sarilumab is a fully humanized anti-IL-6R monoclonal antibody approved for RA. It has also shown beneficial effects on survival in severe COVID-19 cases.^687^ A phase II clinical trial (NCT02991469) is currently evaluating sarilumab’s efficacy and safety of in treating children and adolescents with sJIA.

The blockade of IL-8 signaling accomplished by CXCR2 antagonists or monoclonal anti-IL-8 antibodies. Notably, some CXCR2 inhibitors act as dual antagonists against CXCR1. Their efficacy in inhibiting neutrophil recruitment and mitigating inflammatory damage has been demonstrated across various animal models. Navarixin, also known as MK-7123, is a non-competitive and orally active CXCR1 and CXCR2 antagonist. Navarixin inhibits IL-8 binding to CXCR1 and CXCR2, effectively reducing neutrophil chemotaxis to IL-8 and CXCL1.^688^ This compound has undergone evaluation in three phase II trials (NCT00684593, NCT00632502, NCT00688467) for the treatment of psoriasis and asthma, showing promise by reducing phlegm neutrophils in asthma patients.^689^ Moreover, it exhibits anti-angiogenic effects in tumors and accelerates tumor cell apoptosis, thereby suppressing colon cancer metastasis.^690^ Navarixin also demonstrates the inhibition of melanoma cell proliferation, chemotaxis, invasiveness, reduction of microvessel density, and tumor cell proliferation.^691^ Several CXCR1/CXCR2 antagonists are currently progressing through clinical development stages for various inflammatory diseases. However, the limited efficacy shown in clinical trials and the necessity for ensuring drug safety present important challenges. Delayed drug development may stem from the fact that CXCR2 is activated by multiple chemokines, at least two of which also activate CXCR1. Additionally, mechanistic insights into how CXCR2 signaling inhibition affects diverse cell types in clinical settings remain elusive.^692^

Most members of the pro-inflammatory interleukin family form complexes with binding receptors to regulate inflammation through the activation of JAK/STAT signal transduction pathways. Thus, blocking JAK/STAT signaling represents a potential therapeutic strategy for anti-inflammatory purposes. Tofacitinib, also known as Xeljanz or CP690,550, is the first JAK inhibitor to be approved for human use. Pharmacologically, tofacitinib inhibits JAK/STAT signaling by targeting JAK3.^693^ It has been approved for treating RA, ulcerative colitis, psoriatic arthritis, and polyarticular juvenile idiopathic arthritis.^452^ During the COVID-19 pandemic, tofacitinib was considered a candidate for managing severely affected patients.^694^ A phase III clinical trial (NCT04469114) demonstrated that tofacitinib reduces the risk of death or respiratory failure in hospitalized COVID-19 pneumonia patients.^695^ Additionally, it has shown efficacy in treating atopic dermatitis, alopecia areata, and ankylosing spondylitis.^696–698^ Common adverse events associated with tofacitinib include opportunistic infections, thromboembolism, gastrointestinal perforation, and herpes zoster.^699^ A phase II clinical trial (NCT04246372) is also evaluating the efficacy of tofacitinib in the treatment of immune dermatological disorders in Down syndrome. Given that tofacitinib inhibits numerous crucial cytokines and immune cells, its evaluation requires additional safety data to ensure its applicability for the therapy of other diseases.

Baricitinib, a structural analog of tofacitinib, mainly inhibits JAK1 and JAK2 and has moderate activity against TYK2.^700^ It is approved for RA, alopecia areata, and COVID-19.^452^ Baricitinib is the first JAK inhibitor approved by the FDA for the treatment of COVID-19 and the first systemic therapy endorsed for alopecia areata.^701,702^ Additionally, baricitinib exhibits anti-HIV activity in the central nervous system and has therapeutic potential for HIV-associated neurocognitive disorders.^703^ Ongoing research in a phase II clinical trial (NCT05452564) is assessing the efficacy of baricitinib in suppressing HIV in the central nervous system.

Several other JAK inhibitors are progressively gaining approval for diverse diseases. Ruxolitinib has been approved by the FDA for the treatment of moderate and high-risk myelofibrosis (MF), steroid-refractory acute graft-versus-host disease, and true erythrocytosis.^704,705^ Upadacitinib has been approved by the FDA to treat moderate to severe Crohn’s disease in patients with inadequate response or intolerance to TNF medications, moderate to severely active RA in patients with inadequate response or intolerance to methotrexate, patients with active psoriatic arthritis, and patients with non-imaging axial spondyloarthritis.^706–708^ Peficitinib has been approved in Japan to treat RA patients with inadequate response to conventional therapy.^709^ Fedratinib and momelotinib have received FDA approval for the treatment of patients with moderate or high risk of MF.^710,711^ Taken together, JAK inhibitors are widely available for anti-inflammatory treatment due to their effects on inflammation-related signal transduction.

C5a also activates neutrophils, enhancing the inflammatory response and immune function. Targeting C5a-C5aR interactions has shown potential for treating various inflammatory diseases.^712^ Blocking the protein-protein interaction between C5a and C5aR represents a viable strategy for anti-inflammatory therapy.^713^ Avacopan, an orally available C5aR1 inhibitor, is used as an adjunct to glucocorticoids for treating antineutrophil cytoplasmic antibody (ANCA)-associated vasculitis^714^ and is being tested for conditions like atypical hemolytic uremic syndrome (aHUS), IgA nephropathy, and C3 glomerulonephritis. However, avacopan therapy is not recommended for patients with liver disease or cirrhosis.^715^ Its efficacy and safety have been demonstrated in patients receiving rituximab as part of induction therapy for ANCA.^716^ Additionally, antagonizing C5aR1 has been shown to alter microglia polarization and attenuate disease progression in a murine model of Alzheimer’s disease (AD), although clinical trials are still needed.^717^ Avacopan has been approved by the FDA for the adjunctive treatment of severely active ANCA-associated vasculitis (specifically microscopic polyangiitis and granulomatosis with polyangiitis) in adults, in combination with standard therapies, including glucocorticoids.^718^ A phase IV clinical trial (NCT06072482) is currently evaluating the long-term safety and efficacy of avacopan in subjects with ANCA-associated vasculitis.

In summary, significant progress has been made in developing drugs targeting pro-inflammatory factor-binding receptors. Numerous drugs, both approved for treatment and in preclinical studies, have demonstrated substantial anti-inflammatory effects. However, the complete understanding of intracellular signaling pathways regulated by these receptors remains elusive, and blocking receptor binding to pro-inflammatory factors may result in adverse effects beyond the intended anti-inflammatory benefits. This is a primary reason many preclinical drugs have yet to receive approval for clinical use. Thus, optimizing drug structures to reduce adverse effects while enhancing therapeutic efficacy is the current focus in preclinical anti-inflammatory drug development.

#### Drugs modulating inflammatory factors or contents released by neutrophils

In addition to receptor modulators that regulate neutrophil function by targeting membrane receptors, numerous targeted drugs that decrease the levels of inflammatory factors or contents released by neutrophils are in development or already in clinical application. Canakinumab, an anti-IL-1β monoclonal antibody, has been approved for the treatment of FCAS, MWS, CAPS, sJIA, familial Mediterranean fever (FMF), mevalonate kinase deficiency (MKD), TNF receptor-associated periodic syndrome (TRAPS), and AOSD.^677^ A phase II clinical trial (NCT03775109) is exploring the potential benefits of canakinumab for the treatment of alcoholic hepatitis.

Siltuximab, an anti-IL-6 monoclonal antibody, has been approved for the treatment of Castleman’s disease.^719^ A phase II clinical trial (NCT04975555) is currently evaluating the efficacy of siltuximab in managing cytokine release syndrome (CRS) associated with CAR-T cell therapy and immune effector cell-associated neurotoxicity (ICANS). IL-8 is a potent pro-inflammatory chemokine; however, no systemic treatment targeting IL-8 has received approval. In China, an anti-human IL-8 monoclonal antibody cream has been approved for treating inflammatory skin diseases such as psoriasis and eczema.^720^ HuMax-IL8 (BMS-986253), another anti-IL-8 monoclonal antibody, exhibited a favorable safety and tolerability profile in solid tumor patients during a phase I clinical trial (NCT02536469).^721^

Infliximab (Remicade), an intravenous anti-TNF-α monoclonal antibody, has been shown to contribute to the treatment of multiple inflammatory autoimmune diseases, such as IBD, RA, psoriasis, and Kawasaki disease, by specifically binding to and neutralizing TNF-α to inhibit neutrophil recruitment, reducing inflammation and tissue damage.^722–724^ C5 is a bioactive molecule that activates neutrophils and promotes the release of inflammatory mediators. Anti-C5 monoclonal antibody therapies are emerging as promising anti-inflammatory agents. Eculizumab has been approved for treating paroxysmal nocturnal hemoglobinuria (PNH) and aHUS.^725^ Additionally, it has been approved in China for treating adult anti-hydropathin-4 (AQP4) antibody-positive optic neuromyelitis optica spectrum disorder (NMOSD).^726^ Vilobelimab has been approved for treating critically ill patients with COVID-19^727^ and has shown therapeutic benefits in patients with pyoderma gangrenosum and hidradenitis suppurativa.^728,729^ Ravulizumab has received approval for the treatment of anti-AChR antibody-positive myasthenia gravis (gMG), aHUS, and PNH.^730–732^ A phase III clinical trial (NCT04543591) is underway to evaluate the efficacy and safety of ravulizumab for the treatment of thrombotic microangiopathy after hematopoietic stem cell transplantation. In summary, monoclonal antibodies targeting specific pro-inflammatory cytokines represent promising immunosuppressive therapies for autoimmune and inflammatory diseases. Their potential can be explored in combination with other drugs to enhance treatment outcomes across various human diseases, including cancers.

Neutrophils release enzymes from their granules through degranulation. While these enzymes are responsible for eliminating pathogens, they can also exacerbate the inflammation. Consequently, reducing the release of these contents during neutrophil activation may also promote the resolution of inflammation. Sivelestat (ONO-5046), a selective, reversible, and competitive inhibitor of NE, has been shown to contribute to the treatment of acute lung injury/acute respiratory distress syndrome (ALI/ARDS) and coagulation dysfunction by mitigating lung vascular permeability, elevating pulmonary artery pressure, improving lung tissue wet/dry weight ratio, and reducing leukocyte adhesion in the pulmonary capillary bed.^733–735^ AZD5904, an oral irreversible MPO inhibitor, has been shown to contribute to the treatment of chronic obstructive pulmonary disease (COPD) by blocking the progression of emphysema and small airway remodeling.^736,737^ Dornase Alfa (Pulmozyme), a nebulized inhalation recombinant human DNase, has been shown to contribute to the treatment of CF, COPD, and COVID-19 by degrading DNA in lung mucus to remove NETs, thereby reducing mucus viscosity and promoting respiration.^738–740^

Although multiple anti-inflammatory therapeutic agents targeting neutrophils have been developed, with some gaining clinical approval, the progress of many drugs remains hindered in clinical trials or developmental stages. Several factors contribute to the challenges faced by these trials: an incomplete understanding of the spatial and temporal regulation of human neutrophil function and biology, resulting in suboptimal target selection; inadequate dosing regimens; unacceptable adverse effects; and difficulties in translating findings from animal models to humans. As our understanding of neutrophil surface receptors and intracellular signaling pathways continues to advance, anti-inflammatory drugs targeting neutrophils will be refined. Collectively, the development of anti-inflammatory drugs targeting neutrophils presents both opportunities and challenges.

### Anti-tumor therapy

The dual function of neutrophils in the tumor microenvironment poses challenges for tumor immunotherapy. Three therapeutic strategies targeting TANs are currently proposed: (1) blocking neutrophil infiltration into local tumors, (2) inhibiting the immunosuppressive function of neutrophils, and (3) amplifying the anti-cancer activity of neutrophils.^75^ Multiple drugs can act directly or indirectly on neutrophils to achieve these therapeutic goals, including receptor antagonists, monoclonal antibodies, and small molecule drugs targeting transcription factors (Table 3).

**Table 3:** Current therapeutic drugs targeting TANs

Therapeutic Strategy	Target	Drugs	Indication	Clinical Trial	References
Blocking neutrophil infiltration into local tumors	G-CSF1R	LY3022855	Advanced solid cancer	NCT02718911	^744^
	IL-6R	Tocilizumab	Melanoma	NCT03999749	^745^
	IL-8	BMS-986253	Advanced hepatocellular carcinoma	NCT04050462	^721^
	CXCR1/2	SX-682	Metastatic colorectal cancer	NCT04599140	NA
	C5aR	TJ210001	Advanced solid cancer	NCT04947033	^75^
	CXCR4	BL-8040	Pancreatic cancer	NCT02826486	^752^
Inhibiting the immunosuppressive function of neutrophils	Tyrosine kinase	Cabozantinib	Advanced refractory solid tumor	NCT05038839	NA
	STAT3	WP1066	Recurrent malignant glioma	NCT01904123	^755^
		TTI-101	Advanced solid cancer	NCT03195699	^756^
		VVD-130850	Advanced solid cancer	NCT06188208	^757^
	Arginase-1	Arginine	Glioblastoma multiforme	NCT02017249	NA
	Phosphodiesterase 5	Tadalafil	Resectable gastric cancer	NCT05709574	NA
	LXR	RGX-104	Advanced solid cancer	NCT02922764	NA
Amplifying the adjuvant anti-cancer efficacy of neutrophils	CD47	Magrolimab	Hematological malignancy	NCT03248479	^768^
			Advanced solid cancer	NCT02953782	NA
		Lemzoparimab	Hematological malignancy	NCT04912063	^769^
	SIRPα	CC-95251	Advanced solid cancer	NCT03783403	NA
		BI 765063		NCT03990233	NA
	TLR2/4	BCG	Non-muscle invasive bladder cancer	NCT03711032	NA
			Melanoma	NCT00671554	NA
				NCT01838200	NA
	TGF-β receptor type I kinase	Galunisertib	Rectal adenocarcinoma	NCT02688712	^776^
	TGF-β	GC1008	Metastatic breast cancer	NCT01401062	^777^
	TGF-β and PD-L1	M7824	Biliary tract cancer	NCT03833661	^778^

All clinical trials were identified at clinicaltrials.gov

#### Blocking neutrophil infiltration into local tumors

Blocking pathways involved in neutrophil production shows potential to halt cancer progression. Tumor-derived G-CSF drives the activity and generation of immunosuppressive neutrophils within the tumor microenvironment.^741^ Downstream transcription factors activated by G-CSF, including DNA binding inhibitor 1 (DBI-1), retinoblastoma 1 (Rb1), and IRF8, play pivotal roles in driving neutrophil production and promoting their immunosuppressive functions.^202^ G-CSF secreted by breast cancer cells regulates neutrophil activity *via* the JAK/STAT3 pathway, promoting the malignant progression of breast cancer.^742^ In metastatic breast cancer, IL-17 expressed by γδ T cells induces macrophage-derived IL-1β, which in turn regulates G-CSF-dependent neutrophil amplification and polarization. Neutralizing G-CSF prevents neutrophil accumulation and mitigates the T cell suppressive phenotype of neutrophils.^743^ A phase I clinical trial (NCT02718911) evaluated the safety, pharmacokinetics, and anti-tumor activity of LY3022855, a CSF1R inhibitor, in combination with durvalumab or tremelimumab for the treatment of advanced solid cancers.^744^ Additionally, blocking pathways involved in neutrophil recruitment has demonstrated potential for halting cancer progression. Activation of IL-8 through CXCR1/CXCR2, IL-6 through IL-6R, and C5a through C5aR signaling contributes to TAN recruitment.^308^ A phase II clinical trial (NCT03999749) confirmed the efficacy of tocilizumab (anti-IL-6R monoclonal antibody) in combination with ipilimumab and nivolumab in the treatment of unresectable stage III or IV melanoma.^745^ BMS-986253, an anti-IL-8 monoclonal antibody, has demonstrated safety and tolerability in treating various advanced solid tumors.^721^ A phase II clinical trial (NCT04050462) is also evaluating BMS-986253 in combination with nivolumab for advanced hepatocellular carcinoma. SX-682, a CXCR1 and CXCR2 inhibitor, effectively reduces PMN-MDSC infiltration by blocking CXCR2, thereby slowing tumor progression and synergizing with multiple immunotherapeutic modalities.^746–748^ A phase I /II clinical trial (NCT04599140) is currently evaluating the efficacy of SX-682 in combination with nivolumab for metastatic colorectal cancer. C5a secreted by cancer cells upregulates PD-L1 expression to promote tumor immune evasion and induces the formation of NETs to promote tumor metastasis.^749,750^ A phase I clinical trial (NCT04947033) is evaluating the efficacy of TJ210001, an anti-C5aR monoclonal antibody, in treating advanced solid tumors.^75^ The CXCL12/CXCR4 signaling pathway is crucial for neutrophil homing to the bone marrow and is also involved in neutrophil-mediated tumor immunity.^671^ Plerixafor, a CXCR4 inhibitor, inhibits neutrophil infiltration into tumors and holds the potential for anti-angiogenic therapy in colorectal cancer models.^751^ A phase II clinical trial (NCT02826486) confirmed that the CXCR4 inhibitor BL-8040, in combination with pembrolizumab and chemotherapy, attenuates TAN infiltration and enhances cytotoxic CD8^+^ T cell function in pancreatic cancer patients.^752^ Collectively, targeting neutrophil receptors or inflammatory factors to block neutrophil infiltration into local tumors holds significant promise for enhancing tumor immunotherapy.

#### Inhibiting the immunosuppressive function of neutrophils

TANs impair T cell functionality and suppress their activity through releasing active molecules or cytokines with immunosuppressive functions. Inhibitors targeting these molecules contribute to modulating immunosuppressive signaling in TANs. Targeting JAK/STAT3 signaling holds considerable promise for inhibiting the immunosuppressive function of neutrophils.^483^ Cabozantinib, a tyrosine kinase inhibitor, significantly reduces neutrophil infiltration in the tumor microenvironment and its immunosuppressive activity on CD8^+^ and CD4^+^ T cells.^753^ A phase I clinical trial (NCT05038839) is evaluating the efficacy of cabozantinib in combination with pamipanib for advanced refractory solid tumors. AZD9150, a STAT3 antisense oligonucleotide, remodels the suppressive tumor microenvironment and enhances T cell abundance and function in combination with anti-PD-L1 treatment.^754^ Other ongoing clinical trials evaluating the efficacy of STAT3 inhibitors in cancer treatment include WP1066 (NCT01904123), TTI-101 (NCT03195699), and VVD-130850 (NCT06188208).^755–757^ Moreover, PMN-MDSCs express nitric oxide synthase 2 (Nos2) and arginase-1 (Arg-1), depleting L-arginine to inhibit anti-tumor T cell responses and exert immunosuppressive functions.^758^ L-arginine supplementation inhibits tumor growth, reduces intratumoral PMN-MDSC abundance, and increases the expression of CD8^+^ T cells, CD4^+^ T cells, and pro-inflammatory cytokines in mice.^759^ A phase I clinical trial (NCT02017249) has confirmed the efficacy of oral arginine in improving cellular immune function in patients with glioblastoma multiforme by enhancing the functional response of peripheral T cells. Sildenafil or tadalafil are phosphodiesterase 5 inhibitors that stimulate vasodilation to treat erectile dysfunction by disrupting the phosphodiester bond of cyclic GMP (cGMP).^760^ A previous study has shown that sildenafil attenuates tumor growth by downregulating the expression of Nos2 and Arg-1 in PMN-MDSCs in murine tumor models.^761^ Several phase I and II clinical trials have demonstrated that tadalafil reduces the concentration of MDSCs and Tregs and increases the abundance of antigen-reactive CD8^+^ T cells in patients with squamous cell carcinoma of the head and neck, enhancing the tumor immune response.^762,763^ A phase II clinical trial (NCT05709574) is evaluating the ability of tadalafil in combination with neoadjuvant chemotherapy to inhibit MDSCs in patients with resectable gastric cancer.

Other small molecule drugs without anti-PD-1 blocking function exert antitumor effects by inhibiting neutrophil activity to enhance T cell cytotoxic responses. Exenatide, a glucagon-like peptide 1 receptor agonist, promotes anti-tumor CD8^+^ T cell cytotoxic responses in lung and colon cancer by limiting ROS levels to inhibit NETosis.^764^ Palmitoyl-2-citrulloyl-3-acetyl-rocglycerol (PLAG) reduces neutrophil infiltration, increases CD8^+^ T cell infiltration, and inhibits the infiltration of CD4/FoxP3 tumorigenic T cells in the MB49 hormonal mouse model.^765^ RGX-104, an LXR agonist, reduces PMN-MDSC abundance and activates CD8^+^ T cells in hormonal mice, enhancing the sensitivity of anti-PD-1 blockade therapy.^766^ A phase I clinical trial (NCT02922764) is evaluating RGX-104’s inhibition of PMN-MDSC in advanced solid malignancies.

Collectively, small molecule drugs targeting JAK/STAT3/PD-L1 signal transduction or enhancing T cell cytotoxic responses exert antitumor effects by enhancing immune surveillance of tumor cells, inhibiting the immunosuppressive function of neutrophils, and ameliorating immunotherapy-induced drug responsiveness.

#### Amplifying the anti-cancer activity of neutrophils

TANs can exhibit anti-tumor and pro-tumor phenotypes, underscoring the immunotherapeutic potential of amplifying anti-tumor TAN activity or converting pro-tumor TANs to an anti-tumor phenotype. Targeting the CD47-SIRPα interaction may enhance neutrophil-mediated ADCC and thereby amplify the anticancer effects of neutrophils.^767^

CD47 antibodies, such as lemzoparimab (NCT04912063) and magrolimab (NCT03248479), have demonstrated early clinical benefits in treating hematological malignancies.^768,769^ Magrolimab (NCT02953782) has also been expanded for the treatment of advanced solid tumors. The SIRPα inhibitor BI 765063 (NCT03990233) and the anti-SIRPα antibody CC-95251 (NCT03783403) are currently under evaluation in phase I clinical trials for advanced solid tumors. Moreover, TGF-β induces neutrophil polarization towards the N2 phenotype, while IFN I induces neutrophil polarization towards the N1 phenotype.^67,68^ Therefore, activation of IFN I or blockade of TGF-β signaling may enhance the anti-tumor effect of neutrophils. Immune checkpoint blockades (ICBs), particularly anti-PD-1/PD-L1 blockade, have become a core component of tumor immunotherapy in clinical practice. However, due to primary, adaptive, and acquired resistance, only a limited proportion of patients respond well to ICB as monotherapy.^326^ TLR agonists activate neutrophils and promote type I IFN production in the tumor microenvironment.^325^ Although BCG (TLR2/4 agonist) immunotherapy is the gold standard treatment for high-grade, high-risk non-muscle invasive bladder cancer (NMIBC), approximately 30–40% of patients receiving BCG still experience treatment failure.^770^ Research has shown that BCG induces neutrophils to express TNF-associated apoptosis-inducing ligand (TRAIL/Apo-2L), promoting programmed cell death in tumor cells.^771^ Therefore, the combination of TLR agonists and ICBs is a viable strategy for tumor immunotherapy and is currently under investigation in clinical trials. For instance, a phase III clinical trial (NCT03711032) is assessing the efficacy and safety of pembrolizumab (MK-3475) in combination with BCG for NMIBC treatment. Additionally, clinical trials (NCT00671554 and NCT01838200) have evaluated the efficacy of BCG in melanoma. However, while BCG immunotherapy has been shown to inhibit melanoma progression in murine models,^772^ its clinical benefit remains undetermined.^773^

TGF-β is critical for the polarization of TANs towards the N2 phenotype. A previous study demonstrated that the anti-TGF-β antibodies or TGF-β receptor kinase inhibitor SM16 can block TGF-β signaling, eliminating pro-tumorigenic N2 TANs and promoting anti-tumorigenic N1 TANs in murine tumor models.^67^ Subsequently, several studies have also demonstrated that TGF-β blockade is a promising strategy for triggering CD8^+^ T cell infiltration and improving tumor immunotherapy outcomes.^774,775^ Clinical trials have evaluated various agents targeting TGF-β signaling in cancer treatment. For example, a phase II clinical trial (NCT02688712) is evaluating the efficacy of galunisertib, a TGF-β receptor type I kinase inhibitor, in combination with neoadjuvant radiotherapy for rectal adenocarcinoma.^776^ A phase II clinical trial (NCT01401062) demonstrated the efficacy and tolerability of GC1008, a human anti-TGF-β monoclonal antibody, in combination with local radiotherapy in the treatment of metastatic breast cancer.^777^ Furthermore, M7824, a fusion protein that dually inhibits PD-L1 and TGF-β, has demonstrated efficacy in treating biliary tract cancer in phase II clinical trial (NCT03833661).^778^

Unlike traditional radiotherapy and chemotherapy, tumor immunotherapy harnesses the patient’s own immune system to identify, target, and eliminate tumor cells, effectively suppressing tumors while reducing collateral damage to healthy tissues. This approach has placed tumor immunotherapy at the forefront of current cancer treatment. Neutrophils exhibit a dual function in tumor progression, simultaneously facilitating immune evasion and promoting drug resistance within the tumor microenvironment. However, the clinical application of neutrophil-targeted tumor immunotherapy is hindered by challenges such as lack of specificity, short duration of therapeutic effects, and complex crosstalk with other immune cells. Therefore, integrating targeted neutrophil-based therapies with complementary strategies, including chemotherapy and immunotherapy, represents a promising avenue of current research. This approach aims to synergistically abrogate immunosuppressive mechanisms and enhance anti-tumor immune responses.^779^ In summary, tumor immunotherapy targeting neutrophils presents numerous opportunities and challenges warranting further exploration.

### Neutrophil hitchhiking strategy

From a physical standpoint, hitchhiking is a mode of transportation and loading commonly used for the delivery of various nanoparticles or drugs.^780^ Drugs injected directly into the body may be identified by the immune system as foreign invaders and thus be phagocytosed and removed, compromising their efficacy and producing adverse effects. Therefore, loading drugs onto carriers that can evade immune system surveillance to reach the site of pathogenicity is a widely investigated drug delivery strategy. Lipid nanoparticles are currently popular as drug delivery systems, especially in vaccine development, with great biocompatibility and drug loading efficiency.^781^ Neutrophil hitchhiking is emerging as a novel and effective method of drug delivery due to the rapid recognition and attack of pathogens and chemotactic migration in the body, which promise to improve the therapeutic efficacy of drugs.^782^ Compared to traditional nanotransport systems, neutrophils offer better biosafety and specificity, greatly improving drug delivery efficiency and carrier safety.^61^ Researchers encapsulated pioglitazone into bacterial-derived outer membrane vesicles, making it an ideal bait for neutrophil uptake as a means of evading the blood-brain barrier to treat ischemic stroke.^783^ Researchers also attempted to modify a peptide on the nanoparticle surface to enable specific binding to FPRs on the surfaces of neutrophils.^784^ Neutrophil drug delivery systems may display organ toxicity,^785^ making the safety of this strategy questionable. The special drug preparation requirements, individualized treatment due to cellular differences, and potential hazards of excessive inflammation and thrombosis due to neutrophil activation also limit the clinical application of this strategy.^786^ The current research direction should prioritize the specificity of the patient’s own neutrophils to avoid rejection by the body. Culturing obtained neutrophils in vitro to modify them through genetic engineering techniques, such as clustered regularly interspaced short palindromic repeats/CRISPR associated 9 (CRISPR/Cas9), is also a potential option.

Recent studies have revealed that neutrophil-derived exosomes can operate autonomously, independent of their parent cells, and modulate adaptive immune responses by exerting influence over specific cell types.^787^ Exosomes, small extracellular vesicles ranging in size from 30 to 150 nm and secreted by a variety of cell types, have emerged as mediators of intercellular communication, facilitating the transfer of diverse lipids, proteins, and nucleic acids to recipient cells.^788^ Exosomes possess distinct advantages, including their ability to evade phagocytosis, extend circulation time, and penetrate deep tissues, making them highly promising candidates for drug delivery systems.^787^ The composition and function of exosomes are closely related to their original cell, which means that exosomes secreted by neutrophils have the potential to target tumors and inflammation.^789^ Researchers loaded neutrophil exosomes encapsulating VEGF into hydrogels to promote diabetic wound healing.^790^ Neutrophil exosomes encapsulating doxorubicin (DOX) have been employed to traverse the blood-brain barrier, offering a novel strategy for glioma inhibition.^791^ Neutrophil exosomes may carry NE released during neutrophil degradation to degrade the ECM, contributing to the symptoms of COPD.^792^ Neutrophil exosomes have also been found to release soluble factors to induce thrombosis.^793^ RNA and proteins that drive tumor metastasis may exploit exosomes as escape mechanisms, enabling them to evade immune surveillance.^794^ Therefore, therapeutic strategies based on emerging drug delivery systems mediated by neutrophil hitchhiking hold great promise in inflammatory diseases and malignancies. As the next generation of drug delivery systems continues to evolve, more advanced neutrophil-based hitchhiking nanomaterials will emerge. Although many difficulties remain in the clinical application of neutrophil hitchhiking strategies, they represent a new therapeutic direction in disease nanotherapeutics.

### Neutrophil-to-lymphocyte ratio

The neutrophil-to-lymphocyte ratio (NLR), an easily derived parameter from routine blood tests, offers a cost-effective and practical tool for evaluating inflammatory progression and prognosticating outcomes in cancer patients. A predominance of neutrophils, coupled with reduced lymphocyte levels, is characteristic of the nonspecific acute inflammatory response.^795^ Procalcitonin (PCT) and C-reactive protein (CRP) are more sensitive indicators of inflammatory diseases, including autoimmune diseases,^796^ while the NLR is more useful for predicting disease progression. The NLR has been shown to be markedly elevated in the blood of patients with active ulcerative colitis compared with healthy control individuals.^797^ Moreover, the NLR has been considered a possible early predictor of COVID-19.^798^ Alterations in both local and systemic inflammation, as well as disruptions in leukocyte counts and myelopoiesis, are widely recognized as pivotal features of cancer progression.^799^ Emerging evidence highlights a strong association between elevated NLR levels and the activation of the innate immune response, indicating that NLR may serve as a reflection of tumor-driven inflammatory processes.^800,801^ NLR has consistently been regarded as a reliable prognostic marker across various malignancies, irrespective of disease stage or treatment modality. In patients with metastatic colorectal cancer, an elevated NLR (> 5) has been independently correlated with decreased overall survival (OS).^802^ Similarly, in hepatocellular carcinoma (HCC) patients who underwent living-donor liver transplantation, elevated pre-transplant NLR levels were associated with an increased risk of disease recurrence.^800^ The NLR has consistently demonstrated significant prognostic value across various solid tumor types, disease stages, and therapeutic strategies, establishing it as a promising tool for predicting disease outcomes in clinical practice.

Given the remarkable efficacy of current antagonists targeting neutrophil surface receptors or monoclonal antibodies of cytokines that regulate neutrophil function, the design of drugs that act on neutrophils represents a highly promising treatment strategy. Moreover, the roles of the neutrophil hitchhiking strategy and neutrophil-related assays are gaining increasing attention. Thus, therapeutic strategies targeting neutrophils may replace the current standard of treatment for some immune-related diseases in the future.

## Conclusion and perspective

In the field of neutrophil and signal transduction research, several comprehensive reviews have described the biological functions of neutrophils and summarized the components and delivery mechanisms of signal transduction pathways.^17,30,43,75,285,803–805^ In this review, we seek to provide a comprehensive perspective on neutrophil biology, examining how intracellular signal transduction pathways govern neutrophil functional activities, elucidating their role in human diseases, and summarizing emerging treatment strategies aimed at modulating neutrophil activity. This emphasis distinguishes our review from others, aiming to serve as a valuable resource for scholars across diverse disciplines including immunology, clinical medicine, pharmacology, and related fields.

Neutrophil migration depends on cell polarity adjustment and the remodeling of cytoskeletal actin, which is mediated by downstream signaling pathways, including the PI3K and ERK pathways, by the binding of chemoattractants to the corresponding receptors.^145^ Neutrophil polarity adjustment is characterized by the asymmetric organization of the cell, with a distinct leading edge and uropod. This asymmetry is driven by cytoskeletal actin remodeling at the leading edge in response to chemoattractant signals, a process orchestrated by the actin-nucleating function of the Arp2/3 complex. Activation of upstream signaling pathways catalyzes the conversion of PIP2 to PIP3 within the cell membrane, which in turn activates Cdc42 and Rac. These molecules stimulate the Arp2/3 complex, driving actin polymerization and the subsequent formation of pseudopods.^140^ Neutrophils in the environment of multiple complex chemotactic signals, such as “intermediary” signals (LTB_4_ and CXCL8) and “end-target” signals (fMLF and C5a), may rely on the p38 MAPK pathway and PTEN to screen for the precise direction of chemotaxis to ensure accurate arrival at sites of infection or inflammation.^390,446^ Indeed, downstream crosstalk between the PI3K and p38 MAPK pathways may be the key to the differences in the chemotactic responses of neutrophils to chemoattractants. Understanding the signal transduction pathways underlying neutrophil-regulated chemotactic migration is crucial for designing treatment strategies to effectively manipulate neutrophil-targeted migration, thereby promoting the resolution of inflammation. Particularly in sepsis, targeting these pathways to restore neutrophil chemotaxis to inflammatory sites represents a promising approach to managing immune dysfunction. Additionally, manipulating neutrophil chemotactic migration through the modulation of signaling pathways can help alleviate localized excessive inflammatory responses. High-resolution technologies are increasingly capable of visualizing signal transduction within neutrophils, offering valuable insights for the design of treatment strategies aimed at modulating inflammation.

Given the advancements in high-resolution techniques, including CyTOF and single-cell sequencing,^7^ this review seeks to delve into the diversity and heterogeneity of TANs from the perspective of epigenetic regulation and phenotypic analysis. Within the inflammatory tumor microenvironment, neutrophils are activated to produce cytokines that promote tumor cell proliferation. Additionally, they upregulate PD-L1 expression through JAK/STAT/PD-L1 signaling pathway, allowing tumor cells to escape immune surveillance by impairing T cell recognition and killing mechanisms.^806^ Over the past decade, cancer immunotherapy has revolutionized treatment paradigms, driving the development of a wide array of immunotherapeutic agents. Immunotherapy targeting neutrophils has demonstrated efficacy in inhibiting tumor growth and metastasis, effectively mitigating severe toxicities associated with current immunotherapy regimens.^75^ However, the dualistic nature of TANs presents a challenge in the design of tumor immunotherapies targeting neutrophils. It requires the identification of cytomarkers for neutrophils with pro-tumorigenic properties in tumors of different stages, sites, and tissue contexts, hindering current drug development perspectives. Furthermore, due to the intricate nature of the tumor microenvironment, strategies focused solely on inhibiting tumor-promoting phenotypes or inducing transitions to tumor-suppressing neutrophil phenotypes, along with reducing their immunosuppressive capabilities, often prove insufficient in achieving significant clinical benefit.^65^ Thus, neutrophil-targeting strategies may serve as a complementary approach to immunotherapy targeting tumor cells in the short term. However, neutrophil immunotherapy carries significant warnings and risks. Adverse effects may accumulate and surpass tolerable thresholds, with neutropenia emerging as an unavoidable consequence. Overall, tumor immunotherapy targeting neutrophils presents both opportunities and challenges, with advancements in high-resolution technologies paving the way for research in the field.

Despite significant advances in understanding the pathophysiology of inflammatory and autoimmune disorders, the broad suppression of excessive inflammation using nonspecific immunosuppressive agents continues to be the prevailing therapeutic approach for many conditions. Various receptor antagonists or inhibitors, as well as monoclonal antibodies to various pro-inflammatory cytokines, are indeed effective in reducing inflammation. However, more efforts are required to address the wide immunosuppressive effects and volatile side effects of these drugs. An alternative strategy to mitigate chronic inflammation involves promoting the reverse migration of neutrophils away from sites of local tissue damage. However, research on this mechanism is still in its infancy, and it is uncertain whether reverse neutrophil migration that exhibits a strong pro-inflammatory phenotype has deleterious effects, such as distal organ damage or systemic immunosuppression.^807^ Several preclinical drugs targeting TANs have undergone clinical trials, with some showing promising therapeutic benefits. However, neutrophil-targeted tumor immunotherapy continues to face important clinical limitations, including lack of specificity, short duration of action, and complex crosstalk with other immune cells. Emerging therapies based on neutrophil hitchhiking strategies have shown significant benefits in some inflammatory and malignant diseases.^782^ Collectively, neutrophil-targeted therapies have opened up a new direction in the treatment of human diseases and are proving to be promising therapeutics. The integration of multi-omics analyses with high-resolution technologies represents the forefront for future characterization of neutrophil phenotypes and exploration of their functions.

With the development of increasingly sophisticated technologies, neutrophils are proving to be more complex cells than initially anticipated, and increasing evidence is emphasizing their expanding role in health and disease. The chemotactic migration of neutrophils in complex inflammatory environments and the heterogeneity and diversity of neutrophils in cancer leave many questions unresolved. Exploring signal transduction pathways regulating neutrophil chemotactic migration and tumor immunity may contribute to elucidating neutrophil diversity and function in inflammatory diseases and cancer. Understanding signal transduction pathways that regulate chemotactic migration and tumor immunity of neutrophils may contribute to developing targeted therapeutic strategies based on neutrophil mechanisms. Further investigation is needed to explore how the functional properties of neutrophils can be harnessed to drive a significant breakthrough in immunotherapy, while minimizing collateral damage to the host organism.
